# Gestational weight gain and maternal immediate perinatal and postpartum outcomes in low and middle income countries: individual participant data meta-analyses

**DOI:** 10.1136/bmjmed-2025-001558

**Published:** 2026-07-06

**Authors:** Uttara Partap, Janaína Calu Costa, Enju Liu, Ilana R Cliffer, Dongqing Wang, Molin Wang, Sudeer Kumar Nookala, Vishak Subramoney, Brittany Briggs, Ajibola Ibraheem Abioye, Manfred Accrombessi, Seth Adu-Afarwuah, Sheraz Ahmed, Hellen Akurut, Hasmot Ali, Asad Ali, Joao Guilherme Alves, Teddy Andra, Carla Adriane Leal de Araújo, Alemayehu Argaw, Shams Arifeen, Rinaldo Artes, Per Ashorn, Ulla Ashorn, Fereidoun Azizi, Bowen Banda, Ahmed Tijani Bawah, Nita Bhandari, Zulfiqar A Bhutta, Valerie Briand, Elvira Beatriz Calvo, Marly Augusto Cardoso, Marcia Caldas de Castro, Jose Guilherme Cecatti, Gabriela Chico-Barba, Ranadip Chowdhury, Parul Christian, Shalean Collins, Anthony Costello, Kathryn G Dewey, Titus H Divala, Jamille Gregório Dombrowski, Michele Drehmer, Christopher P Duggan, Pratibha Dwarkanath, Alison M Elliott, Davaasambuu Enkhmaa, Daniel Erchick, Guadalupe Estrada-Gutierrez, Frankie J Fair, Henrik Friis, Jimena Fritz, Samira Behboudi Gandevani, Davaasumbuu Ganmaa, Armando García-Guerra, Alison D Gernand, Exnevia Gomo, Austrida Gondwe, Isabel González-Ludlow, Rebecca Grais, Ousmane Guindo, Lotta Hallamaa, Davidson H Hamer, Giles Hanley-Cook, Alain Hien, Lieven Huybregts, Sheila Isanaka, Fyezah Jehan, Gilberto Kac, Richard Kajubi, Abel Kakuru, Margaret P Kasaro, Joanne Katz, Subarna K Khatry, Nancy F Krebs, Pratap Kumar, Anura V Kurpad, Carl Lachat, Tsering Pema Lama, Hector Lamadrid-Figueroa, Hermann Lanou, Anna Lartey, Miriam K Laufer, See Ling Loy, Nur Indrawaty Lipoeto, Laura Beatriz López, Liania Alves Luzia, Abdullah Mahmud, G Arun Maiya, Kenneth Maleta, Dharma S Manandhar, Mark J Manary, Charles Mangani, Claudio Romero Farias Marinho, Hugo Martínez-Rojano, Susana L Matias, Alicia Matijasevich, Elizabeth M McClure, Sotero S Mengue, Joshua D Miller, Sophie E Moore, Marhazlina Mohamad, Hamid Jan Jan Mohamed, Malay Kanti Mridha, Ferdinand M Mugusi, Ameer Muhammad, Alfa Muhihi, T Verenice Muñoz-Rocha, Wilbroad Mutale, Lynnette M Neufeld, Phuong Hong Nguyen, Maria Angélica Antunes Nunes, Maria Ome-Kaius, David Osrin, Noel Patson, Andrea B Pembe, Otilia Perichart-Perera, Karen E Peterson, Zul Premji, Andrew M Prentice, Amado D Quezada-Sánchez, Najeeb Rahman, Preetha Ramachandra, Usha Ramakrishnan, Arjumand Rizvi, Dominique Roberfroid, Patricia Lima Rodrigues, Stephen J Rogerson, Patricia HC Rondó, Daniel E Roth, Reyna Sámano, Naomi M Saville, Maria Inês Schmidt, Katherine EA Semrau, Yasir Shafiq, Saijuddin Shaikh, Bhim P Shrestha, José Roberto Da Silva, Hora Soltani, Sajid Soofi, Rodrigo Medeiros Martorano, Renato Teixeira Souza, Dan J Stein, Jeffrey SA Stringer, Christopher R Sudfeld, Sunita Taneja, Fahimeh Ramezani Tehrani, Tinku Thomas, James M Tielsch, Laéticia Céline Toe, Holger W Unger, Willy Urassa, Juliana dos Santos Vaz, Audêncio Victor, Emily L Webb, Keith P West, Elizabeth Widen, Lesley Workman, Robert O Wright, Lee Shu Fune Wu, Sera L Young, Heather Zar, Lingxia Zeng, Wafaie W Fawzi, Gestational Weight Gain Pooling Project Consortium

**Affiliations:** 1 Department of Global Health and Population, Harvard T H Chan School of Public Health, Boston, MA, USA; 2 Isaac Centre for Public Health, Indian Institute of Science, Bengaluru, India; 3 Department of Epidemiology, School of Public Health, University of São Paulo, São Paulo, Brazil; 4 Institutional Centers for Clinical and Translational Research, Boston Children’s Hospital, Boston, Massachusetts, USA; 5 Division of Gastroenterology, Hepatology, and Nutrition, Boston Children’s Hospital, Harvard Medical School, Boston, Massachusetts, USA; 6 World Vegetable Center, Bangkok, Thailand; 7 Department of Global and Community Health, College of Public Health, George Mason University, Fairfax, Virginia, USA; 8 Department of Epidemiology, Harvard T H Chan School of Public Health, Boston, Massachusetts, USA; 9 Department of Biostatistics, Harvard T H Chan School of Public Health, Boston, MA, USA; 10 Channing Division of Network Medicine, Brigham and Women’s Hospital and Harvard Medical School, Boston, MA, USA; 11 Cytel, India, on behalf of the Bill and Melinda Gates Foundation, Seattle, WA, USA; 12 DVPL Tech, Dubai, UAE; 13 Certara USA, on behalf of the Bill and Melinda Gates Foundation, Seattle, WA, USA; 14 Institut de Recherche Clinique du Benin, Abomey-Calavi, Benin; 15 Department of Nutrition and Food Science, University of Ghana, Legon, Ghana; 16 Aga Khan University, Karachi, Pakistan; 17 MRC/UVRI and LSHTM Uganda Research Unit, Entebbe, Uganda; 18 JiVitA Project, Rangpur, Bangladesh; 19 Department of International Health, Johns Hopkins Bloomberg School of Public Health, Baltimore, MD, USA; 20 Instituto de Medicina Integral Prof Fernando Figueira (IMIP), Recife, Brazil; 21 Infectious Disease Research Collaboration, Kampala, Uganda; 22 Department of Food Technology, Safety and Health, Ghent University, Gent, Belgium; 23 Maternal and Child Health Division, International Centre for Diarrhoeal Disease Research (icddr,b), Dhaka, Bangladesh; 24 Institute of Education and Research (INSPER), Sao Paulo, Brazil; 25 Faculty of Medicine and Health Sciences, Tampere University, Tampere, Finland; 26 Endocrine Research Centre, Research Institute for Endocrine Sciences, Shahid Beheshti University of Medical Sciences, Tehran, Iran; 27 Unit of Environmental Sciences and Management, North West University, Potchefstroom Campus, Potchefstroom, South Africa; 28 Department of Medical Laboratory Sciences, School of Allied Health Sciences, University of Health and Allied Sciences, Ho, Ghana; 29 Centre for Health Research and Development, Society for Applied Studies, New Delhi, India; 30 Centre for Global Child Health, Hospital for Sick Children, Toronto, ON, Canada; 31 Institute for Global Health & Development, Aga Khan University, Karachi, Pakistan; 32 Research Institute for Sustainable Development (IRD) EMR 271, Bordeaux Population Health Centre, National Institute for Health and Medical Research (INSERM) UMR 1219, University of Bordeaux, Bordeaux, France; 33 Former Head, Department of Nutrition, Mother & Child Health Direction, Ministry of Health, Buenos Aires, Argentina; 34 Faculdade de Saúde Pública, Universidade de São Paulo, São Paulo, Brazil; 35 Harvard T H Chan School of Public Health, Boston, Massachusetts, USA; 36 University of Campinas, Campinas, Brazil; 37 Nutrition and Bioprogramming Coordination, Instituto Nacional de Perinatología, Lomas de Virreyes, Mexico City, Mexico; 38 Center for Human Nutrition, Department of International Health, Johns Hopkins Bloomberg School of Public Health, Baltimore, Maryland, USA; 39 Tulane University, New Orleans, Louisiana, USA; 40 UCL Insitute for Global Health, London, UK; 41 Department of Nutrition, University of California, Davis, California, USA; 42 Blantyre Malaria Project, Kamuzu University of Health Sciences, Blantyre, Malawi; 43 Department of Parasitology, Institute of Biomedical Sciences, University of São Paulo, São Paulo, Brazil; 44 Postgraduate Studies Programme in Epidemiology, School of Medicine, Federal University of Rio Grande do Sul, Porto Alegre, Brazil; 45 Division of Gastroenterology, Hepatology and Nutrition, Boston Children’s Hospital, Harvard Medical School, Boston, Massachusetts, USA; 46 Department of Nutrition, Harvard T H Chan School of Public Health, Boston, MA, USA; 47 Division of Nutrition, St John's Research Institute, Bangalore, India; 48 London School of Hygiene and Tropical Medicine, London, UK; 49 National Centre for Maternal and Child Health, Ulaanbaatar, Mongolia; 50 Department of Immunobiochemistry, Instituto Nacional de Perinatología (Mexico City), Mexico City, Mexico; 51 College of Health, Wellbeing and Life Sciences, Sheffield Hallam University, Sheffield, UK; 52 Department of Nutrition, Exercise and Sports, University of Copenhagen, Copenhagen, Denmark; 53 Center for Research in Nutrition & Health, National Institute of Public Health, Cuernavaca, Mexico; 54 Faculty of Nursing and Health Sciences, Nord University, Bodø, Nordland, Norway; 55 Channing Division of Network Medicine, Brigham and Women's Hospital, Boston, Massachusetts, USA; 56 Harvard Medical School, Boston, Massachusetts, USA; 57 Centro de Investigación en Nutrición y Salud, Instituto Nacional de Salud Pública, Cuernavaca, Mexico; 58 Department of Nutritional Sciences, Pennsylvania State University, Philadelphia, PA, USA; 59 Faculty of Medicine & Health Sciences, University of Zimbabwe, Harare, Zimbabwe; 60 UNC Project, Tidziwe Centre, Kamuzu Central Hospital, Lilongwe, Central Region, Malawi; 61 Nutrition and Bioprogramming Coordination, Instituto Nacional de Perinatología, Mexico City, Mexico; 62 Epicentre, Paris, France; 63 Epicentre Niger, Niamey, Niger; 64 Department of Global Health, Boston University School of Public Health, Boston, Massachusetts, USA; 65 Section of Infectious Diseases, Department of Medicine, Boston University Chobanian & Avedisian School of Medicine, Boston, Massachusetts, USA; 66 Center on Emerging Infectious Disease, Boston University, Boston, Massachusetts, USA; 67 Nazi Boni University, Bobo-Dioulasso, Hauts-Bassins, Burkina Faso; 68 Nutrition, Diets, and Health Unit, International Food Policy Research Institute, Washington, DC, USA; 69 Department of Pediatrics and Child Health, Aga Khan University, Karachi, Pakistan; 70 Nutritional Epidemiology Observatory, Josué de Castro Nutrition Institute, Federal University of Rio de Janeiro, Rio de Janeiro, Brazil; 71 UNC Global Projects – Zambia, LLC, Lusaka, Zambia; 72 Nepal Nutrition Intervention Project-Sarlahi, Kathmandu, Nepal; 73 University of Colorado School of Medicine, Aurora, Colorado, USA; 74 Department of Reproductive Medicine and Surgery, Kasturba Medical College, Manipal Academy of Higher Education, Manipal, India; 75 Department of Physiology, St John's Medical College, Bangalore, India; 76 Department of Perinatal Health, National Institute of Public Health, Cuernavaca, Mexico; 77 Institut de Recherche en Sciences de la Sante (IRSS), Ouagadougou, Burkina Faso; 78 Center for Vaccine Development and Global Health, University of Maryland School of Medicine, Baltimore, MA, USA; 79 Duke-NUS Medical School, Singapore; 80 Department of Reproductive Medicine, KK Women’s and Children’s Hospital, Singapore; 81 Department of Nutrition, Andalas University, Padang, Indonesia; 82 Faculty of Medicine, Nutrition School, University of Buenos Aires, Buenos Aires, Argentina; 83 School of Public Health, University of Sao Paulo, Sao Paulo, Brazil; 84 Nutrition and Clinical Services Division, International Centre for Diarrhoeal Disease Research (icddr,b), Dhaka, Bangladesh; 85 Shield Pharmaceuticals, Hauppauge, NY, USA; 86 Department of Physiotherapy, Manipal College of Health Professions, Manipal Academy of Higher Education, Manipal, India; 87 School of Public Health and Family Medicine, University of Malawi, College of Medicine, Blantyre, Malawi; 88 Mother and Infant Research Activities (MIRA), Kathmandu, Nepal; 89 Department of Pediatrics, Washington University, St. Louis, St. Louis, Missouri, USA; 90 Escuela Superior de Medicina del Instituto Politécnico Nacional, Mexico City, Mexico; 91 Department of Nutritional Sciences and Toxicology, University of California, Berkeley, Berkeley, California, USA; 92 Faculdade de Medicina, Universidade de São Paulo, São Paulo, Brazil; 93 RTI International, Durham, NC, USA; 94 Universidade Federal do Rio Grande do Sul, Porto Alegre, Brazil; 95 University of North Carolina Chapel Hill, Chapel Hill, NC, USA; 96 Department of Women and Children’s Health, King’s College London, London, UK; 97 MRC Unit The Gambia at the London School of Hygiene and Tropical Medicine, Fajara, Gambia; 98 School of Nutrition and Dietetics, Faculty of Health Sciences, Universiti Sultan Zainal Abidin, Gong Badak Campus, Kuala Nerus, Malaysia; 99 Nutrition Programme, School of Health Sciences, Universiti Sains Malaysia, Kubang Kerian, Malaysia; 100 Centre for Non-communicable Diseases and Nutrition, BRAC James P Grant School of Public Health, BRAC University, Dhaka, Bangladesh; 101 Department of Internal Medicine, Muhimbili University of Health and Allied Sciences, Dar es Salaam, Tanzania; 102 VITAL Pakistan Trust, Karachi, Pakistan; 103 Africa Academy for Public Health, Dar es Salaam, Tanzania; 104 University of Zambia School of Public Health, Lusaka, Zambia; 105 Food and Agriculture Organization of the United Nations (FAO), Rome, Italy; 106 International Food Policy Research Institute, Washington, DC, USA; 107 Postgraduate Studies Program in Epidemiology, School of Medicine, Federal University of Rio Grande do Sul, Porto Alegre, Rio Grande do Sul, Brazil; 108 Papua New Guinea Institute of Medical Research, Goroka, Papua New Guinea; 109 Malaria Alert Centre, Kamuzu University of Health Sciences, Blantyre, Malawi; 110 Department of Obstetrics and Gynaecology, Muhimbili University of Health and Allied Sciences, Dar es Salaam, Tanzania; 111 Nutrition and Bioprogramming Coordination, Instituto Nacional de Perinatología (Mexico City), Mexico City, Mexico; 112 Nutritional Sciences Department, School of Public Health, University of Michigan, Ann Arbor, Michigan, USA; 113 Department of Parasitology/Medical Entomology, School of Public Health and Social Sciences, Muhimbili University of Health and Allied Sciences, Dar es Salaam, Tanzania; 114 Centro de Investigación en Evaluación y Encuestas, Instituto Nacional de Salud Pública, Cuernavaca, Morelos, Mexico; 115 Department of Physiotherapy, Manipal College of Health Professions, Manipal Academy of Higher Education, Manipal, Karnataka, India; 116 Hubert Department of Global Health, Rollins School of Public Health, Emory University, Atlanta, Georgia, USA; 117 Department of Medicine, Namur University, Namur, Belgium; 118 Belgian Health Care Knowledge Centre, Brussels, Brussels-Capital Region, Belgium; 119 Instituto de Puericultura e Pediatria Martagão Gesteira, Divisão de Nutrição, Universidade Federal do Rio de Janeiro, Rio de Janeiro, Brazil; 120 Department of Infectious Diseases, University of Melbourne, Doherty Institute, Melbourne, VIC, Australia; 121 Department of Pediatrics and the Centre for Global Child Health, The Hospital for Sick Children, Toronto, ON, Canada; 122 University College London Institute for Global Health, London, UK; 123 Ariadne Labs, Brigham & Women's Hospital and Harvard T H Chan School of Public Health, Boston, MA, USA; 124 Centre of Excellence for Trauma and Emergencies and Community Health Sciences, Aga Khan University, Karachi, Pakistan; 125 Global Advancement of Infants and Mothers (AIM), Department of Pediatric Newborn Medicine, Brigham and Women’s Hospital, Harvard Medical School, Boston, MA, USA; 126 Health Research & Develop Forum (HRDF), Kathmandu, Nepal; 127 Instituto de Medicina Integral Prof. Fernando Figueira (IMIP), Recife, Pernambuco, Brazil; 128 Centre of Excellence in Women and Child Health, Aga Khan University, Karachi, Pakistan; 129 Multidisciplinary Centre, Federal University of Acre, Cruzeiro do Sul, Brazil; 130 Department of Psychiatry and Mental Health and SA-MRC Unit on Risk and Resilience, University of Cape Town, Cape Town, South Africa; 131 Department of Obstetrics and Gynecology, University of North Carolina School of Medicine, Chapel Hill, NC, USA; 132 Reproductive Endocrinology Research Center, Research Institute for Endocrine Sciences, Shahid Beheshti University of Medical Sciences, Tehran, Iran; 133 Department of Biostatistics, St John's Medical College, Bangalore, India; 134 Department of Global Health, Milken Institute School of Public Health, George Washington University, Washington, DC, USA; 135 Nutrition and Metabolic Diseases Unit, Health Sciences Research Institute (IRSS), Bobo-Dioulasso, Burkina Faso; 136 Global and Tropical Health Division, Menzies School of Health Research, Charles Darwin University, Casuarina, NT, Australia; 137 Department of Microbiology and Immunology, Muhimbili University of Health and Allied Sciences, Dar es Salaam, Tanzania; 138 Faculty of Nutrition, Universidade Federal de Pelotas, Pelotas, Brazil; 139 School of Human Ecology, University of Texas at Austin, Austin, TX, USA; 140 Department of Paediatrics and Child Health and SA-MRC unit on Child & Adolescent Health, University of Cape Town, Cape Town, South Africa; 141 Department of Environmental Medicine and Public Health, Icahn School of Medicine at Mount Sinai, New York, NY, USA; 142 Northwestern University, Evanston, IL, USA; 143 Department of Epidemiology and Biostatistics, School of Public Health, Xi’an Jiaotong University Health Science Centre, Xi’an, People's Republic of China

**Keywords:** Nutritional sciences, Obstetrics, Public health, Epidemiology

## Abstract

**Objective:**

To estimate the associations between gestational weight gain and maternal immediate perinatal and postpartum outcomes by pooling data from low and middle income countries.

**Design:**

Individual participant data meta-analyses.

**Data sources:**

PubMed, Embase, Web of Science, and Cochrane Library, based on three searches (Search 1: all prospective studies published from January 2000 to May 2021; Search 2: randomized controlled trials of balanced energy and protein supplementation published until June 2021; Search 3: randomized controlled trials of anti-infectious agents published until August 2021).

**Eligibility criteria for selecting studies:**

Prospective studies (randomised controlled trials or observational cohort studies) with measured maternal weight during pregnancy and data available on maternal height, based in populations from low and middle income countries with no underlying conditions.

**Results:**

The analyses included 156 300 women from 61 studies and 23 countries, with most participants based in South Asia (n=78 454, 50.2%) and sub-Saharan Africa (n=36 327, 23.2%). Compared with women with adequate (90-125%) gestational weight gain, women with excessive (>125%) gestational weight gain had a higher risk of caesarean delivery (risk ratio 1.10, 95% confidence interval 1.06 to 1.13, τ^2^=0.000) and emergency caesarean delivery (risk ratio 1.22, 1.03 to 1.43, τ^2^=0.000). Women with moderately (70% to <90%) or severely inadequate (<70%) versus adequate gestational weight gain had lower risks for caesarean delivery (risk ratio in women with moderately inadequate gestational weight gain 0.88, 95% confidence interval 0.84 to 0.92, τ^2^=0.004; risk ratio in women with severely inadequate gestational weight gain 0.82, 0.77 to 0.88, τ^2^=0.010) and emergency caesarean delivery (risk ratio in moderately inadequate gestational weight gain 0.82, 0.71 to 0.95, τ^2^=0.004; risk ratio in severely inadequate gestational weight gain 0.73, 0.56 to 0.96, τ^2^=0.103). Excessive versus adequate gestational weight gain was associated with higher postpartum weight retained at any time point (mean difference 2.00 kg, 95% confidence interval 1.49 to 2.50, τ^2^=1.317), whereas moderately and severely inadequate gestational weight gain were associated with lower retained weight compared with adequate gestational weight gain. Similar trends were found for postpartum body mass index. Severely inadequate gestational weight gain was associated with lower systolic and diastolic blood pressure at any time point post partum than adequate gestational weight gain. No associations were observed for other outcomes including postpartum depressive symptoms or breastfeeding. Evidence indicating an interaction between gestational weight gain and body mass index before pregnancy was found when examining the risk of caesarean delivery and postpartum weight retention, body mass index, and systolic blood pressure as outcomes.

**Conclusions:**

These findings support the association between suboptimal gestational weight gain and adverse maternal outcomes in the immediate perinatal and postpartum periods. Further research examining the consequences of suboptimal gestational weight gain in low and middle income countries would be valuable to inform potential strategies to improve long term maternal health.

**Review registration:**

PROSPERO CRD42023432836

**WHAT IS ALREADY KNOWN ON THIS TOPIC**

- Robust, large scale analyses based in low and middle income countries have shown associations between inadequate or excessive versus adequate gestational weight gain and adverse neonatal outcomes, including low birth weight, small for gestational age, microcephaly, preterm birth, and macrosomia
- Most available data on potential maternal consequences of suboptimal gestational weight gain are from high income countries
- Robust evidence for associations between inadequate or excessive gestational weight gain and maternal perinatal and postpartum outcomes from low and middle income countries is limited

**WHAT THIS STUDY ADDS**

- An increased risk of caesarean and emergency caesarean delivery, and higher postpartum weight retention and body mass index, was associated with excessive versus adequate gestational weight gain
- Clear or consistent associations between gestational weight gain and other immediate perinatal complications, postpartum depression, waist circumference, blood pressure, or breastfeeding difficulty were not found

**HOW THIS STUDY MIGHT AFFECT RESEARCH, PRACTICE, OR POLICY**

- These results indicate important potential maternal health consequences associated with suboptimal gestational weight gain in low and middle income countries
- More robust evidence is needed to confirm these findings and the potential value of interventions that focus on optimising gestational weight gain for improved maternal health in these settings

## Introduction

Gestational weight gain is increasingly recognised as a key potential determinant of pregnancy outcomes. Substantial evidence has indicated associations between suboptimally low or high gestational weight gain and the risk of adverse neonatal outcomes.^1–3^ Specifically, recent large scale analyses based on data pooled from studies in Latin America, Asia, and Africa indicated that severely inadequate gestational weight gain was associated with increased risks of adverse outcomes of up to 40-60%, including low birth weight, small for gestational age, short for gestational age, and microcephaly, with increased risks also observed for moderately inadequate gestational weight gain.^1^ Excessive gestational weight gain was also associated with an increased risk of preterm birth, large for gestational age, and macrosomia.^1^ Because of its modifiable nature, this available evidence indicates that gestational weight gain may be a useful target for strategies aiming to reduce the risk of these outcomes.

Emerging literature also indicates the potential influence of suboptimal gestational weight gain on adverse maternal outcomes at the time of delivery and in the longer term. These adverse outcomes include immediate perinatal outcomes (at the time of delivery), such as caesarean delivery,^4 5^ postpartum measures, such as breastfeeding success,^6^ and longer term outcomes, including postpartum depression,^7^ postpartum weight retention,^8^ and development of cardiometabolic disease.^8–10^ Many of these outcomes are interrelated and have multifactorial determinants and complex development pathways.^11 12^ Recent studies have focused particularly on the relation between excessive gestational weight gain and these outcomes, with effects on immediate perinatal outcomes thought to be mediated by mechanisms relating to the mismatch between fetal and pelvic size.^13^ Effects on other outcomes, including breastfeeding success, are considered to be at least partly a result of endocrine and inflammatory mechanisms related to adiposity.^6 14^ Evidence of the potential maternal consequences of inadequate gestational weight gain is limited, with research suggesting negative associations with measures such as starting breastfeeding.^6^

Much of the available evidence on suboptimal gestational weight gain and adverse maternal outcomes comes from studies conducted in higher income countries, with limited data from low and middle income countries,^15–17^ despite a notable burden of both inadequate and excessive gestational weight gain in these countries.^3 18 19^ Evidence from low and middle income countries about the role of suboptimally low or high gestational weight gain in the development of poor maternal outcomes may help to inform strategies aiming to maintain healthy gestational weight gain, reduce the risk of these outcomes, and promote longer term maternal and child health in these settings. Therefore, collating and considering the available evidence on associations between gestational weight gain and maternal outcomes in a robust manner is needed, particularly for low and middle income countries.

We conducted individual participant data meta-analyses to estimate the association between gestational weight gain and maternal outcomes at delivery and post partum. The individual participant data meta-analyses were based on data pooled from studies measuring maternal pregnancy weight across low and middle income countries in Asia, Africa, and Latin America. We also explored the potential modification of associations with gestational weight gain by body mass index before pregnancy. We considered outcomes such as immediate perinatal complications, psychosocial health, anthropometric and cardiometabolic status, and indicators related to breastfeeding. Considering that a gestational weight gain reference does not currently exist that is representative of populations from low and middle income countries across the range of categories of body mass index before pregnancy, we focused on the adequacy of gestational weight gain defined by the 2009 recommendations of the US Institute of Medicine.^20^

## Materials and methods

### Objectives

The primary objective of this set of individual participant data meta-analyses was to quantify associations between suboptimal gestational weight gain (severely inadequate gestational weight gain, moderately inadequate gestational weight gain, and excessive gestational weight gain *v* adequate gestational weight gain) and maternal immediate perinatal (at the time of delivery) and postpartum outcomes in women living in low and middle income countries. Specifically, we assessed the risk of caesarean delivery, emergency caesarean delivery, perineal tears, postpartum haemorrhage, obstructed or prolonged labour, postpartum depressive symptoms, and any or exclusive breastfeeding at any time post partum. We also recorded mean difference in weight retention, body mass index, waist circumference, and systolic and diastolic blood pressure measured at any time post partum in women with suboptimal versus adequate gestational weight gain. The secondary objective of the analysis was to examine whether associations between gestational weight gain and maternal outcomes might be modified by body mass index before pregnancy (underweight, normal, and overweight or obese).

### Literature search and data acquisition

This set of individual participant data meta-analyses was conducted as part of the Gestational Weight Gain Pooling Project, a data pooling project to understand the distribution, determinants, and consequences of suboptimal gestational weight gain in low and middle income countries. This data pooling project was conducted in two phases (phases 1 and 2). Phase 1 (2019-21) of the Gestational Weight Gain Pooling Project comprised an investigation of gestational weight gain distribution,^18^ sociodemographic and clinical predictors,^19^ nutritional interventions,^21^ and associated neonatal outcomes.^1^ Phase 2 (2021-24) extended this work under a separate funding grant, including updating analyses of gestational weight gain distribution,^22^ and examining additional dietary and psychosocial predictors, nutritional and clinical interventions,^23 24^ and maternal outcomes. Our study was a research aim under phase 2 of the pooling project.

**Figure 1:**
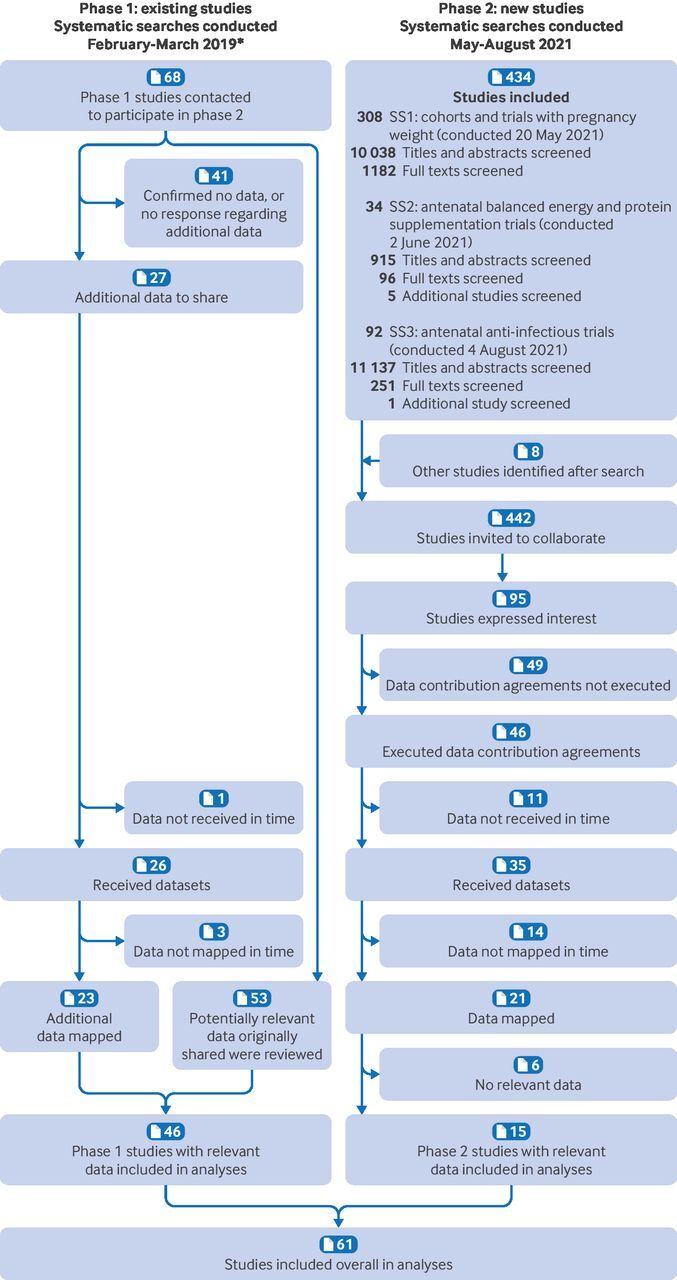
Flowchart of study inclusion for analysis. *The full flowchart for phase 1 has been published previously.^1^ SS= systematic search.

In phase 1 of the Gestational Weight Gain Pooling Project, we conducted systematic literature searches in PubMed, Embase, and Web of Science in February and March 2019, with the final searches conducted by 31 March 2019, restricted to publications on or after 1 January 2000. Eligible studies were prospective studies (randomised controlled trials or observational cohort studies) with measured maternal weight during pregnancy and data available on maternal height, based in populations from low and middle income countries with no underlying conditions (eg, HIV). Retrospective studies analysing maternal weight data from routine medical records were not eligible. The results of the original searches and initial analytical outputs are described in detail elsewhere.^1 19 21^ The main research aims of phase 1 did not include examining maternal outcomes and therefore all of the available relevant data for our analyses were not originally collated from participating investigators. In 2021, we re-contacted all investigators to confirm interest in participating in phase 2, and to request any additional relevant data available to share. Because of funding constraints, we considered all studies in phase 1 of the Gestational Weight Gain Pooling Project where any relevant data could be harmonised (ie, where the required variables were identified and made into consistent format for use across all datasets) before 4 March 2024. In total, we identified and included relevant variables from 46 phase 1 studies (figure 1).

In phase 2 of the Gestational Weight Gain Pooling Project on 20 May 2021, we updated the systematic searches that were conducted in phase 1, based on the same databases as well as the Cochrane Library and with the same eligibility criteria. We also performed two additional systematic searches with related aims. In the first search, performed on 2 June 2021, we identified all randomised controlled trials published so far that examined balanced energy and protein supplementation versus any treatment not involving balanced energy and protein supplementation during pregnancy in low and middle income countries.^23^ In the second search, performed on 4 August 2021, we identified all randomised controlled trials published so far examining antenatal anti-infectious agents in low and middle income countries.^24^ These searches were supplemented with manual searches of relevant systematic reviews and information provided by colleagues on ongoing studies. We contacted all investigators and produced data sharing arrangements for those interested and who had relevant data available. Because of funding constraints, we considered all studies where data sharing agreements could be established, data were shared, and relevant data could be harmonised before 4 March 2024. In total, we included data from 15 phase 2 studies in this analysis (figure 1 and online supplemental table 1).

10.1136/bmjmed-2025-001558.supp1 Supplementary data

### Variables of interest and inclusion of participants

For our analyses, we included women with singleton pregnancies with data for gestational age at each weight measurement and for date of delivery. Data for sex were taken from the included studies rather than from patient reported gender. Participants were pregnant women and other pregnant people. To ensure calculation of the gestational weight gain metric of interest, participants were included who had at least one weight measurement before pregnancy or in early pregnancy (first trimester) and one measurement after the first trimester, or at least one weight measurement in the second trimester. For women with one weight measurement in the second trimester, early pregnancy weight was imputed with validated methods based on mixed effects restricted cubic spline models previously described elsewhere,^25^ grouped by geographic region. This method assumes that missingness is at random, that the subject specific random intercepts and slopes follow a normal distribution, and that the error follows a mean zero normal distribution.^25^ Compared with other simple (eg, arithmetic imputation based on nearest measures) and more complex (eg, marginal models with generalised estimating equations) imputation methods, this method was found to most accurately predict early pregnancy weight in two longitudinal pregnancy cohorts in Tanzania (mean absolute error between observed and imputed values, 1.99 kg and 1.60 kg).^25^

#### Calculation of gestational weight gain

The predictor of interest was total gestational weight gain adequacy ratio, defined according to the criteria of the Institute of Medicine. Briefly, gestational weight gain adequacy was calculated as gestational weight gain observed between weight before pregnancy or in the first trimester and the latest time point in pregnancy as a percentage of gestational weight gain recommended or expected during the same time period, according to the recommendations of the Institute of Medicine.^20^ Box 1 outlines the calculations.^26^

Box 1 **Calculation of gestational weight gain**

- **Observed gestational weight gain**=Last available weight – observed weight before pregnancy or observed or imputed first trimester weight
- **Recommended gestational weight gain by Institute of Medicine 2009**=((Expected first trimester total weight gain by body mass index category/13+6 weeks*) × (13+6 weeks* − gestational age at first measured or imputed weight measure)) + ((gestational age at the last weight measurement – 13+6 weeks*) × Institute of Medicine recommended rate of gestational weight gain for the second and third trimesters by body mass index category)^20^
- **Total gestational weight gain adequacy ratio**=(Observed gestational weight gain/recommended gestational weight gain) × 100%

*Gestational age 13+6 weeks=13 weeks and 6 days.

For calculations and use in analyses, body mass index category was based on the observed weight before pregnancy or observed or imputed first trimester weight. For women aged ≥20 years, body mass index was categorised as underweight (<18.5), normal (18.5-24.9), overweight (25-29.9), or obese (≥30).^27^ For those aged <20 years, body mass index was categorised based on the World Health Organization 2007 reference as underweight (<−2 standard deviations, SD), normal (−2 to <1 SD), overweight (1 to <2 SD), or obese (≥2 SD).^28^

The total gestational weight gain adequacy ratio was categorised as severely inadequate (<70%), moderately inadequate (70% to <90%), adequate (90-125%), or excessive (>125%), as done previously, with adequate gestational weight gain being the referent category.^1 19^ The cut-off values of >125% and <90% reflect the upper and lower limits of recommended gestational weight gain by the Institute of Medicine, and the cut-off value of <70% (severely inadequate) was added to reflect the severity of inadequate gestational weight gain in low and middle income countries, because the recommendations of the Institute of Medicine are based on high income populations.^1 19^ To ensure biological plausibility, we excluded from our analyses participants with a total gestational weight gain adequacy ratio at extremes (study specific ≤1st centile and ≥99th centile).

Because the recommendations of the Institute of Medicine are based on data from high income populations, as part of our secondary analyses, we also examined gestational weight gain adequacy expressed with INTERGROWTH-21st (International Fetal and Newborn Growth Consortium for the 21st Century) standards for gestational weight gain,^29^ where recommended gestational weight gain (denominator) was set as the gestational age specific mean gestational weight gain based on the standard curves. The INTERGROWTH-21st standards for gestational weight gain were constructed only for participants with a normal body mass index before pregnancy, and therefore we restricted our analyses to this subpopulation (with body mass index calculated based on observed weight before pregnancy, or observed or imputed first trimester weight).

#### Outcomes of interest

Outcomes of interest that we originally considered included immediate perinatal complications (ie, complications at the time of delivery, covering caesarean delivery (scheduled and emergency), perineal tears, cephalopelvic disproportion, shoulder dystocia, obstructed and prolonged labour, perinatal asphyxia, postpartum haemorrhage, postpartum infection or sepsis, maternal death, and length of stay at health facility), psychosocial health (postpartum depression, anxiety, or stress), nutritional (including anthropometric measurements such as postpartum weight, body mass index, waist circumference, iron deficiency, and anaemia), cardiometabolic measures (blood pressure, dyslipidaemia, dysglycaemia, diabetes, and metabolic syndrome), inflammatory measures (levels of C reactive protein, alpha(1) acid glycoprotein, and interleukin 6), and indicators related to breastfeeding (starting breastfeeding, length of breastfeeding, and any or exclusive breastfeeding at any time point). Outcomes in these domains have previously been linked with gestational weight gain and often also with body mass index before pregnancy, with some hypotheses on plausible mechanisms of effect.^4–10 13 14^ Studies with extensive missingness on outcome values (defined as n<50 observations and <10% of total sample available) were not considered for pooling (online supplemental tables 2 and 3).

Our aim was to pool data where three or more studies were available for each outcome and therefore outcomes included in the final analyses were: caesarean delivery (either emergency or scheduled, *v* vaginal delivery; n=56 studies), emergency caesarean delivery (*v* vaginal delivery; n=16 studies), perineal tears (observed or abstracted from records by the study team or self-reported by participants; n=8 studies), postpartum haemorrhage (excessive bleeding after birth, as observed or abstracted by the study team or self-reported by participants; n=11 studies), obstructed or prolonged labour (observed or abstracted by the study team or self-reported by participants or, where possible, defined as labour lasting >20-24 hours; n=9 studies), postpartum depressive symptoms (being in the study specific top 20% group *v* lower 80% group for depressive symptom scores, to account for the varying scales used across studies; n=7 studies), postpartum weight retention (defined as the difference between weight at any time point post partum and weight before pregnancy or in the first trimester; n=32 studies), body mass index (n=32 studies) and waist circumference (n=3 studies) measured at any time point post partum, systolic and diastolic blood pressure measured at any time point post partum (n=17 studies), and currently any breastfeeding and exclusively breastfeeding (as binary variables; n=14 and n=10 studies, respectively) at any time point post partum (online supplemental tables 2 and 3). We conducted risk of bias assessments for the included studies with the QUIPS (Quality in Prognosis Studies) risk of bias assessment instrument for prognostic factor studies (online supplemental table 5).^30^

#### Covariates

**Figure 2:**
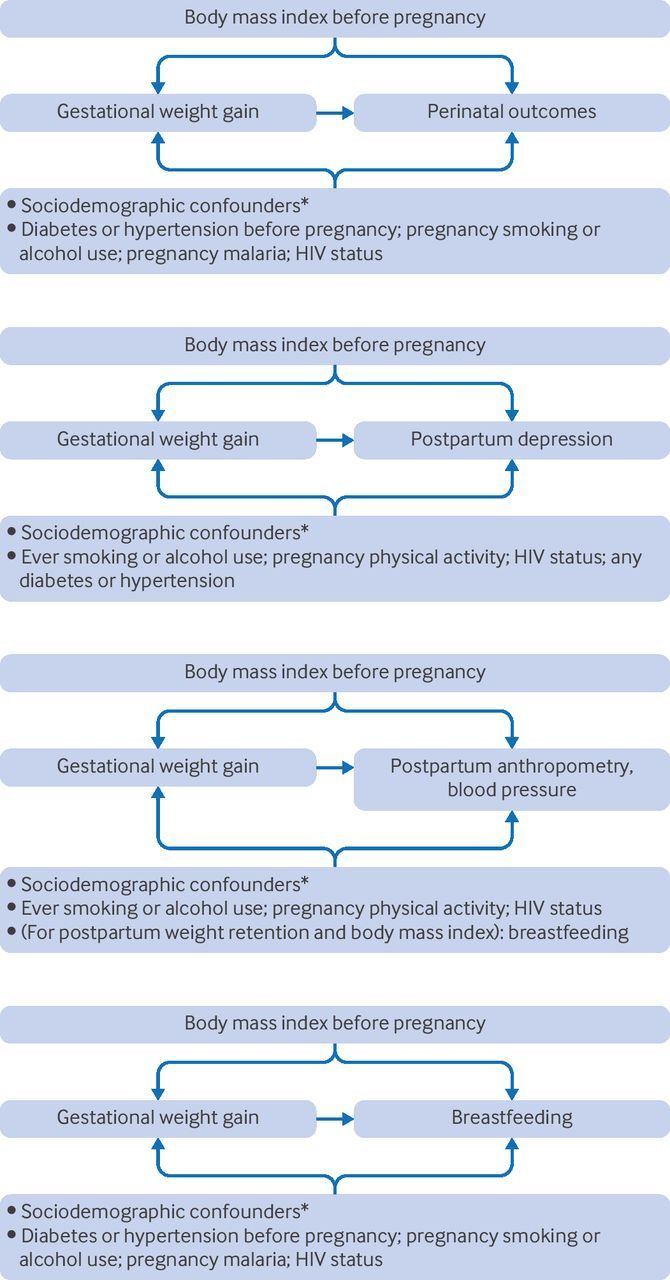
Directed acyclic graphs guiding analytical models. *Sociodemographic (and design related) confounders: maternal age, parity, maternal education and wealth, intervention arm (for trials), and study cluster (random effect)

Based on previous research^4–10 13 14^ and data availability, we developed directed acyclic graphs to guide the selection of potential confounders (figure 2). These potential confounders were adjusted for where possible in all analyses: maternal age, parity, maternal education and wealth, body mass index before pregnancy (based on observed weight before pregnancy, or observed or imputed first trimester weight), intervention arm (for randomised controlled trials), and study cluster (for clustered studies, this variable was included in models as a random effect). For analyses examining immediate perinatal and breastfeeding outcomes, we also adjusted for diabetes or hypertension before pregnancy, alcohol or smoking in pregnancy, malaria in pregnancy, and HIV status, where possible. Analyses examining nutritional and cardiometabolic measures were further adjusted for ever consuming alcohol or smoking, HIV status, and any physical activity at any available time point. Analyses examining postpartum weight retention and body mass index were also adjusted for breastfeeding status at that time point.

For analyses examining systolic and diastolic blood pressure, we excluded participants with known pre-existing hypertension. Models assessing depressive symptoms were further adjusted for ever consuming alcohol or smoking, HIV status, any physical activity in pregnancy, and diabetes or hypertension recorded at any time. Models with measures at multiple time points included number of days post partum as a covariate and were adjusted for clustering by participant identifier. Missing data on covariates, in studies where these were measured, were handled with the missing indicator method.^31^

### Statistical analyses

The primary analysis was a two stage individual participant data meta-analysis because this approach offers greater flexibility in terms of modelling, handling of available confounders, assessing heterogeneity across studies, and dealing with bias in interaction models.^32^ For each meta-analysis, participants with available data on the outcomes of interest were considered for inclusion. Study specific estimates were generated. For single time point measures, Poisson or linear regression models with robust standard errors (or corresponding mixed effects regressions for studies with cluster variables) were constructed. For multiple time point measures, mixed effects Poisson or linear regression models adjusted for clustering at the individual level (and any other study specific clusters) with robust standard errors were constructed. Gestational weight gain was modelled as a categorical predictor (severely inadequate, moderately inadequate, or excessive *v* adequate). Models for each outcome were adjusted for all available relevant confounders, as described above. Study specific estimates were then combined by using random effects meta-analyses (restricted maximum likelihood (REML) method, with Hartung-Knapp adjustment for derivation of 95% confidence intervals). As part of these analyses, we also examined 95% prediction intervals to estimate potential effects in a new study.

Studies with implausible risk ratio point estimates of >10 or <0.01, generally because of low or zero occurrence of the outcome in certain groups, were not included in the meta-analyses (maximum studies excluded in one meta-analysis: n=7 for caesarean delivery, n=2 for emergency caesarean delivery, n=1 for postpartum haemorrhage, and n=1 for prolonged labour). In additional analyses, we checked and confirmed that inclusion of these studies did not substantially affect the estimates. We examined heterogeneity with the τ² statistic. For each meta-analytic model, we checked for evidence of small study bias by examination of funnel plots and Egger's test statistics.

We also explored the potential interaction of gestational weight gain with body mass index before pregnancy by assigning the category specific median values to each category of body mass index and gestational weight gain, and examined these as continuous predictors. This approach was taken to preserve the ordinal nature of the body mass index and gestational weight gain categories in the primary analysis while maintaining power, and has been used previously.^33^ Before undertaking these analyses, we used likelihood ratio tests to check and confirm the assumption of linearity of associations for body mass index and gestational weight gain. We pooled study specific estimates for parameters expressing the multiplicative interaction between these two predictors. Where pooled interaction estimates were significant, we grouped models by the body mass index category before pregnancy (underweight, normal, and overweight or obese, defined according to the thresholds mentioned above) to understand differences in associations.

Finally, we examined the robustness of associations by repeating the primary analyses with gestational weight gain adequacy defined according to the INTERGROWTH-21st standards. We also checked the robustness of the estimates by repeating the primary analyses only in women with measured (ie, non-imputed) weight before pregnancy or first trimester weight, and with complete case analysis to deal with missing data. Also, to examine the robustness of the primary approach used to deal with missing data, we used multiple imputation with chained equations to handle missing data for models examining caesarean delivery. Data were imputed for the outcome (caesarean delivery) and all relevant covariates. Imputation was undertaken separately for each study, and the imputation model included the outcome variable and all covariates. A total of 20 datasets were generated for each study, with information from all datasets pooled to obtain the final study specific estimate that was taken forward for the meta-analysis. We also repeated the meta-analyses only with studies considered to be at overall low risk of bias (defined as having a low risk of bias in all domains assessed, or with a moderate risk of bias in a maximum of one domain). All analyses were conducted with Stata 16 (StataCorp, TX).

### Patient and public involvement

Patients and/or the public were not involved in the design, or conduct, or reporting, or dissemination plans of this research, as it was a secondary analysis based on data from previous studies. Study results will not be sent to individual participants; however they may be disseminated to relevant patient and public communities.

## Results

We included 61 studies with 156 300 participants with data on gestational weight gain, across 23 low and middle income countries (table 1 and online supplemental table 2). Overall, 34.4% (n=21) of studies were based in sub-Saharan Africa, 29.5% (n=18) in Latin America and the Caribbean, and 26.2% (n=16) in South Asia (table 1). Because of differences in sample sizes, the greatest proportion of participants were based in South Asia (n=78 454, 50.2%), followed by sub-Saharan Africa (n=36 327, 23.2%), and North Africa and the Middle East (n=23 867, 15.3%, from one study based in Iran). Five studies with sample sizes of ≥10 000 contributed 55.8% (n=87 272) of all participants.^34–37^

At baseline, 19.6% (n=30 666) of women were aged <20 years and 20.1% (n=31 470) were aged ≥30 years. Of women with data available (n=109 051), 57.2% (n=62 360) had completed 0-7 years of education. Most women had a normal body mass index before pregnancy or in the first trimester, with 16.2% (n=25 338) classified as having underweight and 19.9% (n=31 159) as having overweight or obesity. We found severely inadequate gestational weight gain in 34.5% (n=53 860) of all women, moderately inadequate gestational weight gain in 21.6% (n=33 773), and excessive gestational weight gain in 20.2% (n=31 521) (table 1). First trimester weight was imputed for 50 623 (32.4%) women. Online supplemental tables 2-4 describe more study specific characteristics. The overall risk of bias across the included studies was generally low. Three (4.9%) studies were classified as having a high risk of bias in one domain and six (9.8%) studies as having a moderate risk of bias in two domains. Twenty two (36.1%) of studies were considered to have a low risk of bias across all domains assessed (online supplemental table 5).

**Table 1:** Characteristics of included studies (n=61) and participants (n=156 300) considered in analyses

	Studies	Participants
World region		
Latin America and the Caribbean	18 (29.5)	9900 (6.3)
North Africa and the Middle East	1 (1.6)	23 867 (15.3)
South Asia	16 (26.2)	78 454 (50.2)
South East Asia, East Asia, and Oceania	5 (8.2)	7752 (5.0)
Sub-Saharan Africa	21 (34.4)	36 327 (23.2)
Maternal age (years)		
<20		30 666 (19.6)
20-24		54 526 (34.9)
25-29		39 638 (25.4)
≥30		31 470 (20.1)
Maternal education (years)		
0-4		39 206 (25.1)
5-7		23 154 (14.8)
8-11		30 738 (19.7)
≥12		15 953 (10.2)
Missing		47 249 (30.2)
Body mass index before pregnancy or in first trimester		
Underweight		25 338 (16.2)
Normal		99 803 (63.9)
Overweight		23 017 (14.7)
Obese		8142 (5.2)
Gestational weight gain adequacy*		
Severely inadequate (<70%)		53 860 (34.5)
Moderately inadequate (70% to <90%)		33 773 (21.6)
Adequate (90% to 125%)		37 146 (23.8)
Excessive (>125%)		31 521 (20.2)

Data are number (%).

*Defined as ratio of observed gestational weight gain versus gestational weight gain recommended in line with 2009 guidelines from the Institute of Medicine.

Data on maternal outcomes of interest were available across distinct subsets of studies. The largest number of studies and participants was available for caesarean delivery (56 studies, 102 929 participants) and other immediate perinatal outcomes, with fewer studies available for postpartum outcomes, such as depressive symptoms and waist circumference (table 2). Among participants with available data, the proportion of women having caesarean or emergency caesarean delivery was 23.8% and 8.6%, respectively, and 11.3% of participants had perineal tears. Postpartum haemorrhage was observed or reported for 20.3% of participants (with this relatively high value from one study based on self-report^36^), and prolonged labour for 15.5% of participants. Postpartum measures were typically taken within the first year after delivery. For all participants with data available on depressive symptoms, most were taken at one time point, a median of 84 (interquartile range (IQR) 50) days post partum, and 10.5% of participants were classified as being in the highest study specific group (group 5, scores categorised as five equal groups) of symptom scores at any time. Based on multiple observations across participants, average values for postpartum body mass index, waist circumference, and blood pressure were within normal ranges. Almost all women were breastfeeding across all time points (93.2%, measured a median of 89 (IQR 125) days post partum), with 42.0% exclusively breastfeeding measured at a median of 105 (IQR 140) days post partum (table 2).

**Table 2:** Summary¶ of available data on perinatal and postpartum outcomes of interest (n=61 studies)

	No (%) of studies	No of participants with available data	No of observations (where repeated measures)	No (%) with outcome, or mean (SD) of outcome	Time point of measure (days post partum) (median (IQR))
No (%) of perinatal outcomes					
Caesarean delivery†	56 (91.8)	102 929		24 481 (23.8)	
Emergency caesarean delivery‡	16 (26.2)	24 475		2106 (8.6)	
Perineal tears	8 (13.1)	46 136		5197 (11.3)	
Postpartum haemorrhage	11 (18.0)	48 978		9927 (20.3)	
Prolonged labour	9 (14.8)	54 150		8413 (15.5)	
No (%) of psychosocial measures					
Highest group for depressive symptom score§	7 (11.5)	19 417	21 540	2264 (10.5)	84 (50)
Postpartum weight and related measures					
Mean (SD) postpartum weight retained (kg)	32 (52.5)	63 214	153 017	1.7 (7.4)	84 (141)
Mean (SD) body mass index	32 (52.5)	63 214	153 017	21.9 (4.8)	84 (141)
Mean (SD) waist circumference (cm)	3 (4.9)	825	1712	87.7 (11.3)	44.5 (68)
Mean (SD) systolic blood pressure (mm Hg)	17 (27.9)	23 302	56 913	107.1 (14.5)	99 (222)
Mean (SD) diastolic blood pressure (mm Hg)	17 (27.9)	23 281	56 845	69.2 (8.7)	99 (222)
No (%) currently breastfeeding					
Any breastfeeding	14 (23.0)	43 194	144 241	134 425 (93.2)	89 (125)
Exclusively breastfeeding	10 (16.4)	29 915	128 854	54 129 (42.0)	105 (140)

*For repeated measures, summaries are based on all observations (non-unique participants).

†Caesarean delivery includes both emergency and scheduled caesarean delivery.

‡Denominator for emergency caesarean delivery includes all deliveries (vaginal delivery, and emergency and scheduled caesarean delivery).

§Depressive symptom scores were measured with the Edinburgh Postnatal Depression Scale (three studies and subset of one study), Patient Health Questionnaire 9 (one study), Centre for Epidemiologic Studies-depression scale (one study), Self-Report Questionnaire 20 (subset of one study), and a study specific tool (one study). Scores categorised as five equal groups (20% each of sample) within each study.

¶Summary measures are based on observations with available data on gestational weight gain adequacy.

IQR, interquartile range; SD, standard deviation.

Compared with women having adequate gestational weight gain, women with excessive gestational weight gain had a 10% increased risk of caesarean delivery (risk ratio 1.10, 95% confidence interval (CI) 1.06 to 1.13, τ^2^=0.000) (table 3 and online supplemental figures 1-3). In contrast, women with moderately and severely inadequate versus adequate gestational weight gain had lower risks of caesarean delivery (risk ratio for moderately inadequate gestational weight gain 0.88, 95% CI 0.84 to 0.92, τ^2^=0.004; risk ratio for severely inadequate gestational weight gain 0.82, 0.77 to 0.88, τ^2^=0.010). We observed similar trends for emergency caesarean delivery (compared with adequate gestational weight gain, risk ratio for excessive gestational weight gain 1.22, 95% CI 1.03 to 1.43, τ^2^=0.000; risk ratio for moderately inadequate gestational weight gain 0.82, 0.71 to 0.95, τ^2^=0.004; risk ratio for severely inadequate gestational weight gain 0.73, 0.56 to 0.96, τ^2^=0.103). We found no clear evidence for associations between gestational weight gain and the risk of perineal tears, postpartum haemorrhage, prolonged labour, or depressive symptoms in the postpartum period, although severely inadequate versus adequate gestational weight gain was associated with a modest decrease in the risk of postpartum haemorrhage (table 3 and online supplemental figures 4-18).

**Table 3:** Associations between suboptimal gestational weight gain versus adequate gestational weight gain and maternal perinatal and postpartum outcomes

	No of studies	No of participants or observations*	No of participants with event†	Risk ratio or mean difference (95% CI)	τ^2^
**Severely inadequate gestational weight gain**					
Perinatal outcomes (risk ratio)					
Caesarean delivery‡	55	53 135	10 270	0.82 (0.77 to 0.88)	0.010
Emergency caesarean delivery	15	13 719	1026	0.73 (0.56 to 0.96)	0.103
Perineal tears	8	29 962	3290	0.95 (0.88 to 1.02)	0.000
Postpartum haemorrhage	11	31 389	7412	0.91 (0.86 to 0.96)	0.000
Prolonged labour	9	37 867	5928	0.82 (0.63 to 1.06)	0.050
Psychosocial measures (risk ratio)					
Highest group for depressive symptom score§	7	12 416	1343	0.99 (0.94 to 1.04)	0.000
Postpartum weight and related measures (mean difference)					
Postpartum weight retained (kg)	32	99 021		−2.82 (−3.47 to −2.17)	2.257
Body mass index	32	99 021		−1.16 (−1.40 to −0.92)	0.326
Waist circumference (cm)	3	870		−3.05 (−8.14 to 2.05)	1.744
Systolic blood pressure (mm Hg)	17	30 449		−1.15 (−2.03 to −0.28)	1.515
Diastolic blood pressure (mm Hg)	17	30 421		−0.58 (−0.96 to −0.20)	0.056
Currently breastfeeding (risk ratio)					
Any breastfeeding	14	97 586	91 046	1.00 (1.00 to 1.00)	0.000
Exclusively breastfeeding	10	89 484	36 587	0.99 (0.95 to 1.03)	0.001
**Moderately inadequate gestational weight gain**					
Perinatal outcomes (risk ratio)					
Caesarean delivery‡	54	48 047	10 638	0.88 (0.84 to 0.92)	0.004
Emergency caesarean delivery	15	12 249	978	0.82 (0.71 to 0.95)	0.004
Perineal tears	8	21 069	2592	0.90 (0.78 to 1.03)	0.012
Postpartum haemorrhage	10	22 081	3757	0.97 (0.91 to 1.04)	0.000
Prolonged labour	9	23 219	3648	1.01 (0.94 to 1.09)	0.000
Psychosocial measures (risk ratio)					
Highest group for depressive symptom score§	7	10 405	981	0.98 (0.93 to 1.03)	0.000
Postpartum weight and related measures (mean difference)					
Postpartum weight retained (kg)	32	69 595		−1.13 (−1.40 to −0.85)	0.391
Body mass index	32	69 595		−0.81 (−1.00 to −0.62)	0.193
Waist circumference (cm)	3	734		−1.98 (−5.89 to 1.92)	1.722
Systolic blood pressure (mm Hg)	17	30 481		−0.80 (−1.43 to −0.16)	0.322
Diastolic blood pressure (mm Hg)	17	30 453		−0.47 (−1.05 to 0.11)	0.182
Currently breastfeeding (risk ratio)					
Any breastfeeding	14	61 693	57 987	1.00 (1.00 to 1.00)	0.000
Exclusively breastfeeding	10	53 252	24 074	0.99 (0.95 to 1.03)	0.000
**Excessive gestational weight gain**					
Perinatal outcomes (risk ratio)					
Caesarean delivery‡	50	55 379	17 539	1.10 (1.06 to 1.13)	0.000
Emergency caesarean delivery	14	10 190	1202	1.22 (1.03 to 1.43)	0.000
Perineal tears	8	16 069	2175	1.06 (0.93 to 1.21)	0.008
Postpartum haemorrhage	11	17 438	1636	0.99 (0.50 to 1.97)	0.608
Prolonged labour	9	13 092	1935	1.03 (0.86 to 1.22)	0.000
Psychosocial measures (risk ratio)					
Highest group for depressive symptom score§	7	10 639	1040	1.04 (0.98 to 1.11)	0.000
Postpartum weight and related measures (mean difference)					
Postpartum weight retained (kg)	32	50 410		2.00 (1.49 to 2.50)	1.317
Body mass index	32	50 410		0.96 (0.70 to 1.23)	0.299
Waist circumference (cm)	3	948		0.85 (−0.56 to 2.26)	0.000
Systolic blood pressure (mm Hg)	17	28 801		0.36 (−0.92 to 1.65)	1.740
Diastolic blood pressure (mm Hg)	17	28 773		1.12 (−0.49 to 2.74)	7.368
Currently breastfeeding (risk ratio)					
Any breastfeeding	14	44 064	40 874	1.00 (1.00-1.00)	0.000
Exclusively breastfeeding	10	34 792	16 512	0.99 (0.96 to 1.03)	0.000

Gestational weight gain adequacy was defined as the ratio of observed gestational weight gain versus gestational weight gain recommended in the 2009 guidelines from the Institute of Medicine. Severely inadequate gestational weight gain was defined as gestational weight gain adequacy ratio <70%, moderately inadequate as 70% to <90%, adequate as 90-125%, and excessive as >125%.

Estimates pooled with the random effects meta-analysis (restricted maximum likelihood (REML) method), with Hartung-Knapp adjustment for standard errors.

*Number of participants or number of observations for repeated measures (postpartum depressive symptoms, weight and related measures, and breastfeeding), for each comparison.

†Number of participants with event where risk ratio is reported.

‡Caesarean delivery includes both emergency and scheduled caesarean delivery.

§Scores categorised as five equal groups within each study.

CI, confidence interval.

For postpartum weight and related outcomes, women with moderately and severely inadequate gestational weight gain had lower weight retention at any postpartum time point than women having adequate gestational weight gain (mean difference for severely inadequate gestational weight gain −2.82 kg, 95% CI −3.47 to −2.17, τ^2^=2.257; mean difference for moderately inadequate gestational weight gain −1.13 kg, −1.40 to −0.85, τ^2^=0.391), whereas women with excessive gestational weight gain retained on average 2.00 kg more weight (95% CI 1.49 to 2.50, τ^2^=1.317) versus those with adequate gestational weight gain (table 3 and online supplemental figures 19-21). We observed similar differences for postpartum body mass index: compared with adequate gestational weight gain, excessive gestational weight gain was associated with higher postpartum body mass index (mean difference 0.96, 95% CI 0.70 to 1.23, τ^2^=0.299), whereas moderately and severely inadequate gestational weight gain were associated with progressively lower body mass index (mean difference for severely inadequate gestational weight gain −1.16, −1.40 to −0.92, τ^2^=0.326).

Relative to women with adequate gestational weight gain, those with moderately and severely inadequate gestational weight gain had a lower postpartum systolic blood pressure (mean difference for moderately inadequate gestational weight gain −0.80 mm Hg, 95% CI −1.43 to −0.16, τ^2^=0.322; mean difference for severely inadequate gestational weight gain −1.15 mm Hg, −2.03 to −0.28, τ^2^=1.515), with similar trends observed for postpartum diastolic blood pressure (table 3 and online supplemental figures 22-33). Gestational weight gain was not found to be associated with waist circumference, or with any breastfeeding or exclusively breastfeeding at any time point (table 3 and online supplemental figures 34-39). When examining associations with 95% prediction intervals, significance was achieved only for associations between severely inadequate versus adequate gestational weight gain and postpartum haemorrhage, and excessive versus adequate gestational weight gain and caesarean or emergency caesarean delivery (online supplemental table 6).

**Figure 3:**
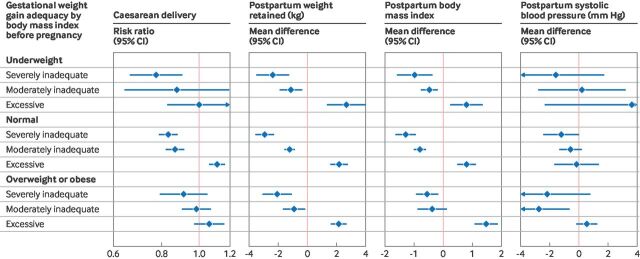
Associations between suboptimal gestational weight gain and key maternal perinatal and postpartum outcomes, grouped by maternal body mass index before pregnancy. Gestational weight gain adequacy was defined as ratio of observed gestational weight gain versus gestational weight gain recommended in line with 2009 guidelines from the Institute of Medicine. Severely inadequate gestational weight gain was defined as gestational weight gain adequacy ratio <70%, moderately inadequate as 70% to <90%, adequate as 90-125%, and excessive as >125%

Pooled estimates for interaction indicated that the associations between gestational weight gain and risk of caesarean delivery, postpartum weight retention, postpartum body mass index, and postpartum systolic blood pressure were modified by body mass index category before pregnancy (online supplemental table 7). Severely inadequate versus adequate gestational weight gain was significantly associated with a reduced risk of caesarean delivery only among those with underweight or a normal body mass index before pregnancy. Moderately inadequate versus adequate gestational weight gain was associated with a reduced risk of caesarean delivery, and excessive versus adequate gestational weight gain was associated with an increased risk of caesarean delivery, only among those with a normal body mass index. Associations were not significant for women with overweight or obesity measured before pregnancy (figure 3 and online supplemental table 8). Associations between severely inadequate, moderately inadequate, and excessive gestational weight gain and postpartum weight retained were largely similar across all body mass index categories. Similar trends were observed when examining postpartum body mass index as the outcome (figure 3 and online supplemental table 8). Conversely, we found no clear evidence of differences in associations between gestational weight gain and systolic blood pressure across body mass index categories.

Estimates of association and trends observed remained substantially unchanged when using gestational weight gain adequacy expressed with INTERGROWTH-21st standards for women with a normal body mass index before pregnancy (online supplemental table 9), when analyses were restricted to participants with measured weight before pregnancy or in the first trimester (online supplemental tables 10 and 11) and when complete case analysis was used (online supplemental table 12), and when multiple imputation was used to deal with missingness in models examining caesarean delivery (online supplemental table 13 and supplemental figures 40-42). Generally, we found no clear evidence of small study bias, except for models examining the relation between severely inadequate gestational weight gain and systolic blood pressure, and for all models examining prolonged labour (online supplemental table 14). Estimates across all meta-analyses remained generally unchanged when our analysis was restricted to studies considered to be at low overall risk of bias (online supplemental table 15).

## Discussion

### Principal findings

Suboptimal gestational weight gain, including inadequate or excessive gestational weight gain, is an important predictor of maternal outcomes in the perinatal and postpartum periods, but evidence from low and middle income countries has been limited. In our set of individual participant data meta-analyses based on more than 150 000 women across 61 studies in 23 low and middle income countries, we found associations between suboptimal gestational weight gain and immediate perinatal and postpartum maternal outcomes. The risks of caesarean and emergency caesarean delivery were higher among women with excessive gestational weight gain versus those with adequate gestational weight gain, and lower among women with moderately and severely inadequate gestational weight gain. Women with excessive gestational weight gain were also more likely to retain more weight and have a higher body mass index post partum than those with adequate gestational weight gain. Moderately and severely inadequate gestational weight gain were associated with lower retained weight and postpartum body mass index.

We observed signs of lower postpartum systolic and diastolic blood pressure among women with moderately and severely inadequate gestational weight gain. Conversely, we did not observe clear associations between gestational weight gain and perineal tears, postpartum haemorrhage, prolonged labour, postpartum depressive symptoms, waist circumference, or breastfeeding status. The evidence indicated that the association between gestational weight gain adequacy and risk of caesarean delivery, postpartum weight retention, body mass index, and systolic blood pressure was modified by body mass index category before pregnancy. Hence these findings indicate potentially important associations between gestational weight gain and the risk of adverse maternal outcomes at delivery and in the longer term.

### Comparison with other studies

Previous research has examined associations between suboptimal gestational weight gain and increased risk of immediate perinatal complications, although these studies were often based in high income or upper middle income countries and focused mainly on excessive gestational weight gain. These associations included increased risk of caesarean delivery,^2 13 38 39^ perineal tears,^13 39^ obstructed labour,^5^ and postpartum haemorrhage.^38 39^ Apart from caesarean delivery, the totality of evidence has remained somewhat less consistent about the association between excessive gestational weight gain and these outcomes.^2 5 13 38 39^ Fetal size is hypothesised as a potentially plausible mechanism for the observed associations, with excessive gestational weight gain leading to larger fetal size relative to maternal pelvic size (including by pathways such as gestational diabetes mellitus^40^), resulting in complications during labour and the potential need for an emergency caesarean delivery.^13^ Anticipated complications in labour potentially associated with excessive gestational weight gain may also result in scheduled caesarean deliveries, as has been noted for gestational diabetes mellitus.^41^ Additional mechanisms have also been proposed, including the potential role of increased pelvic soft tissues narrowing the birth canal.^38^

Excessive gestational weight gain has been linked to fetal macrosomia,^1^ in turn associated with uterine atony,^42^ a risk factor for postpartum haemorrhage.^43^ In line with this prior evidence, we observed an increased risk of caesarean delivery and emergency caesarean delivery among women with excessive gestational weight gain, which may be explained by the pathways described above. In stratified models, the increased risk of caesarean delivery with excessive gestational weight gain was mainly found in those who had a normal body mass index before pregnancy, suggesting an increased need to focus on this subgroup as potentially at higher risk. Reduced sample sizes in stratified models, however, limited definitive conclusions. Also, consistent with previous research,^39^ we also observed trends for a decreased risk of caesarean delivery with inadequate gestational weight gain. This finding might be explained by smaller fetal size relative to maternal pelvic size, which may reduce the risk of complications during labour and associated risk for emergency caesarean delivery, compared with having a fetus of normal size. We, however, reiterate the previously observed strong associations between moderately and severely inadequate gestational weight gain and a range of adverse neonatal outcomes, including low birth weight, small for gestational age, and microcephaly.^1^ These associations indicate the strong negative potential influences on several other pregnancy related outcomes, highlighting the greater overall costs to maternal and child health of insufficient weight gain during pregnancy. No conclusive evidence of associations between inadequate or excessive gestational weight gain and other immediate perinatal outcomes was found from these analyses.

Similarly, the relation between gestational weight gain and postpartum weight retention, body mass index, and waist circumference has more often been examined in higher income settings, with strong evidence indicating positive associations, including up to at least three years post partum.^8–10 13 44 45^ Our analysis provides further evidence from low and middle income countries supporting these data. Measures of adiposity, including weight and body mass index, are strongly associated with blood pressure, hypothesised to be caused by a range of biological mechanisms, including through influences on the sympathetic nervous system, renin-angiotensin-aldosterone and natriuretic peptides systems, and hyperinsulinaemia.^46 47^ Consistent with these hypotheses, we observed similar trends for postpartum systolic and diastolic blood pressure across categories of gestational weight gain adequacy as for postpartum weight retention and body mass index, although the positive associations between excessive gestational weight gain and postpartum blood pressure were not significant.

Associations between excessive gestational weight gain and higher postpartum anthropometric measures likely reflect maternal protein and fat mass accretion during pregnancy.^20^ Furthermore, excessive gestational weight gain in early pregnancy is linked to an increased risk of gestational diabetes mellitus,^48^ which in turn has been associated with increases in cardiometabolic risk in the postpartum period, including higher anthropometric measures, hypertension, dysglycaemia, and type 2 diabetes.^49 50^ Our analysis provides further evidence from low and middle income countries supporting this finding, and because we did not exclude women with gestational diabetes mellitus, may at least partly reflect these pathways. With evidence of links between overweight and obesity and risk of cardiovascular disease and diabetes, including in women in middle age and post partum,^51 52^ these results suggest an increased risk of potentially longer term negative maternal cardiometabolic health associated with excessive gestational weight gain, especially important in women with overweight or obesity before pregnancy who may already have a higher risk of adverse outcomes.

Considering the J shaped association between body mass index and blood pressure, and adverse long term outcomes, the clinical significance of a one unit increase in these measures (as approximately observed in our analysis) would depend on initial body mass index or blood pressure, and may be particularly important for women with overweight or obesity before pregnancy.^53 54^ Furthermore, with nutrition transitions and increasing average body mass index and blood pressure among women, including women of reproductive age,^55 56^ these results indicate potentially important implications in terms of cardiometabolic risk in later life. The potential importance of preventing excessive gestational weight gain among women with higher overweight or obesity before pregnancy is also supported by the slightly greater magnitude of positive associations between gestational weight gain and postpartum body mass index in this group versus women with a normal body mass index or with underweight. These differences were not substantial, however, suggesting that all women may have potential to benefit from interventions aimed at preventing excessive gestational weight gain.

The prevalence of underweight among women of reproductive age in low and middle income countries is substantial,^57 58^ and our findings suggested a potential need to more comprehensively explore long term suboptimal nutritional status in women with severely inadequate gestational weight gain, including features of maternal nutritional depletion, which may affect both future pregnancies and longer term maternal health.^53 59^ Furthermore, although inadequate gestational weight gain was associated with lower postpartum weight retained, body mass index, and blood pressure, previous evidence indicating potentially negative neonatal outcomes associated with inadequate gestational weight gain is important to consider to fully understand the risks associated with lower than optimal gestational weight gain.^1–3^

Excessive gestational weight gain could contribute to the development of depressive symptoms through pathways arising from increased adiposity in the postpartum period (eg, dysregulation of the hypothalamic-pituitary-adrenal axis, inflammatory mechanisms associated with adiposity, or other mechanisms related to body image), which may lead to adverse cognitive and affective changes.^60 61^ Previous evidence has also indicated an increased risk of postpartum depression in those with inadequate gestational weight gain, possibly because of underlying mechanisms related to hunger or food insecurity.^7 60 62^ Our analysis did not indicate associations between suboptimal gestational weight gain and postpartum depressive symptoms, possibly because of the smaller analytical sample sizes, heterogeneity of scales used, and setting specific validity of the scales used to measure depressive symptoms. Although we examined study specific scores, meta-analyses covered four distinct subjective scales, and a clumped distribution of scores in studies was observed in the highest group, comprising <20% of the observations. These factors may have contributed to imprecise estimates. Future large scale studies examining the relation between gestational weight gain and postpartum depressive symptoms based on consistent and validated scales are needed.

Previous evidence suggested that a high body mass index before pregnancy and gestational weight gain may have negative effects on breastfeeding. This finding may be a result of multiple pathways, including increased risk of perinatal complications which may prevent breastfeeding, metabolic effects,^63^ or difficulties with latching and lactation that are associated with adiposity.^6 15 64 65^ Although associations between inadequate gestational weight gain and starting breastfeeding have been reported,^6^ no clear evidence for underlying mechanisms was found. We found no evidence of associations between suboptimal gestational weight gain and breastfeeding in our analysis, suggesting no independent effect compared with other determinants.^66^ Methodological heterogeneity across studies, however, particularly for the specific questions asked to determine exclusive breastfeeding, may also at least partly explain the lack of associations observed.

We found an increased risk of caesarean delivery of about 10% and an increased risk of emergency caesarean delivery of about 20% associated with excessive gestational weight gain. The magnitude of the increase in risk, particularly for emergency caesarean delivery, may be considered clinically important, particularly because of the evidence indicating an increased likelihood of adverse short and long term outcomes associated with caesarean delivery.^67^ Although our analyses were observational and causality must first be confirmed, our findings indicate that preventing excessive gestational weight gain in low and middle income countries may be important in reducing the risk of caesarean delivery in these populations. This implication may be particularly relevant for women with a normal body mass index before pregnancy, among whom stratified models indicated some evidence of an increased risk. The evidence provided in these models, however, may not be enough to warrant differential recommendations for women in different categories of body mass index before pregnancy.^55^ The burden of excessive gestational weight gain is notable in low and middle income countries,^18 19^ alongside increasing body mass index,^55 56^ with multiple barriers to accessing quality, comprehensive emergency obstetric care in these settings—reiterating the potential importance of this finding.^68^ Dietary and physical activity approaches in pregnancy may be useful.^69^ These approaches must consider appropriate balancing of intervention intensity and monitoring of gestational weight gain to ensure that inadequate gestational weight gain is avoided, particularly considering the previously established associations between inadequate gestational weight gain and suboptimal neonatal outcomes.^1–3^

Experimental data based on low and middle income countries indicate a potentially important effect of strategies targeting diet and physical activity for excessive gestational weight gain on reducing the risk of caesarean delivery in women with overweight.^69^ Experimental evidence from low and middle income countries is scarce, however, and further research would be valuable in establishing causality and exploring potentially suitable preventive strategies.^2^ Although our results also indicate associations between excessive gestational weight gain and increases in common measures of cardiometabolic risk, longer term data on a more comprehensive set of risk factors are needed to better understand potential implications. These data could include measures related to the development of type 2 diabetes, because excessive gestational weight gain in early pregnancy has been associated with the risk of gestational diabetes mellitus,^48^ which is linked to later occurrence of type 2 diabetes.^49^ Regardless, strong evidence exists indicating that suboptimal gestational weight gain is associated with poor neonatal and other outcomes, including low birth weight,^1–3^ which are associated with poor long term health.^70^ Our analysis adds to this overall evidence supporting the need to attain optimal gestational weight gain for improved maternal and offspring health in low and middle income countries.

### Strengths and limitations of this study

Our study had some limitations. Although our meta-analyses were based on a large pool of studies and participants, we could not include several studies initially identified as part of our systematic searches for multiple reasons, ranging from lack of response from contacted investigators to data sharing arrangements not being completed on time relative to the end of funding for the project. Because of the observational nature of the data, residual confounding is possible, including for analyses exploring longer term outcomes. Although we adjusted for socioeconomic measures, such as maternal education and wealth, residual confounding cannot be excluded entirely for some associations, particularly those observed between moderately and severely inadequate gestational weight gain and reduced risk of caesarean delivery. In analyses examining caesarean and emergency caesarean delivery, we also did not have data available to account for access to obstetric care. In general, because the analyses covered studies based in a range of settings from low and middle income countries in multiple geographic regions, heterogeneity may have existed across several factors that we could not investigate more closely because of data limitations, including differences in: access to obstetric care; sociocultural practices related to breastfeeding and exclusive breastfeeding; practices related to pregnancy and postpartum diet, and exercise and weight monitoring; and cultural perspectives on depression and interpretation of questions related to depressive symptoms. In particular, we did not have specific information on algorithms of care for different settings in relation to perinatal complications, especially caesarean delivery. This information would be valuable to more clearly distinguish clinical pathways explaining associations, such as decisions by healthcare providers for scheduled caesarean delivery or advice on weight management, versus biological mechanisms, such as fetal size leading to a potentially increased or reduced risk of complications at labour and subsequent emergency caesarean delivery. These factors may have important influences on gestational weight gain adequacy and the risk of later outcomes, thus potentially affecting associations, and should be examined closely in future studies.

Analyses were restricted to participants with information available to calculate the gestational weight gain metrics of interest. We did not undertake imputation for missing data, including for outcomes and covariates. In a limited sensitivity analysis examining caesarean delivery, we observed no substantial difference in estimates when dealing with missing data with multiple imputation with chained equations, suggesting that our approach of the use of the missing indicator and complete case analysis methods to deal with missing data was relatively robust. We cannot fully exclude potential bias, however, if data may be missing not at random for all outcomes, particularly with the missing indicator method or complete case analysis approaches.^71^ First trimester weight was imputed for a substantial proportion of women, and although we observed consistent results in sensitivity analyses restricted to women with measured weight before pregnancy or in the first trimester, we cannot completely exclude errors in weight imputation.

Follow-up was generally limited to the first one or two years post partum, with limited longer term data. Bias from selective attrition may not be entirely excluded, especially for longer term data. Furthermore, self-report of some measures of interest, particularly immediate perinatal variables, such as postpartum haemorrhage, may have reduced the accuracy of the measurement, with potential effects on the strength of associations observed. We also found heterogeneity in the scales used to define postpartum depressive symptoms, which may have affected the findings. Data for some immediate perinatal outcomes were less common than for specific outcomes, such as caesarean delivery or postpartum weight. Although generally we found no clear evidence for small study bias in these cases, Egger's test statistics were significant for prolonged labour, and the smaller sample size for these outcomes may have limited statistical power. This finding indicates the need for more systematic collection and reporting of these measures to understand immediate perinatal health in relation to gestational weight gain.

We examined gestational weight gain adequacy and body mass index as categorical variables to enable interpretation for clinical and public health impact. This approach is limited statistically, including loss of information, and hence future analyses should investigate these variables as continuous measures, and examining linearity of associations with maternal outcomes would be informative. We used two stage meta-analytical approaches to obtain pooled estimates, the assumptions of which may be unreliable when most studies have sparse outcome events.^72^ This approach may have affected estimates of a limited number of meta-analytic models examining immediate perinatal outcomes with fewer overall studies. We also could not more closely examine potential differences in associations for suboptimal gestational weight gain in early versus late pregnancy, or changing trajectories in gestational weight gain adequacy across pregnancy including in response to recommendations from clinical providers (eg, for gestational diabetes mellitus or scheduled caesarean delivery) because of reduced data availability.

We could not examine the relation between specific components of gestational weight gain (eg, gain in weight or body mass index of the mother *v* other components, such as the placenta) and the outcomes of interest. Investigating this relation would more clearly separate the underlying pathways so we can understand to what extent weight at delivery (or specific components of weight gain) may explain the associations observed. These analyses would require more complex statistical considerations to more formally investigate and quantify mediation by these characteristics. Because our primary question was establishing associations of gestational weight gain with these outcomes, and because weight at delivery was not always available in the studies included, we could not examine this question comprehensively in our analysis. Furthermore, detailed data from low and middle income countries, including weight at delivery, and quantifying specific components of weight gain (eg, measuring placental weight) would be valuable to explain these aspects. Discussions have taken place on the need to better define optimal and suboptimal gestational weight gain in low and middle income countries, including the need for references more representative of low and middle income countries.^73^ More comprehensive data would provide a clearer exploration of appropriate references and cut-off values to define suboptimal gestational weight gain in low and middle income countries.

Our analysis had several strengths. Analyses were based on a pool of large scale individual participant data from 23 low and middle income countries across five global regions, with relevant variables harmonized across all studies, and substantial sample sizes for most outcomes. Where associations were observed, the consistency in trends of associations across gestational weight gain categories suggested that these were not because of chance alone. Furthermore, our results are in line with previous literature, mainly from higher income countries examining the influence of suboptimal gestational weight gain on perinatal and postpartum maternal outcomes.^4–10 13 14^

### Conclusions

Our findings support associations between gestational weight gain and the development of adverse maternal outcomes in the immediate perinatal and postpartum periods. Further research examining the later consequences of suboptimal gestational weight gain in low and middle income countries would be beneficial to inform the potential value and design of strategies to improve maternal health in the long term.

## Data Availability

Data may be obtained from a third party and are not publicly available. The datasets analysed during the study are not publicly available because the study was an analysis of data pooled from multiple individual studies. Data from individual studies may be available to share upon reasonable request to the individual study investigators.
