# Supplementary material for: Gestational weight gain and maternal immediate perinatal and postpartum outcomes in low and middle income countries: individual participant data meta-analyses
Source: BMJ Med. 2026 Jul 6;5(1):e001558. doi: 10.1136/bmjmed-2025-001558 (PMC13358349; doi:10.1136/bmjmed-2025-001558)
Supplement: Supplementary data [file bmjmed-5-1-s001.pdf]

BMJ Publishing Group Limited (BMJ) disclaims all liability and responsibility arising from any reliance placed on this supplemental material which has been supplied by the author(s)

Gestational weight gain and maternal perinatal and postpartum outcomes in low- and middle-income countries: an individual participant data meta-analysis

Supplementary material

Supplementary Table 1. Distribution of studies identified in Gestational Weight Gain Pooling Project Phase 2 systematic searches (N=442), not included in current analysis (N=427), and included in current analysis (N=15) across regions.

	n (%) out of total, not included, or included		
**Region**	**Total identified**	**Not included in current analysis**	**Included in current analysis**
Central Europe, Eastern Europe, and Central Asia	5 (1.1)	5 (1.2)	0 (0.0)
Latin America and Caribbean	43 (9.7)	39 (9.1)	4 (26.7)
North Africa and Middle East	29 (6.6)	29 (6.8)	0 (0.0)
South Asia	87 (19.7)	82 (19.2)	5 (33.3)
Southeast Asia, East Asia, and Oceania	107 (24.2)	107 (25.1)	0 (0.0)
Sub-Saharan Africa	161 (36.4)	155 (36.3)	6 (40.0)
Mixed Regions*	10 (2.3)	10 (2.3)	0 (0.0)

Non inclusion may have been for a variety of reasons, including non-response of author to initial invitation, no relevant data to the current analysis on maternal outcomes, inability to establish data sharing agreement on time, or inability to intake and harmonize data on time. The flow of studies from identification to inclusion is outlined in Figure 1.
*Mixed-region studies included: South Asia and Sub-Saharan Africa (2 studies), South Asia, Sub-Saharan Africa and Latin America (5 studies), South and Southeast Asia (1 study), Southeast Asia and Latin America (1 study), and Sub-Saharan Africa and Latin America (1 study)

Supplementary Table 2. Summary of studies included in pooled analysis.

					Gestational weight gain adequacy (n, %)			
**Study acronym**	**Author and date**	**Country**	**Study type**	**Analytical N (participants)**	**Severely inadequate (<70%)**	**Moderately inadequate (70% to <90%)**	**Adequate (90% to <125%)**	**Excessive (≥125%)**
AKU_MMN	Bhutta 2009 (1)	Pakistan	Trial	1507	750 (49.8)	440 (29.2)	219 (14.5)	98 (6.5)
AKU_VITD	Khan 2016 (2)	Pakistan	Trial	532	115 (21.6)	131 (24.6)	159 (29.9)	127 (23.9)
Calcium_India	Dwarkanath 2021 (3)	India	Trial	10472	627 (6.0)	2320 (22.2)	4268 (40.8)	3257 (31.1)
Calcium_Tanzania	Dwarkanath 2021 (3)	Tanzania	Trial	9954	1783 (17.9)	1906 (19.1)	3047 (30.6)	3218 (32.3)
EU_MMN	Ramakrishnan 2003 (4)	Mexico	Trial	353	85 (24.1)	59 (16.7)	129 (36.5)	80 (22.7)
FU_GWG	Rodrigues 2010 (5)	Brazil	Observational	146	22 (15.1)	15 (10.3)	41 (28.1)	68 (46.6)
FU_LEPTINGWG	Franco-Sena 2016 (6)	Brazil	Observational	232	38 (16.4)	24 (10.3)	58 (25.0)	112 (48.3)
GWU_MINT	Erchick 2023 (7)	Nepal	Trial	698	323 (46.3)	146 (20.9)	161 (23.1)	68 (9.7)
ICDDR_MINIMat	Persson 2012 (8)	Bangladesh	Trial	3482	2040 (58.6)	770 (22.1)	572 (16.4)	100 (2.9)
ILINS_DYAD_G	Adu-Afarwuah 2015 (9)	Ghana	Trial	1115	302 (27.1)	199 (17.8)	300 (26.9)	314 (28.2)
ILINS_DYAD_M	Ashorn 2015 (10)	Malawi	Trial	1321	550 (41.6)	409 (31.0)	278 (21.0)	84 (6.4)
IMIP_BRAMAG	de Araujo 2020 (11)	Brazil	Trial	863	82 (9.5)	120 (13.9)	234 (27.1)	427 (49.5)
IMIP_GestDM	do Nascimento 2019 (12)	Brazil	Observational	506	122 (24.1)	135 (26.7)	121 (23.9)	128 (25.3)
INPer_CAR	Samano 2021 (13)	Mexico	Observational	398	89 (22.4)	58 (14.6)	104 (26.1)	147 (36.9)
INPer_FICA	Samano 2017 (14)	Mexico	Observational	164	23 (14.0)	31 (18.9)	47 (28.7)	63 (38.4)
INPer_NeuroObesity	Unpublished	Mexico	Observational	274	94 (34.3)	25 (9.1)	45 (16.4)	110 (40.1)
INPer_Poli	Chico-Barba 2024 (15)	Mexico	Observational	160	41 (25.6)	26 (16.3)	41 (25.6)	52 (32.5)
INPer_REDES	Samano 2018 (16)	Mexico	Observational	327	55 (16.8)	52 (15.9)	109 (33.3)	111 (33.9)
INSPM_ELEMENT	Perng 2019 (17)	Mexico	Observational	732	75 (10.2)	112 (15.3)	220 (30.1)	325 (44.4)
INSPM_PROGRESS	Colicino 2021 (18)	Mexico	Observational	926	32 (3.5)	111 (12.0)	277 (29.9)	506 (54.6)
IRD_RECIPAL	Accrombessi 2018 (19)	Benin	Observational	253	102 (40.3)	47 (18.6)	67 (26.5)	37 (14.6)
JHU_NOMS	Katz 2024 (20)	Nepal	Trial	19411	9031 (46.5)	5295 (27.3)	4123 (21.2)	962 (5.0)
JiVitA3	West 2014 (21)	Bangladesh	Trial	23568	14674 (62.3)	5377 (22.8)	3228 (13.7)	289 (1.2)
MRCG_ENID	Moore 2012 (22)	The Gambia	Trial	804	463 (57.6)	148 (18.4)	135 (16.8)	58 (7.2)
LSHTM_Uganda	Webb 2011 (23)	Uganda	Trial	1278	48 (3.8)	851 (66.6)	169 (13.2)	210 (16.4)
MAHE_SCFPPP	Ramachandra 2017 (24)	India	Observational	68	5 (7.4)	8 (11.8)	19 (27.9)	36 (52.9)
MAL1	Etheredge 2015 (25)	Tanzania	Trial	1340	318 (23.7)	302 (22.5)	316 (23.6)	404 (30.1)
MAL2	Darling 2017 (26)	Tanzania	Trial	1829	633 (34.6)	273 (14.9)	458 (25.0)	465 (25.4)

Supplementary Table 2. Summary of studies included in pooled analysis.

					Gestational weight gain adequacy (n, %)			
**Study acronym**	**Author and date**	**Country**	**Study type**	**Analytical N (participants)**	**Severely inadequate (<70%)**	**Moderately inadequate (70% to <90%)**	**Adequate (90% to <125%)**	**Excessive (≥125%)**
MDIG	Roth 2018 (27)	Bangladesh	Trial	1257	282 (22.4)	316 (25.1)	394 (31.3)	265 (21.1)
MINA_Brazil	Cardoso 2020 (28)	Brazil	Observational	1301	251 (19.3)	197 (15.1)	386 (29.7)	467 (35.9)
MISAME_1	Roberfroid 2008 (29)	Burkina Faso	Trial	1084	709 (65.4)	253 (23.3)	106 (9.8)	16 (1.5)
MISAME_2	Huybregts 2009 (30)	Burkina Faso	Trial	1110	732 (65.9)	226 (20.4)	132 (11.9)	20 (1.8)
MISAME_3	Argaw 2023 (31)	Burkina Faso	Trial	1688	836 (49.5)	349 (20.7)	374 (22.2)	129 (7.6)
MNIPH_ENISD	Neufeld 2019 (32)	Mexico	Trial	412	25 (6.1)	70 (17.0)	138 (33.5)	179 (43.4)
NNIPS_3	Christian 2003 (33)	Nepal	Trial	2889	1972 (68.3)	550 (19.0)	340 (11.8)	27 (0.9)
NWU_PMPEN	Widen 2019 (34)	Kenya	Observational	202	65 (32.2)	63 (31.2)	43 (21.3)	31 (15.3)
NWU_PreNAPS	Widen 2017 (35)	Uganda	Observational	235	116 (49.4)	57 (24.3)	44 (18.7)	18 (7.7)
PNS	Fawzi 2007 (36)	Tanzania	Trial	7385	1937 (26.2)	2016 (27.3)	2072 (28.1)	1360 (18.4)
PROMOTE_BirthCohort3	Kajubi 2019 (37)	Uganda	Trial	662	432 (65.3)	142 (21.5)	76 (11.5)	12 (1.8)
ROSE	Isanaka 2019 (38)	Niger	Trial	2136	1199 (56.1)	435 (20.4)	362 (16.9)	140 (6.6)
SAS_WINGS	Taneja 2022 (39)	India	Trial	4455	1655 (37.1)	886 (19.9)	1161 (26.1)	753 (16.9)
SBUMS_GDM	Tehrani 2019 (40)	Iran	Trial	23867	2606 (10.9)	2764 (11.6)	6463 (27.1)	12034 (50.4)
SHU_BMIGWG	Soltani 2017 (41)	Indonesia	Observational	533	173 (32.5)	94 (17.6)	138 (25.9)	128 (24.0)
SPAZ_IPTp	Unger 2015 (42)	Papua New Guinea	Trial	1908	353 (18.5)	383 (20.1)	777 (40.7)	395 (20.7)
St_Johns	Unpublished (St-Johns)	India	Observational	1557	255 (16.4)	272 (17.5)	515 (33.1)	515 (33.1)
ARGGWG_CURVES	Calvo 2009 (43)	Argentina	Observational	1067	197 (18.5)	130 (12.2)	294 (27.6)	446 (41.8)
UCL_LBWSAT	Saville 2018 (44)	Nepal	Trial	2504	925 (36.9)	1217 (48.6)	302 (12.1)	60 (2.4)
MIRA_Janakpur	Osrin 2005 (45)	Nepal	Trial	1109	528 (47.6)	314 (28.3)	224 (20.2)	43 (3.9)
UC_RDNS	Matias 2016 (46)	Bangladesh	Trial	3344	1740 (52.0)	952 (28.5)	549 (16.4)	103 (3.1)
UHAS_AHPI	Yeboah 2017 (47)	Ghana	Observational	282	29 (10.3)	29 (10.3)	90 (31.9)	134 (47.5)
UMB_CMP	Divala 2018 (48)	Malawi	Trial	758	379 (50.0)	151 (19.9)	147 (19.4)	81 (10.7)
USM_PregCohort	Loy 2014 (49)	Malaysia	Observational	150	40 (26.7)	34 (22.7)	32 (21.3)	44 (29.3)
USP_MatStress	Rondo 2003 (50)	Brazil	Observational	901	136 (15.1)	171 (19.0)	302 (33.5)	292 (32.4)
USP_VIMIP	Dombrowski 2019 (51)	Brazil	Observational	503	124 (24.7)	77 (15.3)	144 (28.6)	158 (31.4)
UZ_MatNutri	Friis 2004 (52)	Zimbabwe	Trial	416	30 (7.2)	223 (53.6)	81 (19.5)	82 (19.7)
VITAL_MUMTA_PW	Muhammad 2022 (53)	Pakistan	Trial	1116	504 (45.2)	250 (22.4)	219 (19.6)	143 (12.8)

Supplementary Table 2. Summary of studies included in pooled analysis.

					Gestational weight gain adequacy (n, %)			
**Study acronym**	**Author and date**	**Country**	**Study type**	**Analytical N (participants)**	**Severely inadequate (<70%)**	**Moderately inadequate (70% to <90%)**	**Adequate (90% to <125%)**	**Excessive (≥125%)**
WomenFirst	Hambidge 2019 (54)	Guatemala	Trial	1120	525 (46.9)	240 (21.4)	223 (19.9)	132 (11.8)
XJU_RuralChina	Zeng 2008 (55)	China	Trial	4147	1547 (37.3)	782 (18.9)	1192 (28.7)	626 (15.1)
XJU_Tibet	Kang 2017 (56)	China	Trial	1014	321 (31.7)	246 (24.3)	290 (28.6)	157 (15.5)
ZAPPS_3	Castillo 2019 (57)	Zambia	Observational	1565	298 (19.0)	258 (16.5)	421 (26.9)	588 (37.6)
ZamCAT	AMANHI 2021 (58)	Zambia	Observational	910	417 (45.8)	236 (25.9)	170 (18.7)	87 (9.6)

Gestational weight gain (GWG) adequacy was defined as ratio of observed GWG versus GWG recommended in line with Institute of Medicine 2009 guidelines.

Supplementary Table 3. Summary of studies included in pooled analysis (additional study details).

Study acronym	Author and date	Study type	Study aim or intervention type	Eligible N[1]	Enrolled N	Analytical N
AKU_MMN	Bhutta 2009 (1)	Trial	Multiple micronutrients vs. iron and folic acid	2438	2378	1507
AKU_VITD	Khan 2016 (2)	Trial	Vitamin D vs. placebo	-	-	532
Calcium_India	Dwarkanath 2021 (3)	Trial	Calcium 500 mg vs. 1500 mg	11310	11000	10472
Calcium_Tanzania	Dwarkanath 2021 (3)	Trial	Calcium 500 mg vs. 1500 mg	11382	11000	9954
EU_MMN	Ramakrishnan 2003 (4)	Trial	Multiple micronutrients vs. iron	921[2]	873	353
FU_GWG	Rodrigues 2010 (5)	Observational	Examine potential determinants of sub-optimal weight gain and associations of gestational weight gain with adverse pregnancy outcomes	292	255	146
FU_LEPTINGWG	Franco-Sena 2016 (6)	Observational	Association between leptin concentrations or other maternal characteristics, by pre-pregnancy BMI	-	299	232
GWU_MINT	Erchick 2023 (7)	Trial	Fortified balanced energy and protein supplementation vs. no supplementation	-	-	698
ICDDR_MINIMat	Persson 2012 (8)	Trials	Early vs. usual supplementation with food and multiple micronutrients, or 30 mg Iron + 400 mcg folate, or 60 mg Fe + 400 ug folate	4940	4436	3482
ILINS_DYAD_G	Adu-Afarwuah 2015 (9)	Trial	Multiple micronutrients vs. lipid-based nutrients vs. iron and folic acid	1926	1575	1115
ILINS_DYAD_M	Ashorn 2015 (10)	Trial	Multiple micronutrients vs. lipid-based nutrients vs. iron and folic acid	4861	1391	1321
IMIP_BRAMAG	de Araujo 2020 (11)	Trial	Magnesium vs. placebo	1019	911	863
IMIP_GestDM	do Nascimento 2019 (12)	Observational	Assess relation between early pregnancy physical activity and risk of gestational diabetes mellitus	907	907	506
INPer_CAR	Samano 2021 (13)	Observational	Examine associations between disordered eating behaviors, gestational weight gain and offspring size in pregnant adolescents	-	-	398
INPer_FICA	Samano 2017 (14)	Observational	Examine associations between prenatal leptin concentrations, gestational weight gain, postpartum weight retention, and newborn size in adolescent pregnancies	-	168	164
INPer_NeuroObesity	Unpublished	Observational	Unspecified	-	-	274
INPer_Poli	Chico-Barba 2024 (15)	Observational	Understand association between inflammatory markers and gestational weight gain in adolescents	317	313	160
INPer_REDES	Samano 2018 (16)	Observational	Evaluate association between pre-pregnancy BMI and gestational weight gain and neonatal outcomes for adolescent mothers	800	601	327

Supplementary Table 3. Summary of studies included in pooled analysis (additional study details).						
**Study acronym**	**Author and date**	**Study type**	**Study aim or intervention type**	**Eligible N[1]**	**Enrolled N**	**Analytical N**
INSPM_ELEMENT	Perng 2019 (17)	Observational	Understand potential effects of lead mobilization from maternal bone stores in pregnancy, and if calcium supplementation can suppress this	3998	997	732
INSPM_PROGRESS	Colicino 2021 (18,19)	Observational	Examine associations between 15 maternal prenatal phthalate metabolite concentrations and children's behavioral problems	-	1054	926
IRD_RECIPAL	Accrombessi 2018 (20)	Observational	Assess consequences of malaria during first trimester of pregnancy	1214	411	253
JHU_NOMS	Katz 2024 (21)	Trial	Topical application of sunflower seed oil vs. mustard seed oil for neonatal mortality and infection status	39602	39479	19411
JiVitA3	West 2014 (22)	Trial	Multiple micronutrients vs. iron and folic acid	45918	44567	23568
MRCG_ENID	Moore 2012 (23,24)	Trial	Multiple micronutrients vs. protein-energy vs. multiple micronutrients + protein energy vs. iron and folic acid	1195	875	804
LSHTM_Uganda	Webb 2011 (25)	Trial	440 mg albendazole and 40 mg/kg praziquantel vs. 440 mg albendazole and placebo vs. 40 mg/kg praziquantel and placebo vs. placebo and placebo	3389	2515	1278
MAHE_SCFPPP	Ramachandra 2017 (26)	Observational	Examine if foot structural changes influence plantar pressure patterns in pregnancy and postpartum	84	84	68
MAL1	Etheredge 2015 (27)	Trial	Iron vs. placebo	3425	1500	1340
MAL2	Darling 2017 (28)	Trial	Vitamin A vs. Zinc vs. Vitamin A + zinc vs. placebo	16639	2500	1829
MDIG	Roth 2018 (29)	Trial	Vitamin D (4200 IU/week, 16,800 IU/week, or 28,000 IU/week) vs. placebo	1340	1300	1257
MINA_Brazil	Cardoso 2020 (30)	Observational	Assess influence of early environmental exposures and maternal lifestyle on growth and development of children	1753	1551	1301
MISAME_1	Roberfroid 2008 (31)	Trial	Multiple micronutrient supplements United Nations International Preparation (UNIMMAP) + sulphadoxine pyrimethamine vs. UNIMMAP + chloroquine vs. iron and folic acid + sulphadoxine pyrimethamine vs. iron and folic acid + chloroquine	4312	1426	1084
MISAME_2	Huybregts 2009 (32)	Trial	UNIMMAP + sulphadoxine pyrimethamine (2 doses) vs. UNIMMAP + sulphadoxine pyrimethamine (3 doses) vs. lipid-based nutrients + sulphadoxine pyrimethamine (2 doses) vs. lipid-based nutrients sulphadoxine pyrimethamine (3 doses)	1301	1296	1110
MISAME_3	Argaw 2023 (33)	Trial	Fortified BEP supplementation + iron and folic acid vs. iron and folic acid only during pregnancy and lactation	1906	1897	1688
MNIPH_ENISD	Neufeld 2019 (34)	Trial	Nutrivida vs. low-cost micronutrient tablets vs. low-cost micronutrient powders	723	628	412

Supplementary Table 3. Summary of studies included in pooled analysis (additional study details).

Study acronym	Author and date	Study type	Study aim or intervention type	Eligible N[1]	Enrolled N	Analytical N
NNIPS_3	Christian 2003 (35)	Trial	Multiple micronutrients vs. Vitamin A + iron and folic acid vs. Vitamin A + zinc + iron and folic acid vs. Vitamin A + folic acid vs. Vitamin A	4998	4096	2889
NWU_PMPEN	Widen 2019 (36)	Observational	Examine health outcomes associated with food insecurity in pregnant women with and without HIV	-	371	202
NWU_PreNAPS	Widen 2017 (37)	Observational	Assess links between security, psychosocial health, and nutritional status in pregnancy	-	403	235
PNS	Fawzi 2007 (38)	Trial	Multiple micronutrients vs. iron and folic acid	-	8428	7385
PROMOTE_BirthCohort3	Kajubi 2019 (39)	Trial	Sulfadoxine–pyrimethamine vs. dihydroartemisinin–piperaquine	782	782	662
ROSE	Isanaka 2019 (40,41)	Trial	Lipid based nutrient supplements vs. multiple micronutrients vs. iron and folic acid	3370	3332	2136
SAS_WINGS	Taneja 2022 (42)	Trial	Package of delivery of health, nutrition, water, sanitation and hygiene (WaSH), and psychosocial care: preconception only vs. pregnancy only vs. preconception and pregnancy vs. standard care	5525	4921	4455
SBUMS_GDM	Tehrani 2019 (43)	Trial	Gestational diabetes screening methods	35430	35430	23867
SHU_BMIGWG	Soltani 2017 (44)	Observational	Examine association of BMI and gestational weight gain with pregnancy outcomes	-	1013	533
SPAZ_IPTp	Unger 2015 (45)	Trial	Sulphadoxine-pyrimethamine + azithromycin vs. sulphadoxine-pyrimethamine + chloroquine	2793	2775	1908
St_Johns	Unpublished (St-Johns)	Observational	Unspecified	-	-	1557
ARGGWG_CURVES	Calvo 2009 (46)	Observational	Create reference charts for BMI and gestational weight gain in pregnant Argentinian women	-	1439	1067
UCL_LBWSAT	Saville 2018 (47)	Trial	Participatory learning and action group (PLA) vs. PLA + food vs. PLA + cash transfer vs. control (existing government programs)	63308	25092	2504
MIRA_Janakpur	Osrin 2005 (48)	Trial	Multiple micronutrients vs. iron and folic acid	1985[2]	1200	1109
UC_RDNS	Matias 2016 (49)	Trial	Lipid based nutrient supplements vs. iron and folic acid	4022	4011	3344
UHAS_AHPI	Yeboah 2017 (50)	Observational	Examine first trimester serum leptin concentration and body fat percentage and their association with preeclampsia	-	314	282
UMB_CMP	Divala 2018 (51)	Trial	Intermittent sulphadoxine-pyrimethamine vs. intermittent chloroquine vs. weekly chloroquine prophylaxis	1020	900	758
USM_PregCohort	Loy 2014 (52)	Observational	Characterize health in pregnancy (oxidative stress markers, adipokines) and outcomes of pregnancy and in the first year of life	-	153	150

Supplementary Table 3. Summary of studies included in pooled analysis (additional study details).

Study acronym	Author and date	Study type	Study aim or intervention type	Eligible N[1]	Enrolled N	Analytical N
USP_MatStress	Rondo 2003 (53)	Observational	Assess associations between pregnancy psychological stress, distress and low birth weight, prematurity and intrauterine growth retardation	-	1182	901
USP_VIMIP	Dombrowski 2019 (54)	Observational	Examine association of malaria infection in pregnancy with newborn head circumference	-	600	503
UZ_MatNutri	Friis 2004 (55)	Trial	Multiple micronutrients vs. iron and folic acid	-	1669	416
VITAL_MUMTA_PW	Muhammad 2022 (56)	Trial	Antenatal care (ANC) counseling only vs. ANC counseling + balanced energy and protein (BEP) supplement vs. ANC counseling + BEP supplement + 2 doses azithromycin vs. ANC counseling + BEP supplement + nicotinamide and choline	-	1884 (target)	1116
WomenFirst	Hambidge 2019 (57)	Trial	Maternal nutrition supplementation initiated preconception vs. maternal nutrition supplementation initiated at ~11 weeks gestation vs. no supplement	7697[3]	7387[3]	1120
XJU_RuralChina	Zeng 2008 (58)	Trial	Multiple micronutrients vs. iron and folic acid vs. folic acid	6555	5828	4147
XJU_Tibet	Kang 2017 (59)	Trial	Multiple micronutrients vs. iron and folic acid	1717	1149	1014
ZAPPS_3	Castillo 2019 (60)	Observational	Advance understanding of causes of preterm delivery	-	1575	1565
ZamCAT	AMANHI 2021 (61)	Observational	Examine simplified models to assess gestational age in neonates	-	1083	910

[1]Eligible N includes those who refused consent, if reported. Where only invited N was available, this is marked with superscript "[2]".
[2]Invited N reported only (regardless of ineligibility).
3N reported in this column is for 4 study sites, but data were only used for the three sites where gestational age could be ascertained (N~5646)
-: not reported.

Supplementary Table 4. Summary of available outcome data across studies included in pooled analysis: n (%) of observations with outcome (or outcome measured on at least one occasion for repeated postpartum measures).

Study acronym	Analytical sample size	Cesarean delivery	Emergency cesarean delivery	Perineal tears	Postpartum hemorrhage	Prolonged labor	Highest quintile: depressive symptoms	Postpartum weight retained	Postpartum BMI	Postpartum waist circumference	Postpartum systolic blood pressure	Postpartum diastolic blood pressure	Currently any breastfeeding	Currently exclusively breastfeeding
AKU_MMN	1507	1507 (100.0)	-	-	-	-	-	-	-	-	-	-	-	-
AKU_VITD	532	448 (84.2)	-	-	-	-	-	413 (77.6)	413 (77.6)	-	437 (82.1)	417 (78.4)	-	-
Calcium_India	10472	10463 (99.9)	-	10042 (95.9)	10035 (95.8)	-	7996 (76.4)	7987 (76.3)	7987 (76.3)	-	7989 (76.3)	7989 (76.3)	9989 (95.4)	-
Calcium_Tanzania	9954	9887 (99.3)	-	-	-	-	-	-	-	-	-	-	-	-
EU_MMN	353	352 (99.7)	-	-	-	-	-	-	-	-	-	-	-	-
FU_GWG	146	127 (87.0)	-	-	-	-	-	-	-	-	-	-	-	-
FU_LEPTINGWG	232	216 (93.1)	-	-	-	-	-	196 (84.5)	196 (84.5)	-	166 (71.6)	166 (71.6)	-	-
GWU_MINT	698	695 (99.6)	693 (99.3)	-	-	655 (93.8)	-	490 (70.2)	490 (70.2)	-	493 (70.6)	494 (70.8)	-	-
ICDDR_MINIMat	3482	3269 (93.9)	-	3089 (88.7)	3229 (92.7)	-	-	-	-	-	-	-	-	-
ILINS_DYAD_G	1115	1112 (99.7)	-	-	-	-	953 (85.5)	947 (84.9)	947 (84.9)	-	-	-	-	-
ILINS_DYAD_M	1321	1205 (91.2)	1195 (90.5)	1186 (89.8)	1187 (89.9)	1175 (88.9)	1113 (84.3)	1212 (91.7)	1212 (91.7)	-	375 (28.4)	373 (28.2)	300 (22.7)	300 (22.7)
IMIP_BRAMAG	863	718 (83.2)	-	-	-	-	-	-	-	-	-	-	-	-
IMIP_GestDM	506	376 (74.3)	-	-	-	-	-	-	-	-	-	-	-	-
INPer_CAR	398	395 (99.2)	-	-	-	-	-	-	-	-	-	-	-	-
INPer_FICA	164	164 (100.0)	-	-	-	-	-	160 (97.6)	160 (97.6)	-	-	-	164 (100.0)	164 (100.0)
INPer_NeuroObesity	274	254 (92.7)	-	-	-	-	-	-	-	183 (66.8)	-	-	183 (66.8)	210 (76.6)
INPer_Poli	160	137 (85.6)	-	-	-	-	-	84 (52.5)	84 (52.5)	-	-	-	-	-
INPer_REDES	327	327 (100.0)	-	-	-	-	-	-	-	-	-	-	-	-
INSPM_ELEMENT	732	729 (99.6)	520 (71.0)	-	-	-	-	704 (96.2)	704 (96.2)	-	705 (96.3)	705 (96.3)	705 (96.3)	-

Supplementary Table 4. Summary of available outcome data across studies included in pooled analysis: n (%) of observations with outcome (or outcome measured on at least one occasion for repeated postpartum measures).

Study acronym	Analytical sample size	Cesarean delivery	Emergency cesarean delivery	Perineal tears	Postpartum hemorrhage	Prolonged labor	Highest quintile: depressive symptoms	Postpartum weight retained	Postpartum BMI	Postpartum waist circumference	Postpartum systolic blood pressure	Postpartum diastolic blood pressure	Currently any breastfeeding	Currently exclusively breastfeeding
INSPM_PROGRESS	926	926 (100.0)	606 (65.4)	-	-	-	731 (78.9)	792 (85.5)	792 (85.5)	-	792 (85.5)	792 (85.5)	-	630 (68.0)
IRD_RECIPAL	253	253 (100.0)	246 (97.2)	-	-	-	-	-	-	-	-	-	-	-
JHU_NOMS	19411	-	-	-	-	17416 (89.7)	-	294 (1.5)	294 (1.5)	-	298 (1.5)	298 (1.5)	-	-
JiVitA3	23568	-	-	21988 (93.3)	22084 (93.7)	22875 (97.1)	7385 (31.3)	23392 (99.3)	23392 (99.3)	-	-	-	22757 (96.6)	22757 (96.6)
MRCG_ENID	804	367 (45.6)	-	-	-	-	-	654 (81.3)	654 (81.3)	-	-	-	-	-
LSHTM_Uganda	1278	1226 (95.9)	-	-	-	-	-	1169 (91.5)	1169 (91.5)	-	1190 (93.1)	1190 (93.1)	-	-
MAHE_SCFPPP	68	68 (100.0)	-	-	-	-	-	68 (100.0)	68 (100.0)	-	-	-	-	-
MAL1	1340	1340 (100.0)	1334 (99.6)	1243 (92.8)	1209 (90.2)	-	-	631 (47.1)	631 (47.1)	-	634 (47.3)	634 (47.3)	951 (71.0)	951 (71.0)
MAL2	1829	1792 (98.0)	1775 (97.0)	1274 (69.7)	-	-	-	1173 (64.1)	1173 (64.1)	-	1252 (68.5)	1252 (68.5)	1657 (90.6)	1657 (90.6)
MDIG	1257	1228 (97.7)	-	-	-	-	-	1125 (89.5)	1125 (89.5)	-	-	-	1176 (93.6)	1176 (93.6)
MINA_Brazil	1301	1301 (100.0)	-	-	1301 (100.0)	-	-	-	-	-	-	-	-	-
MISAME_1	1084	1065 (98.2)	-	-	-	-	-	-	-	-	-	-	-	-
MISAME_2	1110	1087 (97.9)	-	-	-	-	-	-	-	-	-	-	-	-
MISAME_3	1688	1649 (97.7)	-	-	-	-	-	1558 (92.3)	1558 (92.3)	-	-	-	-	-
MNIPH_ENISD	412	-	-	-	-	-	-	410 (99.5)	410 (99.5)	409 (99.3)	-	-	410 (99.5)	-
NNIPS_3	2889	-	-	-	-	2847 (98.5)	-	2759 (95.5)	2759 (95.5)	-	-	-	2609 (90.3)	-
NWU_PMPEN	202	184 (91.1)	-	-	-	-	-	199 (98.5)	199 (98.5)	-	140 (69.3)	140 (69.3)	-	-
NWU_PreNAPS	235	235 (100.0)	229 (97.4)	-	-	227 (96.6)	221 (94.0)	233 (99.1)	233 (99.1)	233 (99.1)	-	-	222 (94.5)	-
PNS	7385	7227 (97.9)	7153 (96.9)	6913 (93.6)	7044 (95.4)	7020 (95.1)	-	6798 (92.1)	6798 (92.1)	-	6580 (89.1)	6580 (89.1)	-	-

Supplementary Table 4. Summary of available outcome data across studies included in pooled analysis: n (%) of observations with outcome (or outcome measured on at least one occasion for repeated postpartum measures).

Study acronym	Analytical sample size	Cesarean delivery	Emergency cesarean delivery	Perineal tears	Postpartum hemorrhage	Prolonged labor	Highest quintile: depressive symptoms	Postpartum weight retained	Postpartum BMI	Postpartum waist circumference	Postpartum systolic blood pressure	Postpartum diastolic blood pressure	Currently any breastfeeding	Currently exclusively breastfeeding
PROMOTE_Birth Cohort3	662	653 (98.6)	-	-	-	-	-	654 (98.8)	654 (98.8)	-	655 (98.9)	655 (98.9)	-	-
ROSE	2136	2136 (100.0)	-	-	-	-	-	-	-	-	-	-	1927 (90.2)	1927 (90.2)
SAS_WINGS	4455	-	-	-	-	-	-	4112 (92.3)	4112 (92.3)	-	-	-	-	-
SBUMS_GDM	23867	23867 (100.0)	-	-	-	-	-	-	-	-	-	-	-	-
SHU_BMIGWG	533	451 (84.6)	-	401 (75.2)	416 (78.0)	-	-	-	-	-	-	-	-	-
SPAZ_IPTp	1908	1904 (99.8)	-	-	-	-	-	655 (34.3)	655 (34.3)	-	85 (4.5)	85 (4.5)	-	-
St_Johns	1557	1557 (100.0)	-	-	-	-	-	-	-	-	-	-	-	-
ARG_GWGCURVES	1067	1066 (99.9)	-	-	-	-	-	-	-	-	-	-	-	-
UCL_LBWSAT	2504	2478 (99.0)	2478 (99.0)	-	514 (20.5)	-	-	1994 (79.6)	1994 (79.6)	-	-	-	-	-
MIRA_Janakpur	1109	1103 (99.5)	-	-	-	-	-	-	-	-	-	-	-	-
UC_RDNS	3344	3121 (93.3)	-	-	-	-	-	-	-	-	-	-	-	-
UHAS_AHPI	282	263 (93.3)	-	-	-	-	-	-	-	-	-	-	-	-
UMB_CMP	758	758 (100.0)	-	-	-	-	-	667 (88.0)	667 (88.0)	-	690 (91.0)	690 (91.0)	-	-
USM_PregCohort	150	150 (100.0)	-	-	-	-	-	-	-	-	-	-	144 (96.0)	143 (95.3)
USP_MatStress	901	901 (100.0)	-	-	-	-	-	-	-	-	-	-	-	-
USP_VIMIP	503	478 (95.0)	-	-	-	-	-	-	-	-	-	-	-	-
UZ_MatNutri	416	413 (99.3)	413 (99.3)	-	-	-	-	261 (62.7)	261 (62.7)	-	-	-	-	-
VITAL_MUMTA_PW	1116	1116 (100.0)	991 (88.8)	-	-	-	-	607 (54.4)	607 (54.4)	-	-	-	-	-
WomenFirst	1120	1120 (100.0)	-	-	-	-	-	-	-	-	-	-	-	-

Supplementary Table 4. Summary of available outcome data across studies included in pooled analysis: n (%) of observations with outcome (or outcome measured on at least one occasion for repeated postpartum measures).

Study acronym	Analytical sample size	Cesarean delivery	Emergency cesarean delivery	Perineal tears	Postpartum hemorrhage	Prolonged labor	Highest quintile: depressive symptoms	Postpartum weight retained	Postpartum BMI	Postpartum waist circumference	Postpartum systolic blood pressure	Postpartum diastolic blood pressure	Currently any breastfeeding	Currently exclusively breastfeeding
XJU_RuralChina	4147	4095 (98.7)	3975 (95.9)	-	-	-	-	-	-	-	-	-	-	-
XJU_Tibet	1014	998 (98.4)	969 (95.6)	-	-	-	-	-	-	-	-	-	-	-
ZAPPS_3	1565	1188 (75.9)	1118 (71.4)	-	1195 (76.4)	1154 (73.7)	1018 (65.0)	-	-	-	-	-	-	-
ZamCAT	910	784 (86.2)	780 (85.7)	-	764 (84.0)	781 (85.8)	-	816 (89.7)	816 (89.7)	-	821 (90.2)	821 (90.2)	-	-

-: outcome not available in this study.
For each outcome, studies which were not considered for pooling due to extensive missingness on outcome values (defined as n<50 observations and <10% of total sample available) are also denoted by "-". Where any such studies were excluded, the number of such studies for each outcome was: perineal tears (1 study), postpartum hemorrhage (2 studies), prolonged labor (2 studies), postpartum weight retained (5 studies), postpartum BMI (5 studies), postpartum systolic blood pressure (5 studies), postpartum diastolic blood pressure (5 studies), currently any breastfeeding (3 studies).

Supplementary Table 5. Risk of Bias assessment for included studies.

	Domain			
**Study acronym**	**Study participation**	**Study attrition**	**Measurement of exposure (maternal weight)**	**Measurement of maternal outcomes**
AKU_MMN	Low	Low	Low	Low
AKU_VITD	Moderate	Moderate	Low	Low
Calcium_India	Low	Low	Low	Low
Calcium_Tanzania	Low	Low	Low	Low
EU_MMN	Low	Low	Low	Low
FU_GWG	Low	Low	Low	Low
FU_LEPTINGWG	Low	Moderate	Low	Low
GWU_MINT	Low	Moderate	Low	Low
ICDDR_MINIMat	Low	Low	Low	Low
ILINS_DYAD_G	Low	Low	Low	Low
ILINS_DYAD_M	Low	Moderate	Low	Low
IMIP_BRAMAG	Moderate	Low	Low	Low
IMIP_GestDM	Moderate	Moderate	Low	Low
INPer_CAR*	-	-	Low	Low
INPer_FICA	Low	Moderate	Low	Low
INPer_NeuroObesity*	-	-	Low	Low
INPer_Poli*	-	-	Low	Low
INPer_REDES	Low	Low	Low	Low
INSPM_ELEMENT	Low	Moderate	Low	Low
INSPM_PROGRESS	Low	Moderate	Low	Low
IRD_RECIPAL	Low	Low	Low	Low
JHU_NOMS	Low	High	Low	Low
JiVitA3	Low	Moderate	Low	Low
MRCG_ENID	Low	Moderate	Low	Low
LSHTM_Uganda	Low	Moderate	Low	Low
MAHE_SCFPPP	Low	Moderate	Low	Low
MAL1	Low	Moderate	Low	Low
MAL2	Low	Moderate	Low	Low
MDIG	Low	Moderate	Low	Low
MINA_Brazil	Low	Low	Low	Low
MISAME_1	Low	Low	Low	Low
MISAME_2	Low	Moderate	Low	Low
MISAME_3	Low	Low	Low	Low
MNIPH_ENISD	Low	Low	Low	Low
NNIPS_3	Low	Moderate	Low	Low
NWU_PMPEN	Low	Low	Low	Moderate
NWU_PreNAPS	Low	Low	Low	Low
PNS	Low	Low	Low	Low
PROMOTE_BirthCohort3	Low	Low	Low	Low
ROSE	Moderate	Moderate	Low	Low
SAS_WINGS	Low	Low	Low	Low
SBUMS_GDM	Moderate	Low	Low	Low

Supplementary Table 5. Risk of Bias assessment for included studies.

	Domain			
**Study acronym**	**Study participation**	**Study attrition**	**Measurement of exposure (maternal weight)**	**Measurement of maternal outcomes**
SHU_BMIGWG	Low	Moderate	Low	Low
SPAZ_IPTp	Moderate	Moderate	Low	Low
St_Johns*	-	-	Low	Low
ARG_GWGCURVES	Low	Low	Low	Low
UCL_LBWSAT	Low	High	Low	Low
MIRA_Janakpur	Low	Moderate	Low	Low
UC_RDNS	Low	Moderate	Low	Low
UHAS_AHPI	High	Moderate	Low	Moderate
UMB_CMP	Low	Moderate	Low	Low
USM_PregCohort	Moderate	Moderate	Low	Low
USP_MatStress	Moderate	Low	Low	Low
USP_VIMIP	Low	Low	Low	Low
UZ_MatNutri	Low	Low	Low	Low
VITAL_MUMTA_PW	Low	Moderate	Low	Low
WomenFirst	Low	Low	Low	Low
XJU_RuralChina	Low	Moderate	Low	Low
XJU_Tibet	Moderate	Low	Low	Low
ZAPPS_3	Low	Moderate	Low	Low
ZamCAT	Low	Low	Low	Low

Risk of Bias for noted domains assessed using QUIPS Risk of Bias Assessment Instrument for Prognostic Factor Studies.
*Unpublished study.

Supplementary Table 6. Associations between sub-optimal gestational weight gain (GWG) versus adequate GWG and maternal perinatal and postpartum outcomes - estimates with prediction intervals.

	Studies	N[1]	n[2]	RR or MD (95%PI)	τ^2
**Severely inadequate GWG**					
**Perinatal outcomes (RR)**					
Cesarean delivery[3]	55	53,135	10,270	0.82 (0.67-1.01)	0.010
Emergency cesarean delivery	15	13,719	1,026	0.73 (0.35-1.54)	0.103
Perineal tears	8	29,962	3,290	0.95 (0.88-1.03)	0.000
Postpartum hemorrhage	**11**	**31,389**	**7,412**	**0.91 (0.86-0.96)**	**0.000**
Prolonged labor	9	37,867	5,928	0.82 (0.45-1.48)	0.050
**Psychosocial measures (RR)**					
Highest quintile of depressive symptom score	7	12,416	1,343	0.99 (0.94-1.05)	0.000
**Postpartum weight and related measures (MD)**					
Postpartum weight retained, kg	32	99,021		-2.82 (-5.96-0.32)	2.257
BMI, kg/m^2	32	99,021		-1.16 (-2.35-0.03)	0.326
Waist circumference, cm	3	870		-3.05 (-25.59-19.49)	1.744
Systolic blood pressure, mmHg	17	30,449		-1.15 (-3.92-1.62)	1.515
Diastolic blood pressure, mmHg	17	30,421		-0.58 (-1.21-0.05)	0.056
**Breastfeeding (RR)**					
Currently any breastfeeding	14	97,586	91,046	1.00 (0.99-1.01)	0.000
Currently exclusively breastfeeding	10	89,484	36,587	0.99 (0.91-1.07)	0.001
**Moderately inadequate GWG**					
**Perinatal outcomes (RR)**					
Cesarean delivery[2]	54	48,047	10,638	0.88 (0.76-1.01)	0.004
Emergency cesarean delivery	15	12,249	978	0.82 (0.67-1.01)	0.004
Perineal tears	8	21,069	2,592	0.90 (0.66-1.22)	0.012
Postpartum hemorrhage	10	22,081	3,757	0.97 (0.91-1.04)	0.000
Prolonged labor	9	23,219	3,648	1.01 (0.93-1.09)	0.000
**Psychosocial measures (RR)**					
Highest quintile of depressive symptom score	7	10,405	981	0.98 (0.93-1.03)	0.000
**Postpartum weight and related measures (MD)**					
Postpartum weight retained, kg	32	69,595		-1.13 (-2.43-0.18)	0.391
BMI, kg/m^2	32	69,595		-0.81 (-1.73-0.11)	0.193
Waist circumference, cm	3	734		-1.98 (-22.26-18.29)	1.722
Systolic blood pressure, mmHg	17	30,481		-0.80 (-2.16-0.57)	0.322
Diastolic blood pressure, mmHg	17	30,453		-0.47 (-1.55-0.61)	0.182
**Breastfeeding (RR)**					
Currently any breastfeeding	14	61,693	57,987	1.00 (0.99-1.01)	0.000
Currently exclusively breastfeeding	10	53,252	24,074	0.99 (0.93-1.05)	0.000
**Excessive GWG**					
**Perinatal outcomes (RR)**					
Cesarean delivery[2]	50	55,379	17,539	**1.10 (1.06-1.13)**	0.000
Emergency cesarean delivery	14	10,190	1,202	**1.22 (1.03-1.43)**	0.000
Perineal tears	8	16,069	2,175	1.06 (0.82-1.37)	0.008
Postpartum hemorrhage	11	17,438	1,636	0.99 (0.15-6.60)	0.608

Supplementary Table 6. Associations between sub-optimal gestational weight gain (GWG) versus adequate GWG and maternal perinatal and postpartum outcomes - estimates with prediction intervals.

	Studies	N[1]	n[2]	RR or MD (95%PI)	τ^2
Prolonged labor	9	13,092	1,935	1.03 (0.86-1.23)	0.000
**Psychosocial measures (RR)**					
Highest quintile of depressive symptom score	7	10,639	1,040	1.04 (0.98-1.12)	0.000
**Postpartum weight and related measures (MD)**					
Postpartum weight retained, kg	32	50,410		2.00 (-0.40-4.40)	1.317
BMI, kg/m^2	32	50,410		0.96 (-0.18-2.11)	0.299
Waist circumference, cm	3	948		0.85 (-3.32-5.02)	0.000
Systolic blood pressure, mmHg	17	28,801		0.36 (-2.73-3.46)	1.740
Diastolic blood pressure, mmHg	17	28,773		1.12 (-4.88-7.13)	7.368
**Breastfeeding (RR)**					
Currently any breastfeeding	14	44,064	40,874	1.00 (1.00-1.00)	0.000
Currently exclusively breastfeeding	10	34,792	16,512	0.99 (0.93-1.05)	0.000

BMI: Body mass index. RR: Risk ratio. MD: mean difference. 95%PI: 95% prediction interval.
[1]N= number of participants or number of observations for repeated measures (postpartum depressive symptoms, weight and related measures, and breastfeeding), for each comparison.
[2]n = number of participants with event, where RR is reported.
[3]Cesarean delivery includes both emergency and scheduled cesarean delivery.
Gestational weight gain (GWG) adequacy was defined as ratio of observed GWG versus GWG recommended in line with Institute of Medicine 2009 guidelines. Severely inadequate GWG was defined as GWG adequacy ratio <70%, moderately inadequate as GWG adequacy ratio 70% to <90%, adequate as GWG adequacy ratio 90% to 125%, and excessive as GWG adequacy ratio > 125%.
Estimates pooled using random-effects meta-analysis (REML method), with Hartung-Knapp adjustment for standard errors.

Supplementary Table 7. Pooled effect estimates for interaction between pre-pregnancy BMI and gestational weight gain adequacy.

Outcome	Studies	RRR or DMD (95%CI)	τ^2
**Perinatal outcomes (RRR)**			
Cesarean delivery[1]	**56**	**0.98 (0.97-0.99)**	**0.000**
Emergency cesarean delivery	16	0.97 (0.92-1.04)	0.001
Perineal tears	8	0.98 (0.94-1.03)	0.000
Postpartum hemorrhage	11	0.99 (0.93-1.05)	0.000
Prolonged labor	9	0.99 (0.96-1.03)	0.000
**Psychosocial measures (RRR)**			
Highest quintile of depressive symptom score	7	1.01 (0.99-1.03)	0.000
**Postpartum weight and related measures (DMD)**			
Postpartum weight retained, kg	**32**	**-0.10 (-0.20-0.00)**	**0.035**
BMI, kg/m^2	**32**	**-0.08 (-0.13--0.03)**	**0.006**
Waist circumference, cm	3	-0.28 (-3.95-3.39)	1.889
Systolic blood pressure, mmHg	**17**	**-0.17 (-0.29--0.05)**	**0.000**
Diastolic blood pressure, mmHg	17 s	0.03 (-0.10-0.16)	0.011
**Breastfeeding (RRR)**			
Currently any breastfeeding	14	1.00 (1.00-1.00)	0.000
Currently exclusively breastfeeding	10	1.00 (1.00-1.00)	0.000

BMI: Body mass index. RRR: Ratio of risk ratio. DMD: difference in mean difference. 95%CI: 95% confidence interval.

For binary outcomes, RRR represents the ratio of (risk ratio of outcome per 1% increase in GWG adequacy ratio) for each 1 kg/m^2 increase in BMI. For continuous outcomes, the DMD represents the difference in (mean difference of outcome per 1% increase in GWG adequacy ratio) for each 1 kg/m^2 increase in BMI.

Interaction estimated using variables of category-specific medians for BMI and GWG, treated as continuous variables.

[1]Cesarean delivery includes both emergency and scheduled cesarean delivery.

Estimates pooled using random-effects meta-analysis (REML method), with Hartung-Knapp adjustment for standard errors.

Supplementary Table 8. Associations between sub-optimal gestational weight gain (versus adequate gestational weight gain) and key maternal perinatal and postpartum outcomes, stratified by maternal pre-pregnancy BMI.

	Severely inadequate			Moderately inadequate			Excessive		
	Studies	RR or MD (95%CI)	τ^2	Studies	RR or MD (95%CI)	τ^2	Studies	RR or MD (95%CI)	τ^2
**Cesarean delivery (RR)**									
**Pre-pregnancy[2] underweight**	24	0.77 (0.66-0.90)	0.001	19	0.87 (0.64-1.19)*	0.234	19	1.00 (0.83-1.20)	0.000
**Pre-pregnancy[2] normal BMI**	53	0.83 (0.79-0.87)	0.000	54	0.86 (0.82-0.91)	0.006	46	1.11 (1.07-1.16)	0.000
**Pre-pregnancy[2] overweight or obese**	41	0.91 (0.80-1.04)	0.042	40	0.98 (0.91-1.06)	0.000	42	1.06 (0.98-1.15)	0.006
**Postpartum weight retained, kg (MD)**									
**Pre-pregnancy[2] underweight**	26	-2.37 (-3.44--1.31)	4.379	22	-1.12 (-1.83--0.41)	2.215	19	2.68 (1.38-3.98)	5.844
**Pre-pregnancy[2] normal BMI**	32	-2.95 (-3.53--2.37)	1.909	32	-1.21 (-1.50--0.92)	0.424	31	2.18 (1.64-2.72)	1.404
**Pre-pregnancy[2] overweight or obese**	27	-2.08 (-3.02--1.14)	3.164	23	-0.92 (-1.62--0.22)	1.386	24	2.16 (1.70-2.63)	0.538
**BMI, kg/m^2 (MD)**									
**Pre-pregnancy[2] underweight**	26	-0.98 (-1.54--0.42)	1.194	22	-0.47 (-0.73--0.21)	0.264	19	0.80 (0.27-1.32)	0.839
**Pre-pregnancy[2] normal BMI**	32	-1.29 (-1.60--0.97)	0.631	32	-0.80 (-0.98--0.62)	0.178	31	0.80 (0.53-1.07)	0.356
**Pre-pregnancy[2] overweight or obese**	27	-0.55 (-0.89--0.21)	0.274	23	-0.37 (-0.85-0.10)	0.658	23	1.47 (1.10-1.84)	0.253
**Systolic blood pressure, mmHg (MD)**									
**Pre-pregnancy[1] underweight**	15	-1.59 (-4.90-1.73)	31.751	13	0.22 (-2.73-3.16)	20.548	10	3.65 (-2.31-9.60)	26.780
**Pre-pregnancy[1] normal BMI**	17	-1.21 (-2.39--0.03)	1.372	17	-0.57 (-1.29-0.14)	0.520	18	-0.15 (-1.60-1.31)	3.081
**Pre-pregnancy[1] overweight or obese**	14	-2.19 (-5.14-0.76)	11.156	14	-2.75 (-4.81--0.70)	3.654	18	0.55 (-0.11-1.20)	0.000

BMI: Body mass index. RR: Risk ratio. MD: mean difference. 95%CI: 95% confidence interval.
[1]Cesarean delivery includes both emergency and scheduled cesarean delivery.
[2]Pre-pregnancy BMI, or first trimester BMI used as a proxy where pre-pregnancy BMI was not available.
Gestational weight gain (GWG) adequacy was defined as ratio of observed GWG versus GWG recommended in line with Institute of Medicine 2009 guidelines. Severely inadequate GWG was defined as GWG adequacy ratio <70%, moderately inadequate as GWG adequacy ratio 70% to <90%, adequate as GWG adequacy ratio 90% to 125%, and excessive as GWG adequacy ratio > 125%.
Estimates pooled using random-effects meta-analysis (REML method), with Hartung-Knapp adjustment for standard errors.
*Sidik-Jonkman random effects meta-analysis was used in lieu of REML method for meta-analysis as tau could not be estimated.

Supplementary Table 9. Associations between sub-optimal gestational weight gain (versus adequate gestational weight gain) defined using INTERGROWTH-21st standards and maternal perinatal and postpartum outcomes (among women with normal pre-pregnancy or first trimester BMI only).

	Severely inadequate			Moderately inadequate			Excessive		
**Outcome**	**Studies**	**RR or MD (95%CI)**	**τ^2**	**Studies**	**RR or MD (95%CI)**	**τ^2**	**Studies**	**RR or MD (95% CI)**	**τ^2**
**Perinatal outcomes (RR)**									
Cesarean delivery[1]	51	0.79 (0.74, 0.84)	0.012	51	0.88 (0.85, 0.92)	0.000	44	1.09 (1.02, 1.17)	0.009
Emergency cesarean delivery	14	0.76 (0.65, 0.89)	0.010	14	0.82 (0.71, 0.94)	0.000	12	1.18 (0.90, 1.53)	0.079
Perineal tears	7	0.95 (0.86, 1.05)	0.000	7	0.92 (0.84, 1.00)	0.000	7	0.96 (0.76, 1.21)	0.000
Postpartum hemorrhage	9	0.98 (0.72, 1.34)	0.057	8	0.96 (0.89, 1.04)	0.000	7	1.42 (0.78, 2.58)	0.083
Prolonged labor	9	0.98 (0.91, 1.06)	0.000	9	1.01 (0.93, 1.09)	0.000	5	0.98 (0.78, 1.23)	0.000
**Psychosocial measures (RR)**									
Highest quintile of depressive symptom score	7	0.98 (0.93, 1.04)	0.000	7	0.99 (0.95, 1.04)	0.000	7	1.01 (0.81, 1.26)	0.073
**Postpartum weight and related measures (MD)**									
Postpartum weight retained, kg	32	-3.02 (-3.63, -2.41)	2.643	31	-1.20 (-1.52, -0.89)	0.502	30	1.89 (1.22, 2.56)	2.625
BMI, kg/m^2	32	-1.26 (-1.56, -0.97)	0.608	31	-0.59 (-0.72, -0.46)	0.061	30	0.85 (0.52, 1.18)	0.552
Waist circumference, cm	3	-3.40 (-5.91, -0.89)	2.307	3	-0.71 (-2.10, 0.67)	0.000	3	4.77 (-3.38, 12.92)	46.738
Systolic blood pressure, mmHg	17	-0.80 (-1.36, -0.24)	0.290	17	-0.58 (-0.89, -0.27)	0.000	17	-0.21 (-1.61, 1.19)	4.049
Diastolic blood pressure, mmHg	17	-0.65 (-0.92, -0.38)	0.000	17	-0.16 (-0.41, 0.09)	0.000	17	0.58 (-0.23, 1.39)	0.742
**Breastfeeding (RR)**									
Currently any breastfeeding	14	1.00 (1.00, 1.01)	0.000	14	1.00 (1.00, 1.00)	0.000	13	1.00 (1.00, 1.00)	0.000
Currently exclusively breastfeeding	10	1.01 (0.99, 1.03)	0.000	10	1.01 (0.99, 1.02)	0.000	9	1.02 (1.00, 1.04)	0.000

BMI: Body mass index. RR: Risk ratio. MD: mean difference. 95%CI: 95% confidence interval.
[1]Cesarean delivery includes both emergency and scheduled cesarean delivery.
Gestational weight gain (GWG) adequacy was defined as ratio of observed GWG versus GWG recommended in line with Institute of Medicine 2009 guidelines. Severely inadequate GWG was defined as GWG adequacy ratio <70%, moderately inadequate as GWG adequacy ratio 70% to <90%, adequate as GWG adequacy ratio 90% to 125%, and excessive as GWG adequacy ratio > 125%.
Estimates pooled using random-effects meta-analysis (DerSimonian-Laird method).

Supplementary Table 10. Distribution of participants across GWG categories when including versus excluding women with imputed first trimester weight values.

	N	%
**Including women with imputed first trimester weight values**		
Severely Inadequate	53,860	34.5
Moderately Inadequate	33,773	21.6
Adequate	37,146	23.8
Excessive	31,521	20.2
**Total**	**156,300**	
**Excluding women with imputed first trimester weight values**		
Severely Inadequate	39,638	37.5
Moderately Inadequate	19,887	18.8
Adequate	24,283	23.0
Excessive	21,869	20.7
**Total**	**105,677**	

Supplementary Table 11. Associations between sub-optimal gestational weight gain (versus adequate gestational weight gain) and maternal perinatal and postpartum outcomes - analyses restricted to participants with GWG adequacy based on measured (i.e. non-imputed) first trimester weight values.

	Severely inadequate			Moderately inadequate			Excessive		
	Studies	RR or MD (95%CI)	τ^2	Studies	RR or MD (95%CI)	τ^2	Studies	RR or MD (95%CI)	τ^2
Cesarean delivery[1]	46	0.85 (0.78, 0.92)	0.020	43	0.93 (0.89, 0.97)	0.000	42	1.19 (1.13, 1.26)	0.005
Emergency cesarean delivery	11	0.66 (0.48, 0.91)	0.104	12	0.81 (0.64, 1.03)	0.000	10	1.11 (0.83, 1.49)	0.077
Perineal tears	7	0.96 (0.86, 1.07)	0.001	7	0.95 (0.77, 1.18)	0.034	7	1.00 (0.87, 1.15)	0.000
Postpartum hemorrhage	6	0.95 (0.80, 1.12)	0.010	6	0.93 (0.67, 1.28)	0.059	6	1.04 (0.90, 1.20)	0.001
Prolonged labor	7	0.90 (0.77, 1.04)	0.014	6	0.98 (0.87, 1.10)	0.006	5	0.94 (0.72, 1.23)	0.038
Highest quintile of depressive symptom score	6	1.00 (0.92, 1.08)	0.003	6	0.98 (0.89, 1.08)	0.005	6	1.09 (0.95, 1.25)	0.014
Postpartum weight retained, kg	22	-3.52 (-4.32, -2.73)	2.779	22	-1.15 (-1.57, -0.74)	0.454	22	1.66 (1.16, 2.15)	0.659
BMI, kg/m^2	22	-1.03 (-1.48, -0.59)	0.696	22	-0.89 (-1.26, -0.53)	0.375	23	2.06 (0.59, 3.53)	12.284
Waist circumference, cm	3	1.05 (-2.57, 4.67)	0.000	3	4.37 (-1.08, 9.81)	12.054	3	4.58 (1.71, 7.44)	0.000
Systolic blood pressure, mmHg	11	-0.57 (-1.89, 0.74)	2.270	11	-0.40 (-0.98, 0.18)	0.000	12	0.49 (-2.90, 3.88)	29.642
Diastolic blood pressure, mmHg	11	-0.80 (-1.78, 0.17)	1.091	11	-0.68 (-1.56, 0.20)	0.656	12	0.81 (-2.36, 3.99)	26.200
Currently any breastfeeding	13	1.00 (1.00, 1.00)	0.000	12	1.00 (1.00, 1.00)	0.000	13	1.00 (1.00, 1.00)	0.000
Currently exclusively breastfeeding	7	1.00 (0.95, 1.05)	0.001	6	1.00 (0.97, 1.03)	0.000	7	1.00 (0.97, 1.05)	0.001

BMI: Body mass index. RR: Risk ratio. MD: mean difference. 95%CI: 95% confidence interval.
[1]Cesarean delivery includes both emergency and scheduled cesarean delivery.
Gestational weight gain (GWG) adequacy was defined as ratio of observed GWG versus GWG recommended in line with Institute of Medicine 2009 guidelines. Severely inadequate GWG was defined as GWG adequacy ratio <70%, moderately inadequate as GWG adequacy ratio 70% to <90%, adequate as GWG adequacy ratio 90% to 125%, and excessive as GWG adequacy ratio > 125%.
Estimates pooled using random-effects meta-analysis (DerSimonian-Laird method).

Supplementary Table 12. Associations between sub-optimal gestational weight gain (versus adequate gestational weight gain) and maternal perinatal and postpartum outcomes - analyses done using complete case analysis to handle missing values.

	Severely inadequate				Moderately inadequate				Excessive			
	**Studies**	**N**	**RR or MD (95%CI)**	**τ^2**	**Studies**	**N**	**RR or MD (95%CI)**	**τ^2**	**Studies**	**N**	**RR or MD (95%CI)**	**τ^2**
**Perinatal outcomes (RR)**												
**Cesarean delivery**	**49**	**84,411**	**0.85 (0.80-0.91)**	**0.004**	**49**	**84,534**	**0.89 (0.84-0.94)**	**0.005**	**47**	**82,892**	**1.08 (1.04-1.12)**	**0.001**
**Emergency cesarean delivery**	12	14,538	0.75 (0.52-1.08)	0.161	12	14,699	0.89 (0.72-1.10)	0.022	12	14,538	1.16 (0.94-1.44)	0.000
**Perineal tears**	8	39,226	0.97 (0.92-1.02)	0.000	8	39,226	0.90 (0.80-1.00)	0.008	8	39,226	1.00 (0.85-1.18)	0.018
**Postpartum hemorrhage**	11	41,393	1.03 (0.83-1.26)	0.020	9	40,133	1.00 (0.91-1.09)	0.003	11	41,393	0.88 (0.46-1.69)	0.486
**Prolonged labor**	9	47,564	0.72 (0.51-1.01)	0.124	9	47,564	1.01 (0.93-1.10)	0.000	9	47,564	0.98 (0.83-1.16)	0.004
**Psychosocial measures (RR)**												
**Highest quintile of depressive symptom score**	7	11,647	1.00 (0.93-1.07)	0.000	7	11,647	1.00 (0.89-1.11)	0.003	6	11,479	1.08 (0.78-1.51)	0.069
**Postpartum weight and related measures (MD)**												
**Postpartum weight retained, kg**	**28**	**132,964**	**-2.89 (-3.66--2.11)**	**2.899**	**28**	**132,964**	**-1.16 (-1.49--0.83)**	**0.482**	**28**	**132,964**	**2.24 (1.68-2.79)**	**1.177**
**BMI, kg/m2**	**28**	**132,964**	**-1.16 (-1.44--0.88)**	**0.366**	**28**	**132,964**	**-0.75 (-0.96--0.55)**	**0.193**	**28**	**132,964**	**1.08 (0.32-1.84)**	**3.061**
**Waist circumference, cm**	**3**	**1,226**	**-3.10 (-5.86--0.34)**	**0.148**	3	1,226	-1.85 (-4.70-1.01)	0.870	3	1,226	0.87 (-0.93-2.66)	0.000
**Systolic blood pressure, mmHg**	**16**	**51,787**	**-1.15 (-2.08--0.22)**	**1.252**	16	51,787	-0.75 (-1.53-0.04)	0.378	16	51,787	0.31 (-0.85-1.47)	0.538
**Diastolic blood pressure, mmHg**	**16**	**51,724**	**-0.73 (-1.01--0.44)**	**0.000**	16	51,724	-0.29 (-1.13-0.55)	0.469	16	51,724	0.36 (-0.67-1.38)	0.943
**Breastfeeding (RR)**												
**Currently any breastfeeding**	6	41,120	1.00 (1.00-1.00)	0.000	6	41,120	1.00 (1.00-1.00)	0.000	6	41,120	1.00 (1.00-1.00)	0.000
**Currently exclusively breastfeeding**	5	40,247	1.00 (0.93-1.08)	0.001	5	40,247	0.99 (0.89-1.11)	0.002	5	40,247	0.99 (0.88-1.11)	0.003

BMI: Body mass index. RR: Risk ratio. MD: mean difference. 95%CI: 95% confidence interval.
N= number of participants or number of observations for repeated measures (postpartum depressive symptoms, weight and related measures, and breastfeeding), for each comparison.
Cesarean delivery includes both emergency and scheduled cesarean delivery.
Gestational weight gain (GWG) adequacy was defined as ratio of observed GWG versus GWG recommended in line with Institute of Medicine 2009 guidelines. Severely inadequate GWG was defined as GWG adequacy ratio <70%, moderately inadequate as GWG adequacy ratio 70% to <90%, adequate as GWG adequacy ratio 90% to 125%, and excessive as GWG adequacy ratio > 125%.
Estimates pooled using random-effects meta-analysis (REML method), with Hartung-Knapp adjustment for standard errors.

Supplementary Table 13. Associations between sub-optimal gestational weight gain (GWG) versus adequate GWG and maternal cesarean delivery (using dataset including imputed values [sensitivity] versus main analytical method).

	Studies	RR or MD (95%CI)	τ^2	Egger's test P
**Severely inadequate GWG**				
**Cesarean delivery (imputed datasets)**	55	0.83 (0.77-0.88)	0.008	0.499
**Cesarean delivery (main analysis)**	55	0.82 (0.77-0.88)	0.010	0.466
**Moderately inadequate GWG**				
**Cesarean delivery (imputed datasets)**	55	0.88 (0.84-0.92)	0.004	0.899
**Cesarean delivery (main analysis)**	54	0.88 (0.84-0.92)	0.004	0.907
**Excessive GWG**				
**Cesarean delivery (imputed datasets)**	51	1.09 (1.06-1.12)	0.000	0.146
**Cesarean delivery (main analysis)**	50	1.10 (1.06-1.13)	0.000	0.105

BMI: Body mass index. RR: Risk ratio. MD: mean difference.
Values were imputed where data were missing for potential confounders or outcome, using multiple imputation with chained outcomes, by study.
Cesarean delivery includes both emergency and scheduled cesarean delivery.
Gestational weight gain (GWG) adequacy was defined as ratio of observed GWG versus GWG recommended in line with Institute of Medicine 2009 guidelines. Severely inadequate GWG was defined as GWG adequacy ratio <70%, moderately inadequate as GWG adequacy ratio 70% to <90%, adequate as GWG adequacy ratio 90% to 125%, and excessive as GWG adequacy ratio > 125%.
Gestational weight gain (GWG) adequacy was defined as ratio of observed GWG versus GWG recommended in line with Institute of Medicine 2009 guidelines. Severely inadequate GWG was defined as GWG adequacy ratio <70%, moderately inadequate as GWG adequacy ratio 70% to <90%, adequate as GWG adequacy ratio 90% to 125%, and excessive as GWG adequacy ratio > 125%.
Estimates pooled using random-effects meta-analysis (REML method), with Hartung-Knapp adjustment for standard errors.

Supplementary Table 14. P values for Egger's test assessment of small-study bias in meta-analyses.

	**Meta-analytic model**		
**Outcome**	**Severely inadequate GWG**	**Moderately inadequate GWG**	**Excessive GWG**
**Perinatal outcomes (RR)**			
Cesarean delivery	0.466	0.907	0.105
Emergency cesarean delivery	0.685	0.116	0.063
Perineal tears	0.392	0.870	0.208
Postpartum hemorrhage	0.094	0.602	0.233
Prolonged labor	**0.001**	**0.038**	**0.005**
**Psychosocial measures (RR)**			
Highest quintile of depressive symptom score	0.170	0.958	0.524
**Post-partum weight and related measures (MD)**			
Post-partum weight retained, kg	0.675	0.549	0.207
BMI, kg/m^2	0.252	0.055	0.489
Waist circumference, cm	0.213	0.692	0.628
Systolic blood pressure, mmHg	**0.006**	0.395	0.085
Diastolic blood pressure, mmHg	0.963	0.661	0.885
**Breastfeeding (RR)**			
Breastfeeding	0.296	0.834	*
Exclusive breastfeeding	0.437	0.220	0.159

*Estimate not calculated as convergence not achieved during tau estimation.

Supplementary Table 15. Associations between sub-optimal gestational weight gain (versus adequate gestational weight gain) and maternal perinatal and postpartum outcomes - analyses done including only studies with overall low risk of bias (ROB).

	Severely inadequate			Moderately inadequate			Excessive		
	**Studies**	**RR or MD (95%CI)**	**τ^2**	**Studies**	**RR or MD (95%CI)**	**τ^2**	**Studies**	**RR or MD (95%CI)**	**τ^2**
**Perinatal outcomes (RR)**									
**Cesarean delivery**	**48**	**0.82 (0.77-0.87)**	**0.007**	**47**	**0.87 (0.81-0.94)***	**0.024**	**44**	**1.10 (1.06-1.15)**	**0.001**
**Emergency cesarean delivery**	**14**	**0.73 (0.54-0.99)**	**0.131**	**14**	**0.85 (0.73-0.99)**	**0.000**	**13**	**1.22 (1.03-1.45)**	**0.000**
**Perineal tears**	8	0.95 (0.88-1.02)	0.000	8	0.90 (0.78-1.03)	0.012	8	1.06 (0.93-1.21)	0.008
**Postpartum hemorrhage**	**10**	**0.91 (0.86-0.96)**	**0.000**	9	0.97 (0.91-1.04)	0.000	10	0.96 (0.46-2.01)	0.648
**Prolonged labor**	8	0.77 (0.56-1.06)	0.075	8	0.90 (0.75-1.08)	0.017	8	0.78 (0.48-1.28)	0.139
**Psychosocial measures (RR)**									
**Highest quintile of depressive symptom score**	7	0.99 (0.94-1.04)	0.000	7	0.98 (0.93-1.03)	0.000	7	1.04 (0.98-1.11)	0.000
**Post-partum weight and related measures (MD)**									
**Postpartum weight retained, kg**	**28**	**-2.93 (-3.67--2.19)**	**2.480**	**28**	**-1.16 (-1.48--0.84)**	**0.466**	**28**	**1.94 (1.37-2.50)**	**1.412**
**BMI, kg/m^2**	**28**	**-1.16 (-1.42--0.90)**	**0.319**	**28**	**-0.81 (-1.01--0.60)**	**0.196**	**28**	**0.96 (0.67-1.24)**	**0.274**
**Waist circumference, cm**	3	-3.05 (-8.14-2.05)	1.744	3	-1.98 (-5.89-1.92)	1.722	3	0.85 (-0.56-2.26)	0.000
**Systolic blood pressure, mmHg**	14	-1.00 (-2.00-0.00)	1.565	**14**	**-0.89 (-1.52--0.25)**	**0.385**	14	0.74 (-0.28-1.75)	0.636
**Diastolic blood pressure, mmHg**	**14**	**-0.60 (-0.89--0.32)**	**0.019**	**14**	**-0.50 (-0.88--0.11)**	**0.077**	14	1.65 (0.00-3.31)	5.982
**Breastfeeding (RR)**									
**Currently any breastfeeding**	12	1.00 (1.00-1.00)	0.000	12	1.00 (1.00-1.00)	0.000	12	1.00 (1.00-1.00)	0.000
**Currently exclusively breastfeeding**	8	0.98 (0.93-1.04)	0.002	8	1.00 (0.98-1.02)	0.000	8	1.00 (0.99-1.01)	0.000

Low overall ROB defined as low ROB in all domains, or low ROB in three domains plus moderate ROB in one domain.
Gestational weight gain (GWG) adequacy was defined as ratio of observed GWG versus GWG recommended in line with Institute of Medicine 2009 guidelines. Severely inadequate GWG was defined as GWG adequacy ratio <70%, moderately inadequate as GWG adequacy ratio 70% to <90%, adequate as GWG adequacy ratio 90% to 125%, and excessive as GWG adequacy ratio > 125%.
Estimates pooled using random-effects meta-analysis (REML method), with Hartung-Knapp adjustment for standard errors. *Random effects Sidik-Jonkman method was used for this particular meta-analysis to enable convergence of estimates for τ^2.

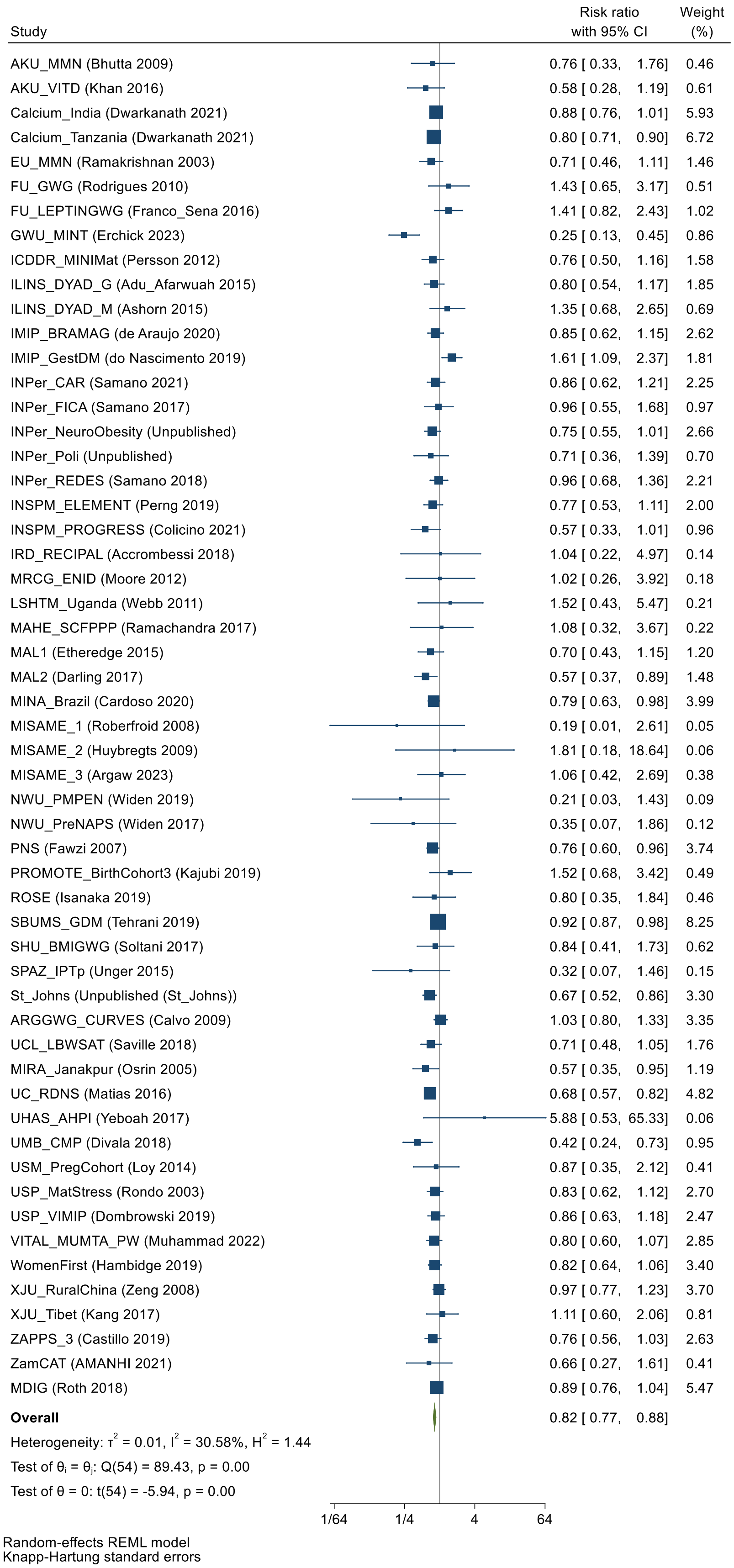

Supplementary Figure 1. Forest plot of association between severely inadequate gestational weight gain and risk of cesarean delivery.

Study	Risk ratio with 95% CI	Weight (%)
AKU_MMN (Bhutta 2009)	1.00 [0.36, 2.75]	0.24
AKU_VITD (Khan 2016)	0.91 [0.49, 1.68]	0.63
Calcium_India (Dwarkanath 2021)	0.95 [0.88, 1.02]	11.08
Calcium_Tanzania (Dwarkanath 2021)	0.82 [0.73, 0.91]	8.46
EU_MMN (Ramakrishnan 2003)	0.81 [0.52, 1.26]	1.18
FU_GWG (Rodrigues 2010)	1.24 [0.52, 2.96]	0.32
FU_LEPTINGWG (Franco_Sena 2016)	0.79 [0.35, 1.77]	0.37
GWU_MINT (Erchick 2023)	0.90 [0.57, 1.42]	1.10
ICDDR_MINIMat (Persson 2012)	0.98 [0.61, 1.58]	1.00
ILINS_DYAD_G (Adu_Afarwuah 2015)	0.93 [0.62, 1.41]	1.32
ILINS_DYAD_M (Ashorn 2015)	1.62 [0.81, 3.21]	0.51
IMIP_BRAMAG (de Araujo 2020)	0.95 [0.73, 1.23]	2.87
IMIP_GestDM (do Nascimento 2019)	1.54 [1.04, 2.29]	1.44
INPer_CAR (Samano 2021)	0.68 [0.44, 1.05]	1.18
INPer_FICA (Samano 2017)	0.96 [0.60, 1.55]	1.01
INPer_NeuroObesity (Unpublished)	0.73 [0.46, 1.17]	1.08
INPer_Poli (Unpublished)	0.92 [0.45, 1.90]	0.46
INPer_REDES (Samano 2018)	0.92 [0.64, 1.32]	1.68
INSPM_ELEMENT (Perng 2019)	0.84 [0.64, 1.12]	2.54
INSPM_PROGRESS (Colicino 2021)	0.91 [0.71, 1.15]	3.32
IRD_RECIPAL (Accrombessi 2018)	2.24 [0.33, 15.09]	0.07
LSHTM_Uganda (Webb 2011)	1.00 [0.55, 1.84]	0.65
MAHE_SCFPPP (Ramachandra 2017)	0.29 [0.04, 1.94]	0.07
MAL1 (Etheredge 2015)	0.63 [0.38, 1.05]	0.90
MAL2 (Darling 2017)	0.60 [0.34, 1.05]	0.73
MINA_Brazil (Cardoso 2020)	0.91 [0.74, 1.12]	4.12
MISAME_1 (Roberfroid 2008)	0.81 [0.07, 9.90]	0.04
MISAME_3 (Argaw 2023)	1.00 [0.33, 3.06]	0.20
NWU_PMPEN (Widen 2019)	0.78 [0.12, 5.22]	0.07
NWU_PreNAPS (Widen 2017)	0.65 [0.11, 3.99]	0.08
PNS (Fawzi 2007)	0.70 [0.55, 0.88]	3.54
PROMOTE_BirthCohort3 (Kajubi 2019)	1.05 [0.38, 2.92]	0.24
ROSE (Isanaka 2019)	1.26 [0.57, 2.78]	0.39
SBUMS_GDM (Tehrani 2019)	0.98 [0.92, 1.04]	12.24
SHU_BMIGWG (Soltani 2017)	1.38 [0.66, 2.89]	0.44
SPAZ_IPTp (Unger 2015)	1.03 [0.43, 2.45]	0.32
St_Johns (Unpublished (St_Johns))	0.81 [0.64, 1.01]	3.73
ARGGWG_CURVES (Calvo 2009)	1.07 [0.79, 1.45]	2.26
UCL_LBWSAT (Saville 2018)	0.62 [0.44, 0.87]	1.91
MIRA_Janakpur (Osrin 2005)	0.68 [0.41, 1.13]	0.89
UC_RDNS (Matias 2016)	0.80 [0.65, 0.98]	4.15
UHAS_AHPI (Yeboah 2017)	8.11 [0.67, 98.71]	0.04
UMB_CMP (Divala 2018)	0.71 [0.39, 1.31]	0.65
USM_PregCohort (Loy 2014)	0.42 [0.12, 1.53]	0.15
USP_MatStress (Rondo 2003)	0.74 [0.55, 1.01]	2.25
USP_VIMIP (Dombrowski 2019)	0.89 [0.63, 1.25]	1.89
UZ_MatNutri (Friis 2004)	0.42 [0.08, 2.30]	0.09
VITAL_MUMTA_PW (Muhammad 2022)	0.90 [0.65, 1.23]	2.12
WomenFirst (Hambidge 2019)	0.87 [0.65, 1.16]	2.53
XJU_RuralChina (Zeng 2008)	0.89 [0.66, 1.21]	2.30
XJU_Tibet (Kang 2017)	1.30 [0.69, 2.45]	0.60
ZAPPS_3 (Castillo 2019)	0.77 [0.54, 1.09]	1.77
ZamCAT (AMANHI 2021)	1.05 [0.58, 1.88]	0.69
MDIG (Roth 2018)	0.83 [0.71, 0.97]	6.10
**Overall**	0.88 [0.84, 0.92]	

Heterogeneity: τ^2 = 0.00, I^2 = 19.21%, H^2 = 1.24

Test of $\theta_i = \theta_j$: Q(53) = 59.23, p = 0.26

Test of θ = 0: t(53) = -5.53, p = 0.00

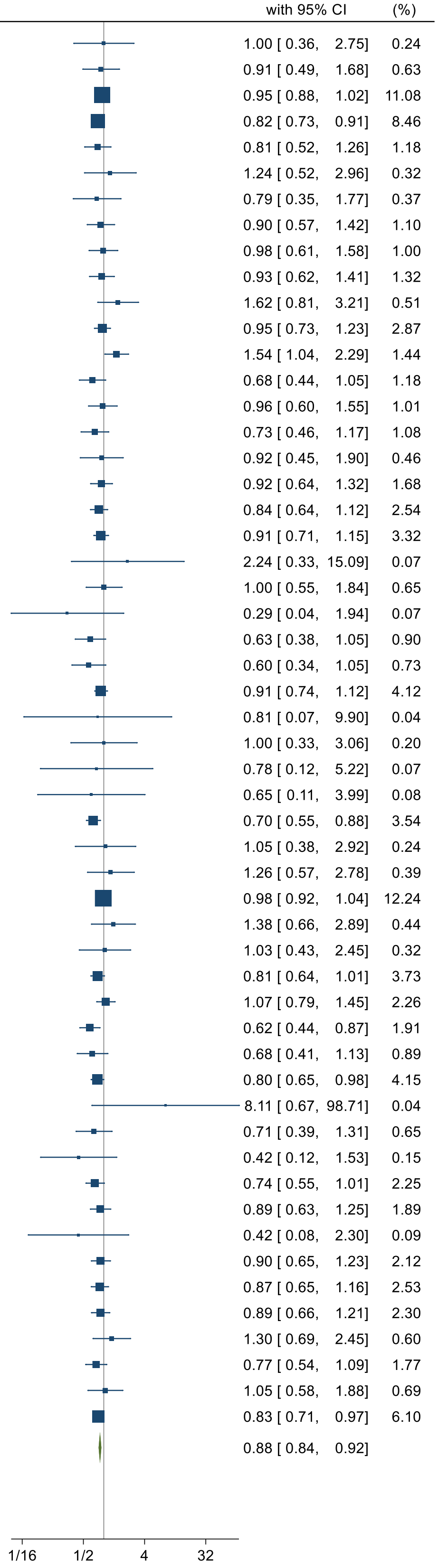

Random-effects REML model
Knapp-Hartung standard errors

Supplementary Figure 2. Forest plot of association between moderately inadequate gestational weight gain and risk of cesarean delivery.

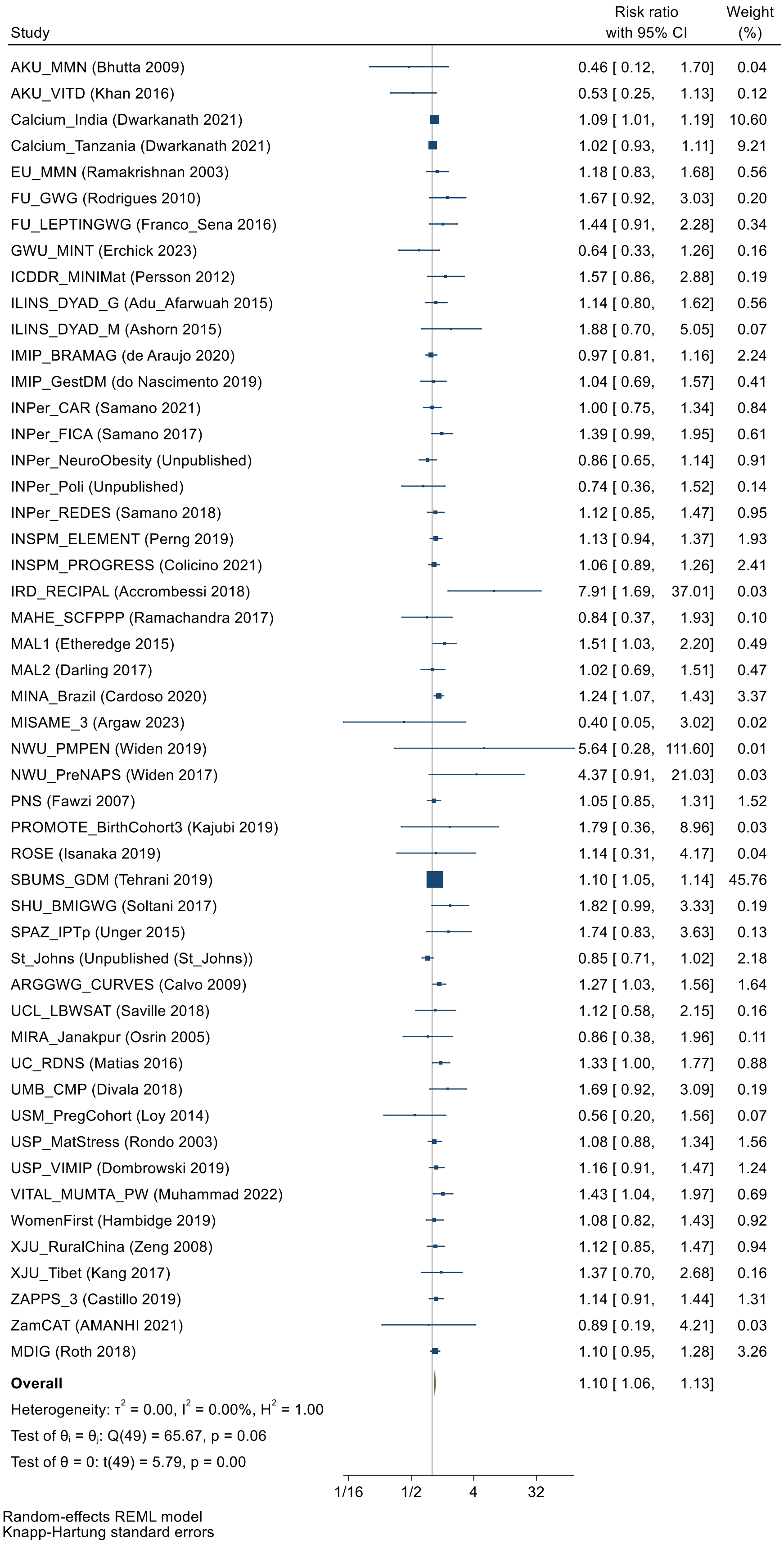

Supplementary Figure 3. Forest plot of association between excessive gestational weight gain and risk of cesarean delivery.

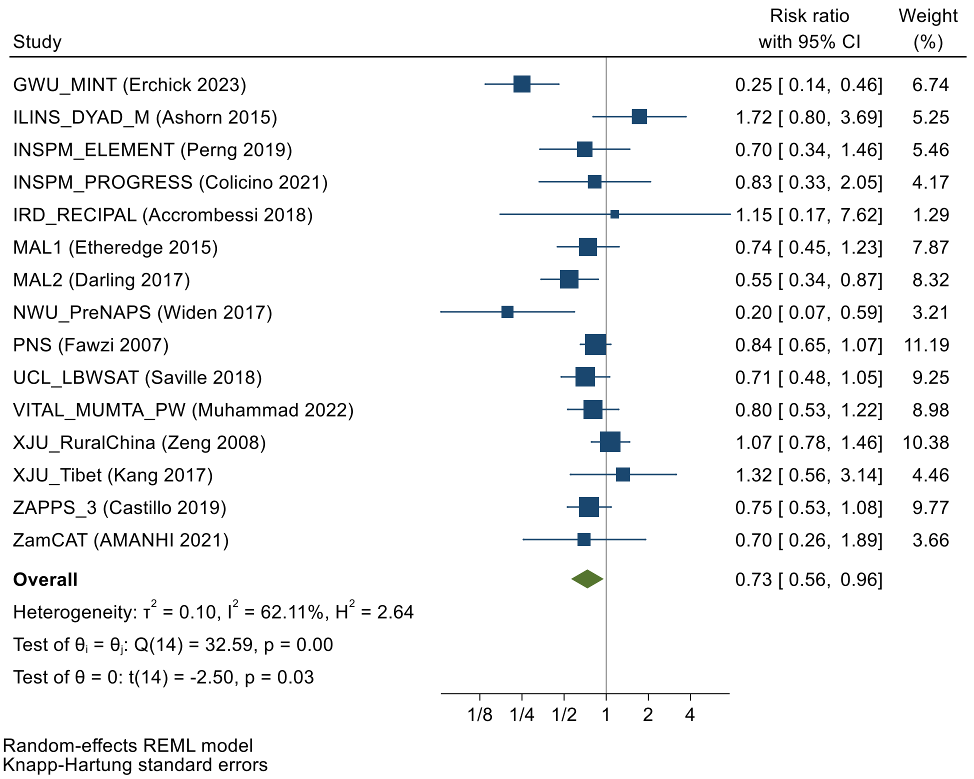

Supplementary Figure 4. Forest plot of association between severely inadequate gestational weight gain and risk of emergency cesarean delivery.

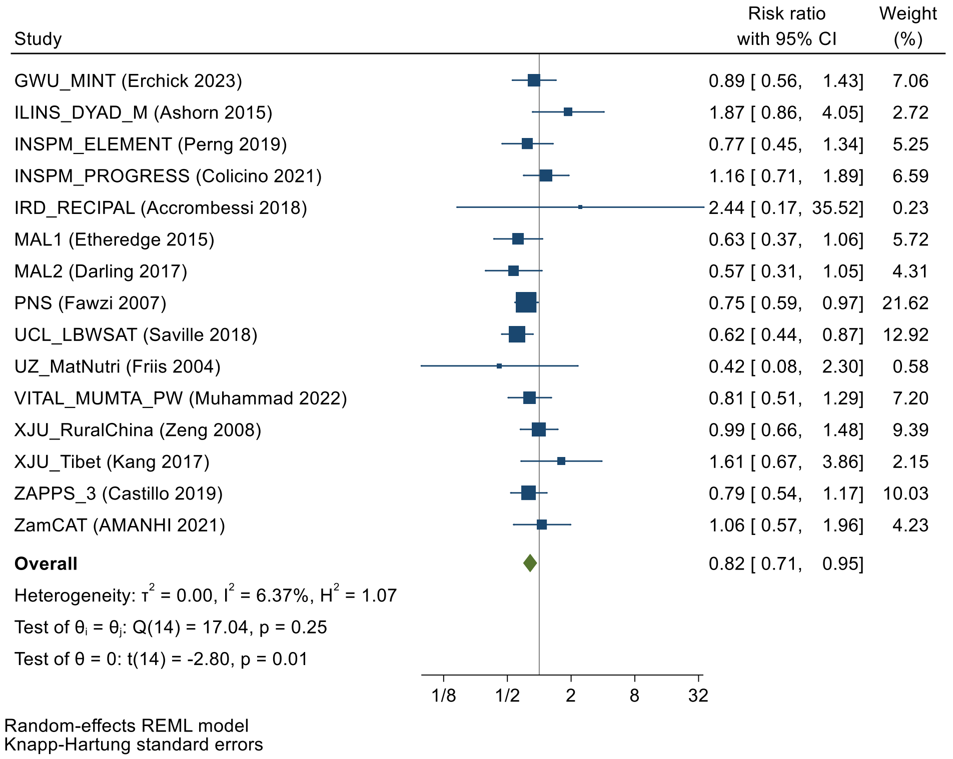

Supplementary Figure 5. Forest plot of association between moderately inadequate gestational weight gain and risk of emergency cesarean delivery.

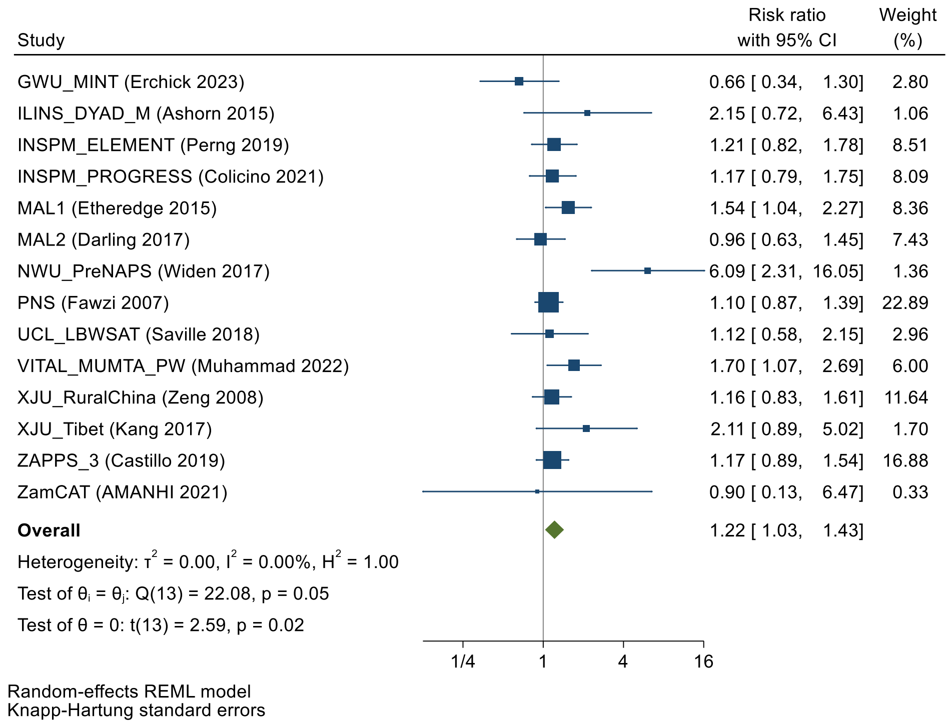

Supplementary Figure 6. Forest plot of association between excessive gestational weight gain and risk of emergency cesarean delivery.

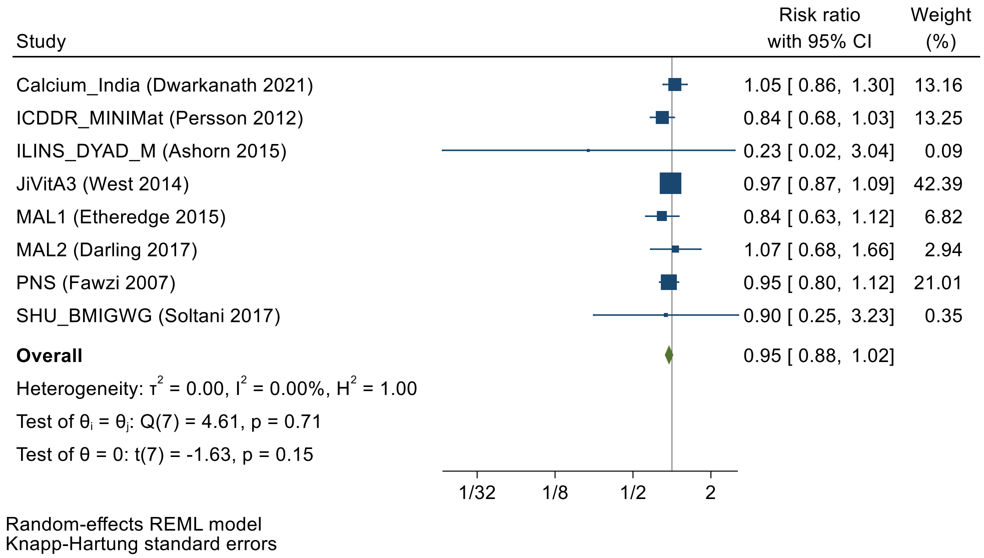

Supplementary Figure 7. Forest plot of association between severely inadequate gestational weight gain and risk of perineal tears.

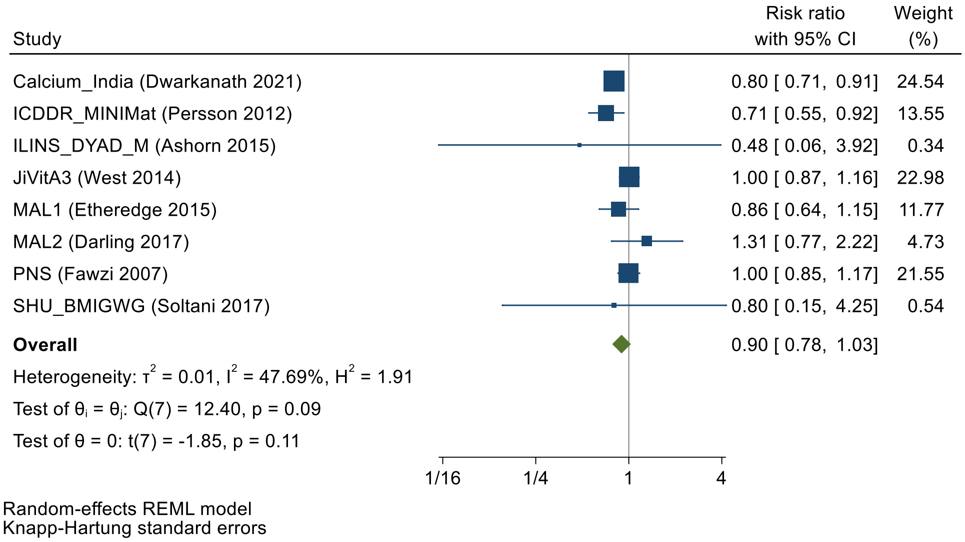

Supplementary Figure 8. Forest plot of association between moderately inadequate gestational weight gain and risk of perineal tears.

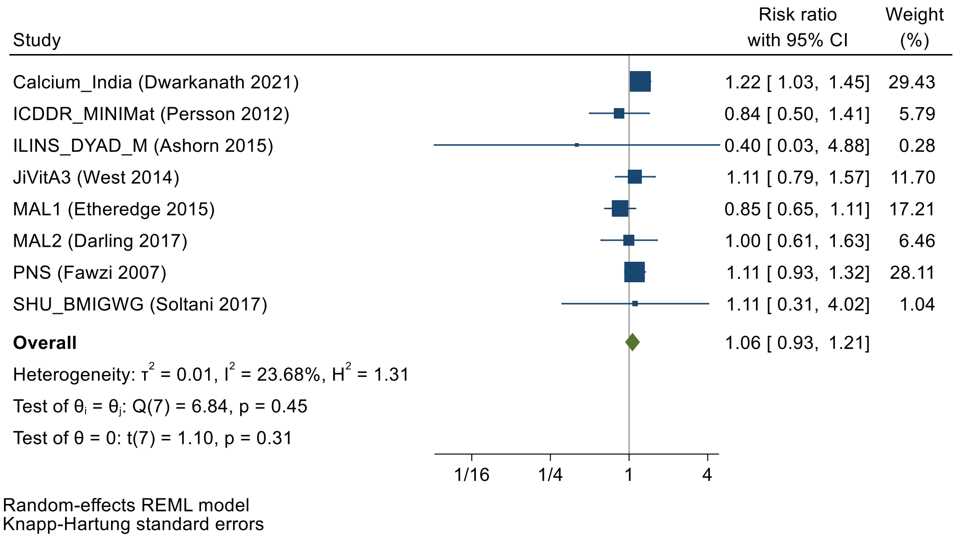

Supplementary Figure 9. Forest plot of association between excessive gestational weight gain and risk of perineal tears.

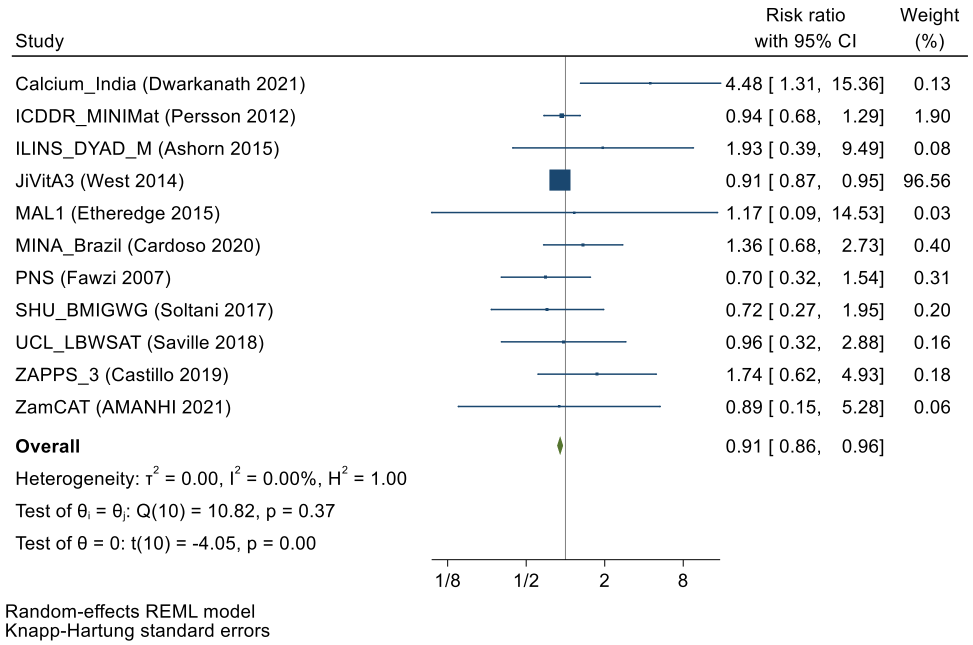

Supplementary Figure 10. Forest plot of association between severely inadequate gestational weight gain and risk of postpartum hemorrhage.

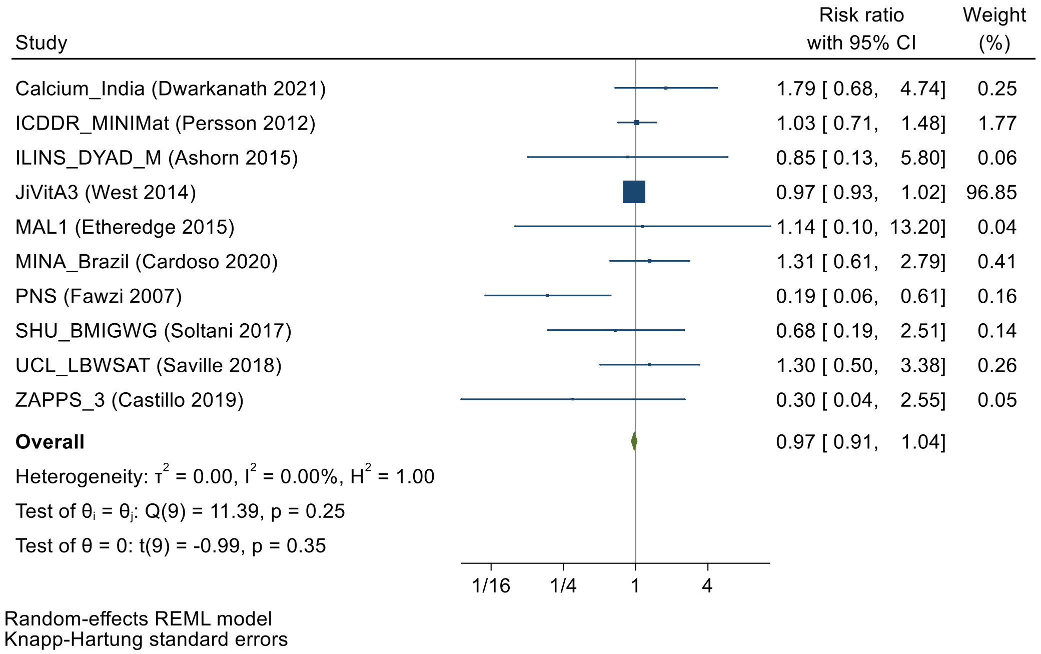

Supplementary Figure 11. Forest plot of association between moderately inadequate gestational weight gain and risk of postpartum hemorrhage.

Supplemental material BMJ Publishing Group Limited (BMJ) disclaims all liability and responsibility arising from any reliance placed on this supplemental material which has been supplied by the author(s)

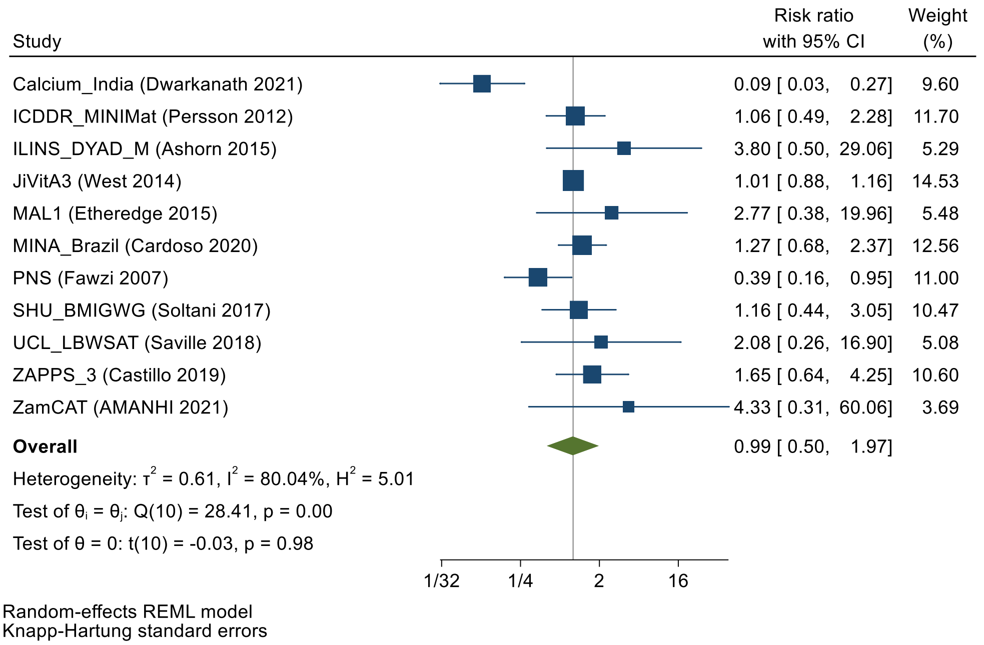

Supplementary Figure 12. Forest plot of association between excessive gestational weight gain and risk of postpartum hemorrhage.

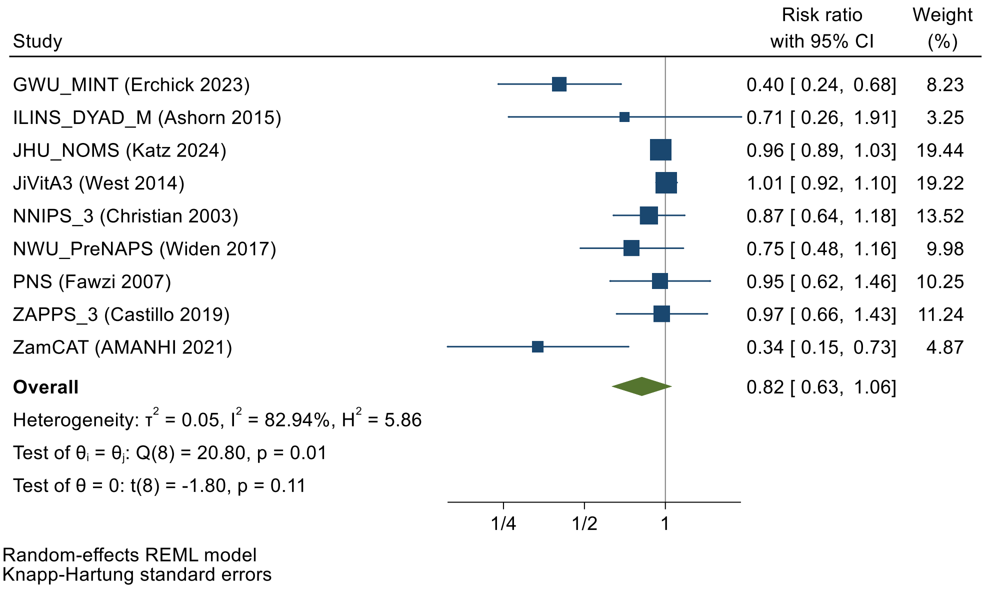

Supplementary Figure 13. Forest plot of association between severely inadequate gestational weight gain and risk of prolonged labor.

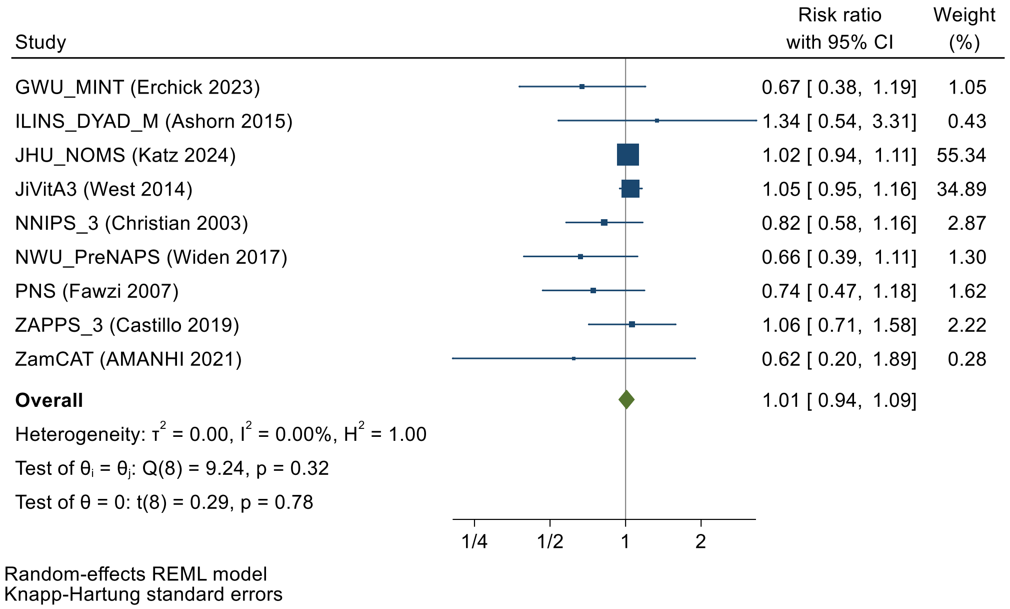

Supplementary Figure 14. Forest plot of association between moderately inadequate gestational weight gain and risk of prolonged labor.

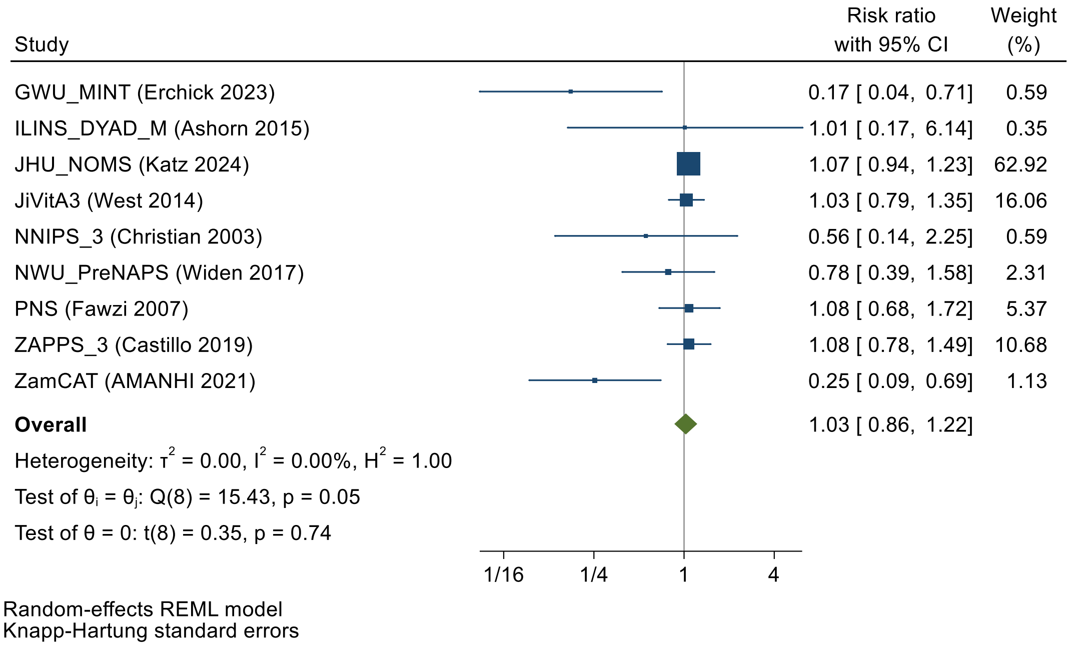

Supplementary Figure 15. Forest plot of association between excessive gestational weight gain and risk of prolonged labor.

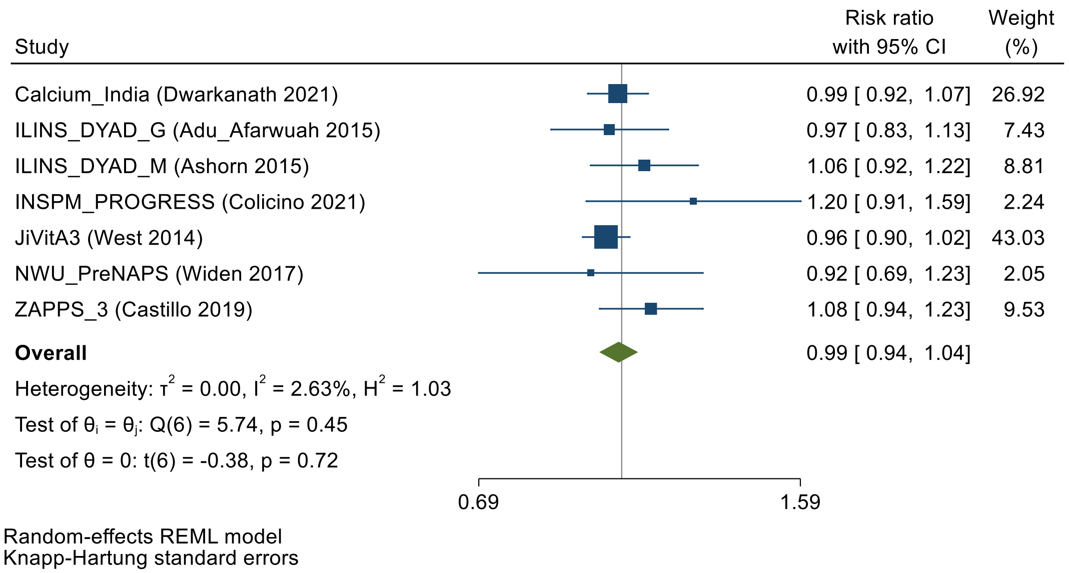

Supplementary Figure 16. Forest plot of association between severely inadequate gestational weight gain and risk of being in the highest quintile of depressive symptom scores.

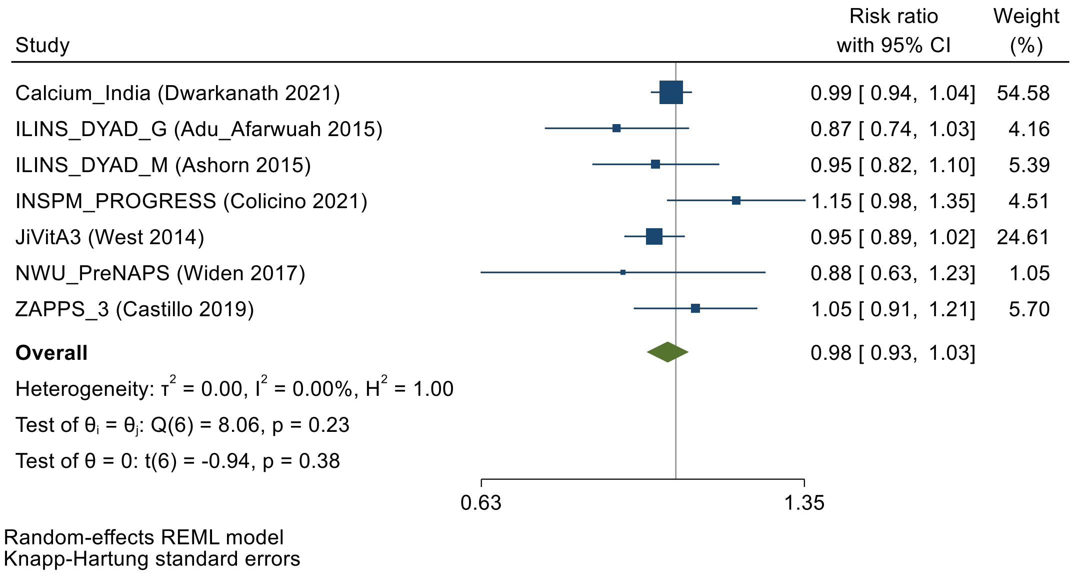

Supplementary Figure 17. Forest plot of association between moderately inadequate gestational weight gain and risk of being in the highest quintile of depressive symptom scores.

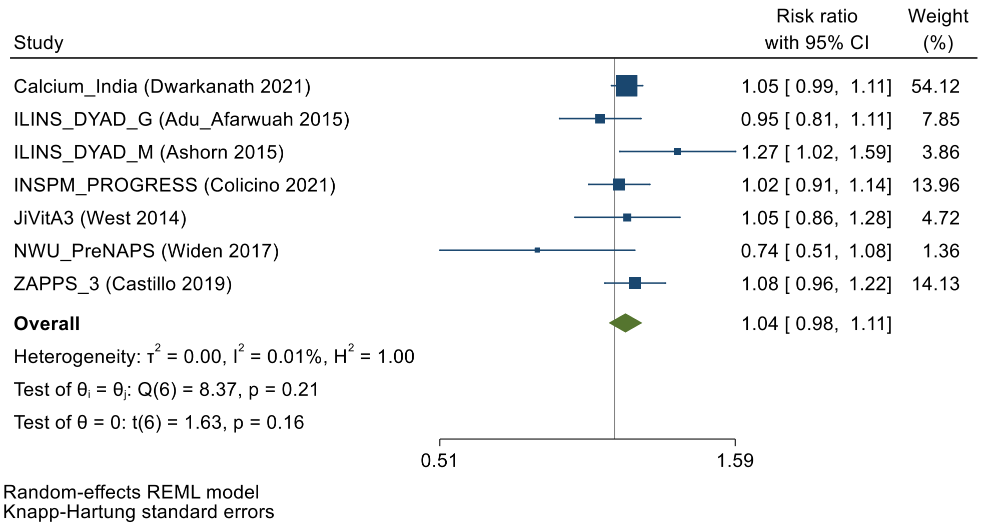

Supplementary Figure 18. Forest plot of association between excessive gestational weight gain and risk of being in the highest quintile of depressive symptom scores.

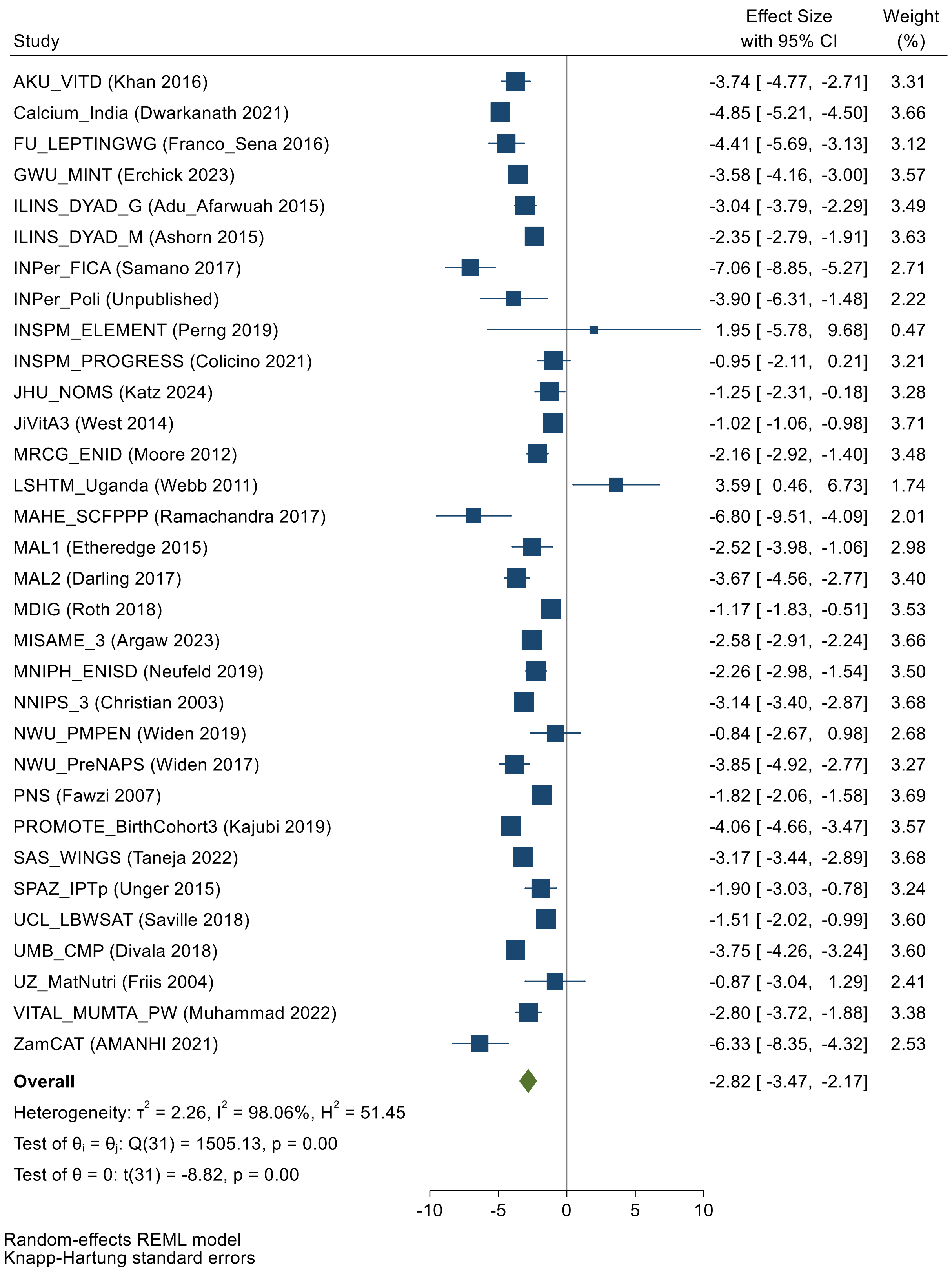

Supplementary Figure 19. Forest plot of association between severely inadequate gestational weight gain and postpartum weight retained (kg).

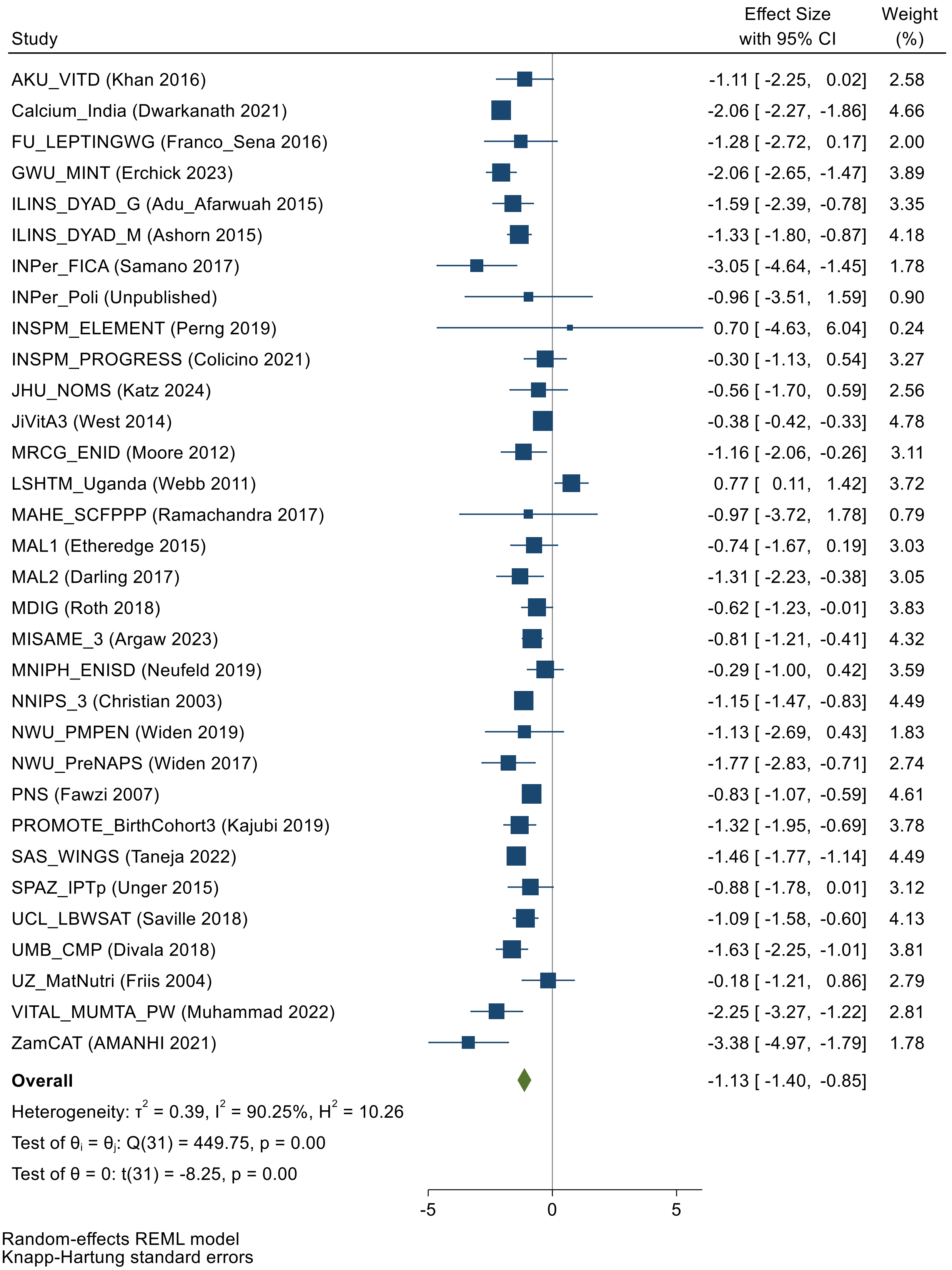

Supplementary Figure 20. Forest plot of association between moderately inadequate gestational weight gain and postpartum weight retained (kg).

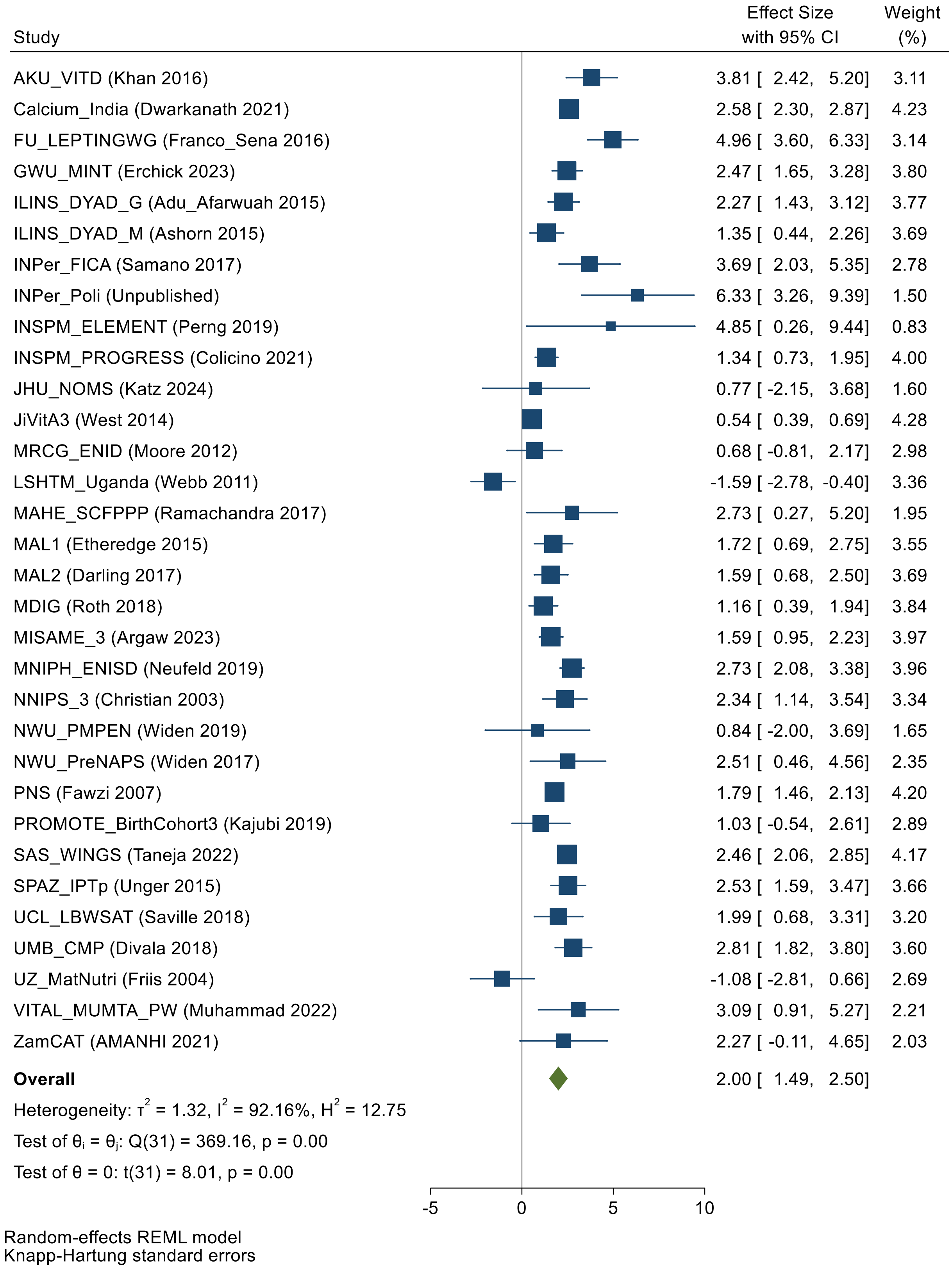

Supplementary Figure 21. Forest plot of association between excessive gestational weight gain and postpartum weight retained (kg).

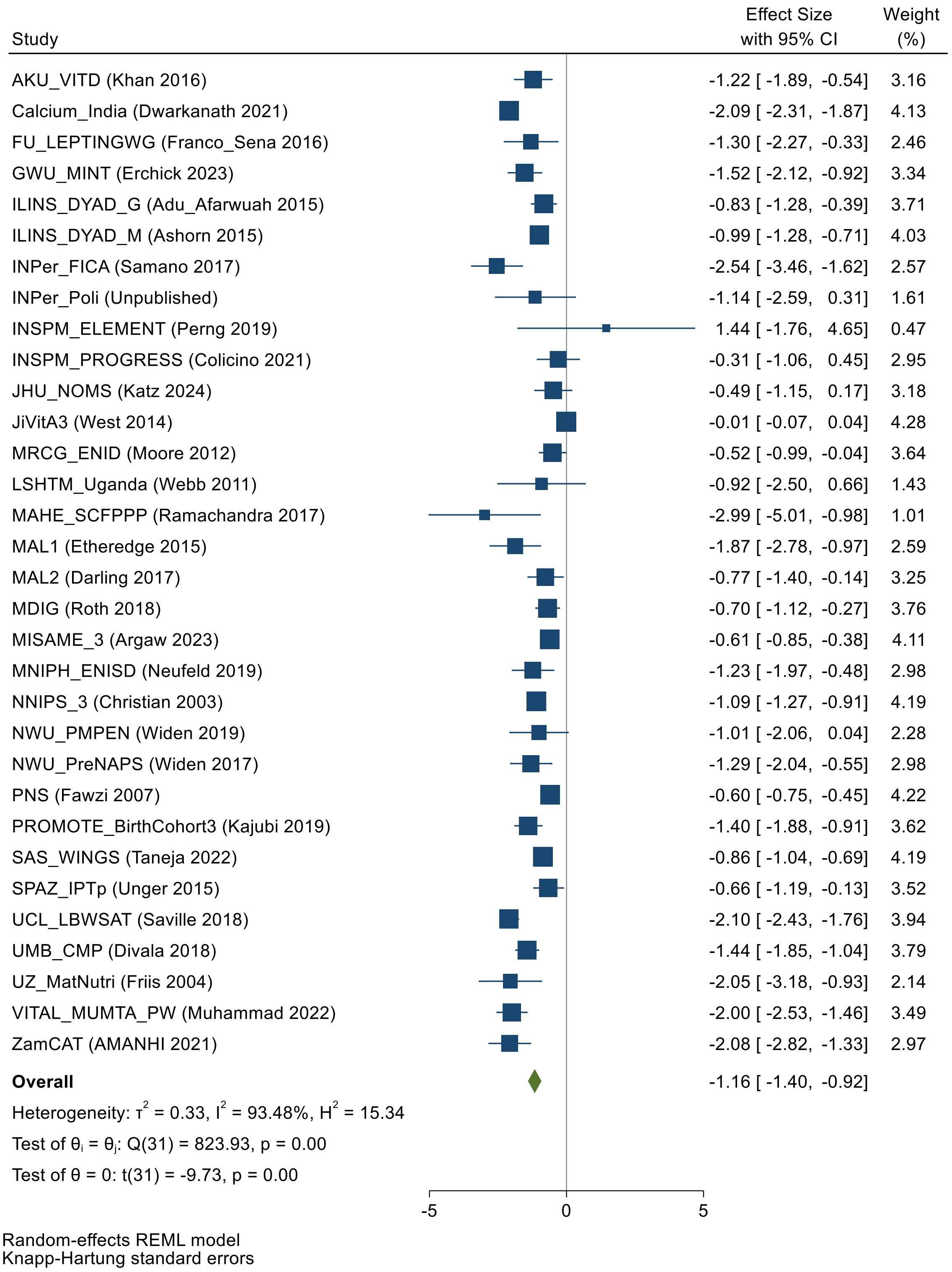

Supplementary Figure 22. Forest plot of association between severely inadequate gestational weight gain and postpartum body mass index (kg/m²).

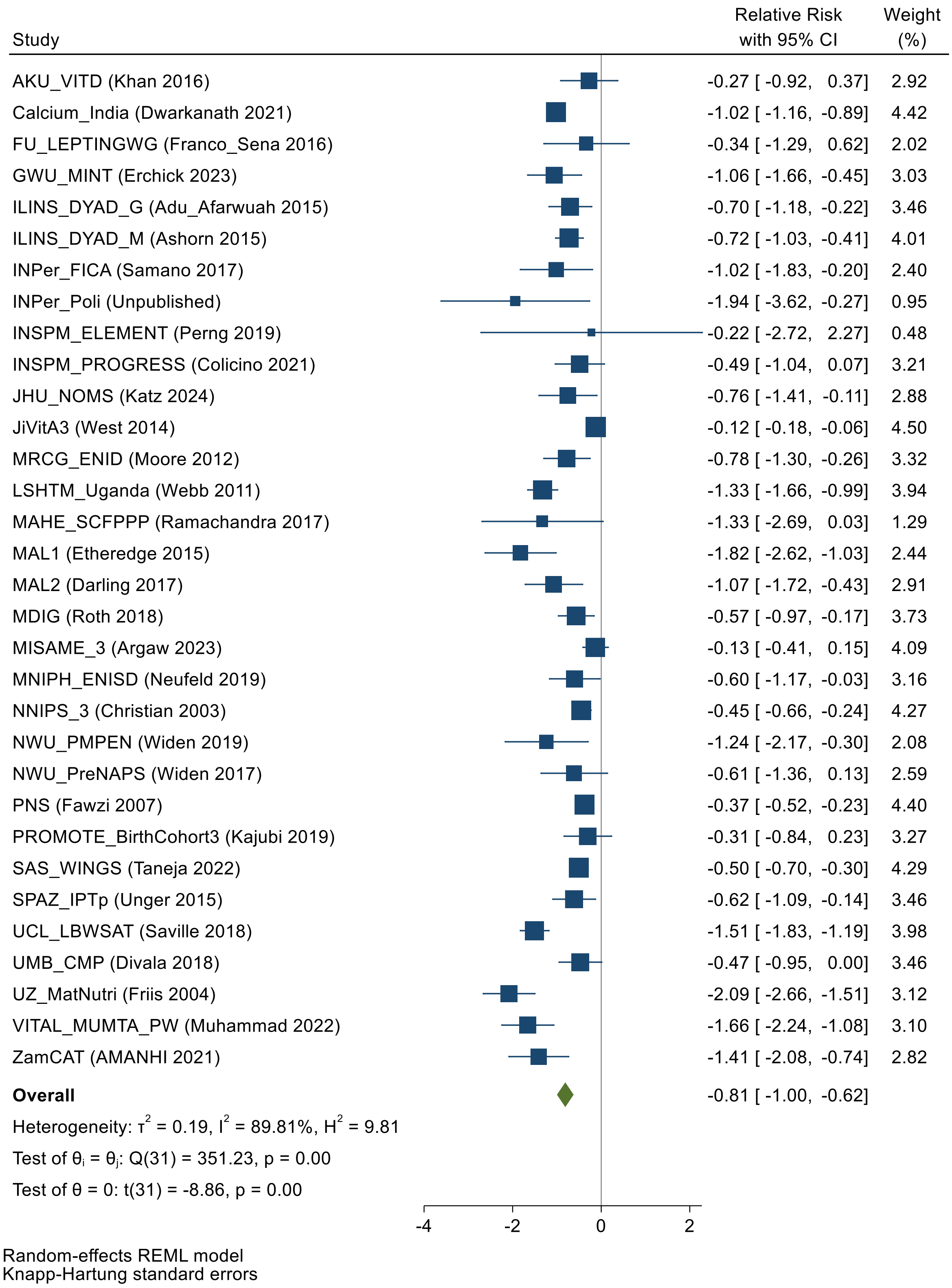

Supplementary Figure 23. Forest plot of association between moderately inadequate gestational weight gain and postpartum body mass index (kg/m²).

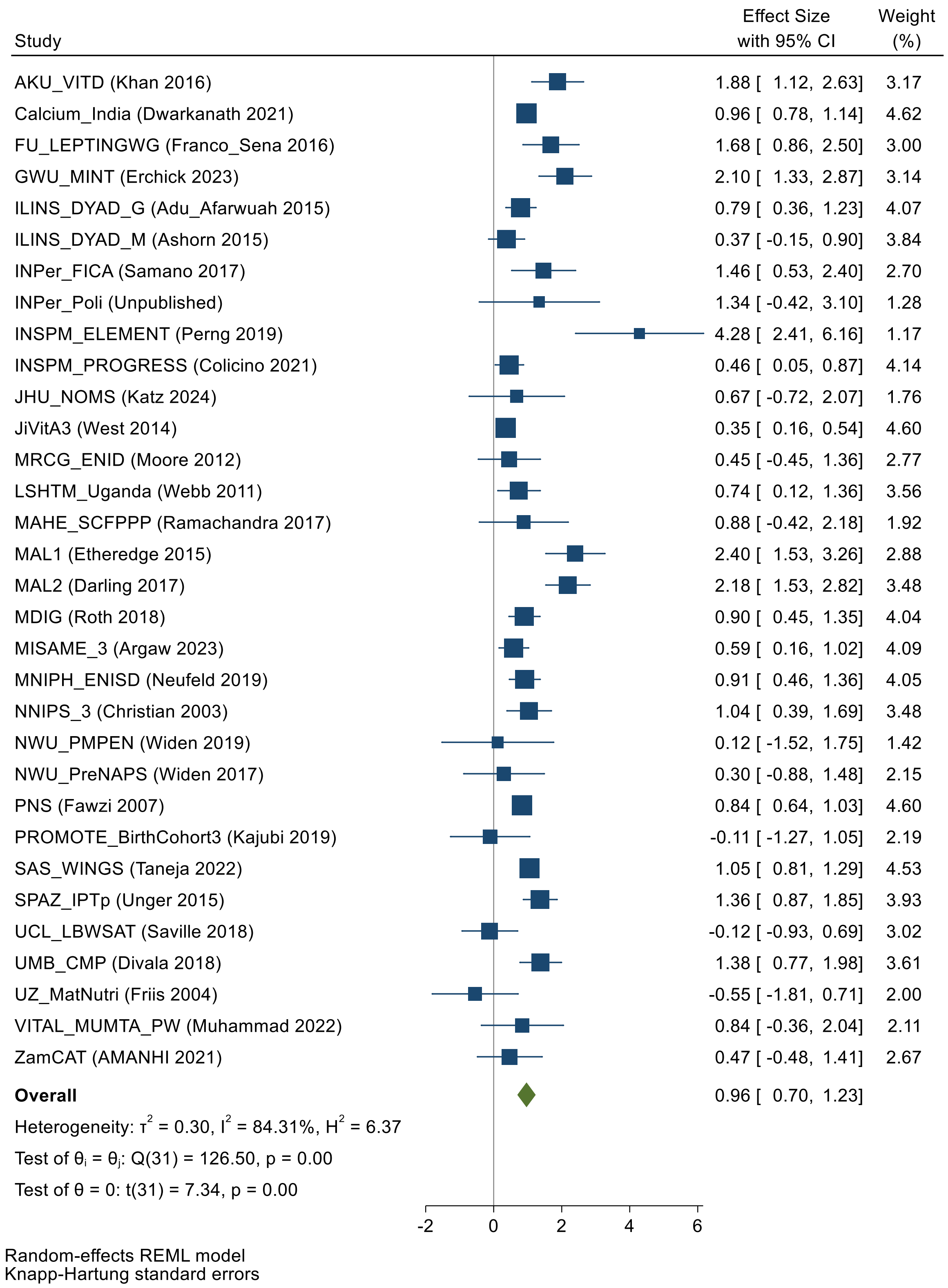

Supplementary Figure 24. Forest plot of association between excessive gestational weight gain and postpartum body mass index (kg/m²).

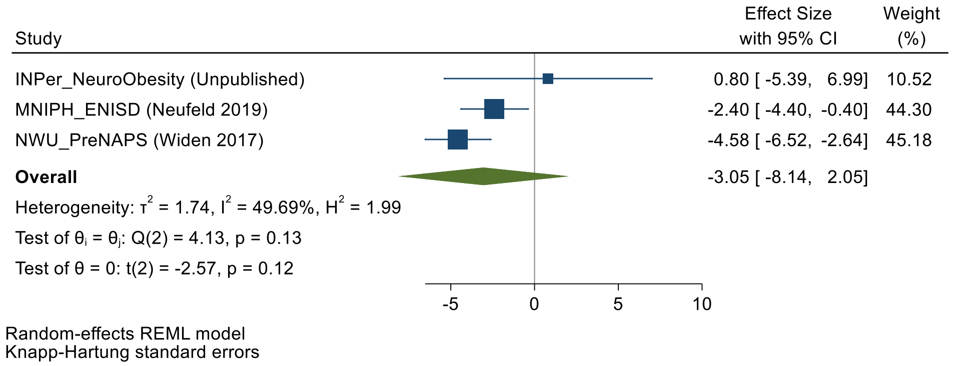

Supplementary Figure 25. Forest plot of association between severely inadequate gestational weight gain and postpartum waist circumference (cm).

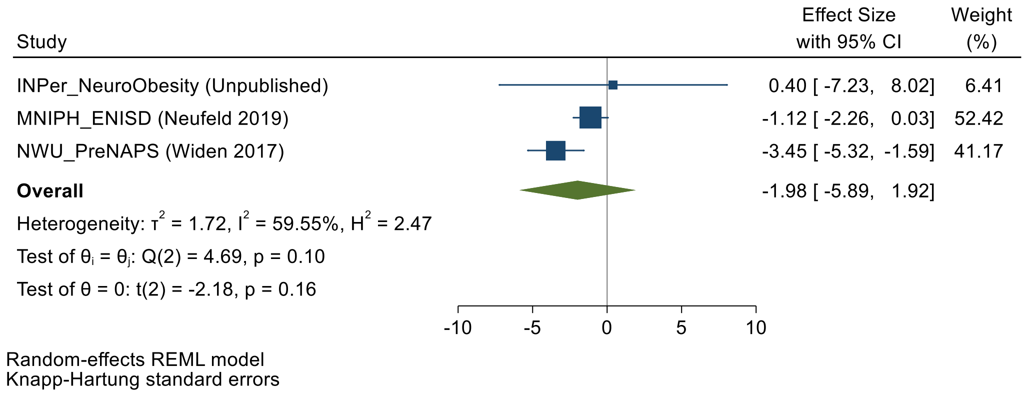

Supplementary Figure 26. Forest plot of association between moderately inadequate gestational weight gain and postpartum waist circumference (cm).

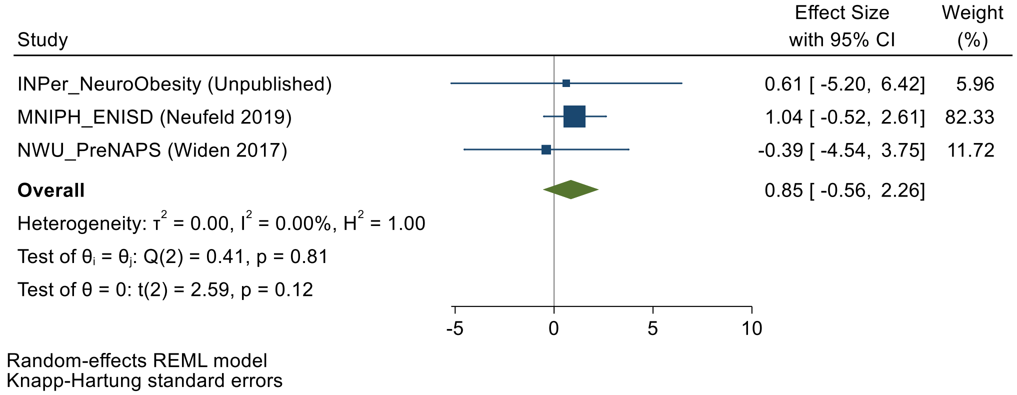

Supplementary Figure 27. Forest plot of association between excessive gestational weight gain and postpartum waist circumference (cm).

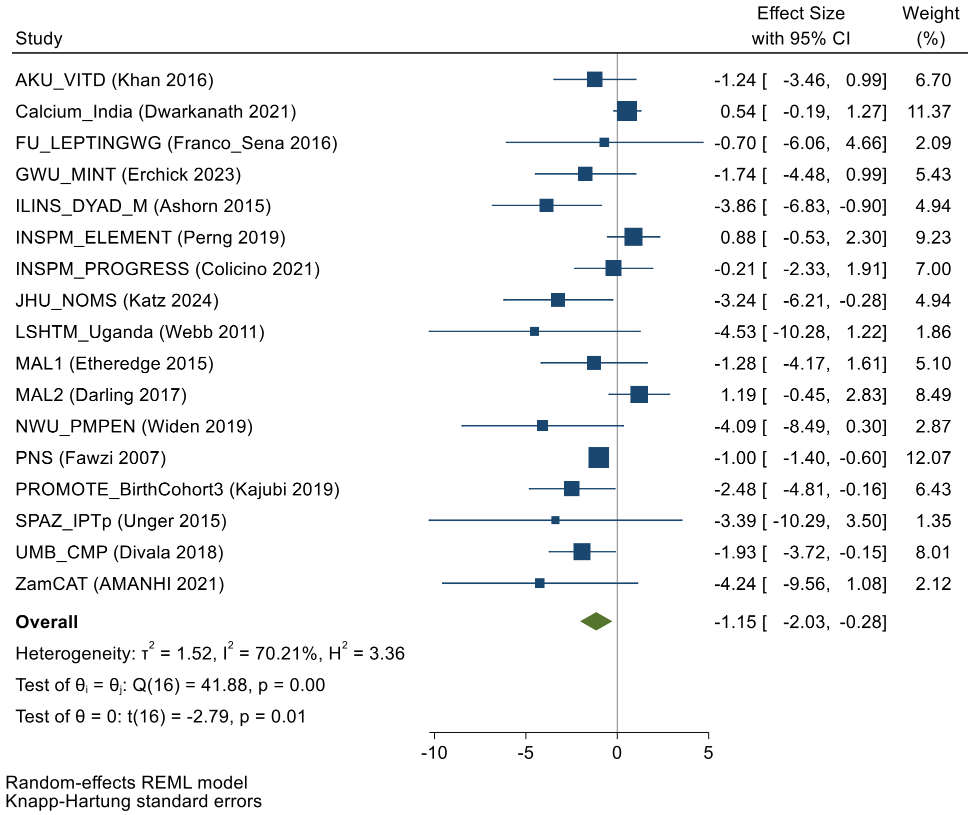

Supplementary Figure 28. Forest plot of association between severely inadequate gestational weight gain and postpartum systolic blood pressure (mmHg).

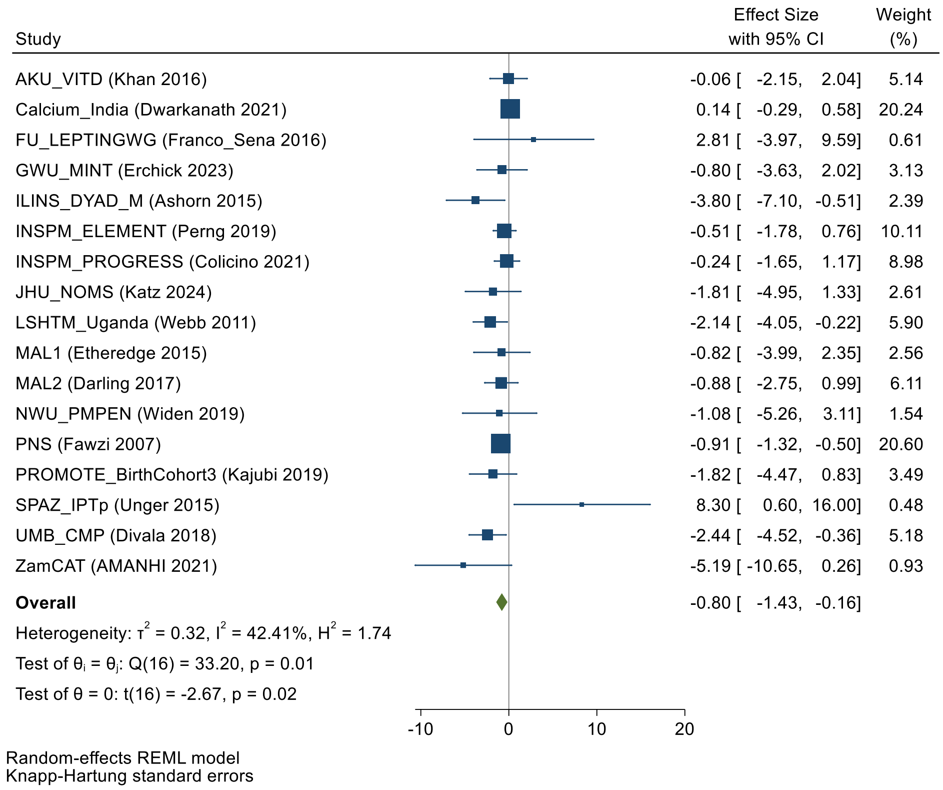

Supplementary Figure 29. Forest plot of association between moderately inadequate gestational weight gain and postpartum systolic blood pressure (mmHg).

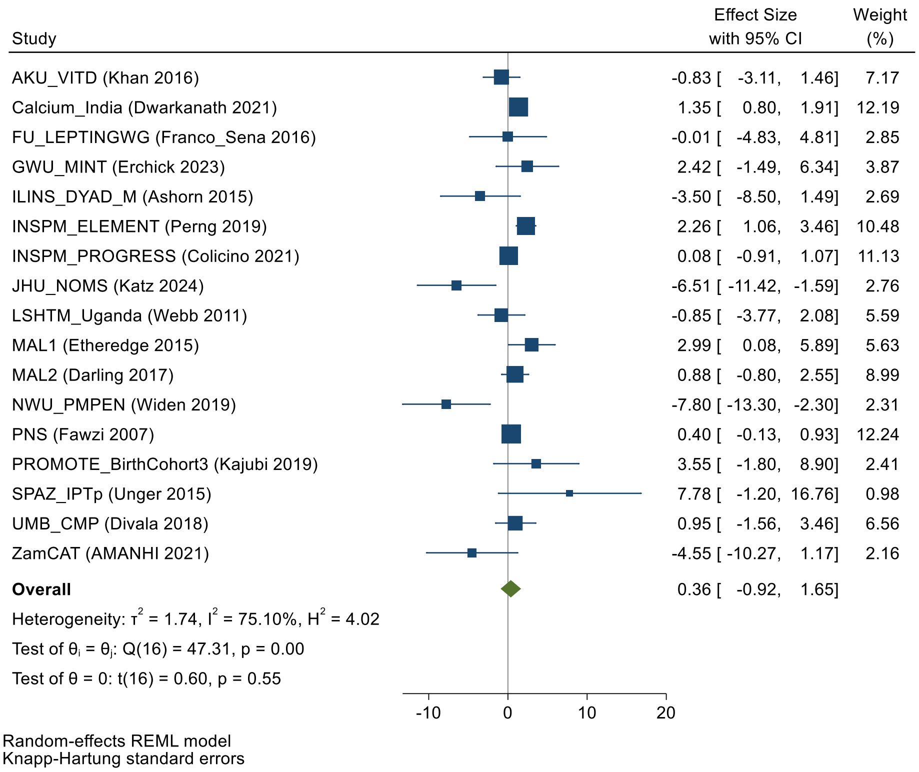

Supplementary Figure 30. Forest plot of association between excessive gestational weight gain and postpartum systolic blood pressure (mmHg).

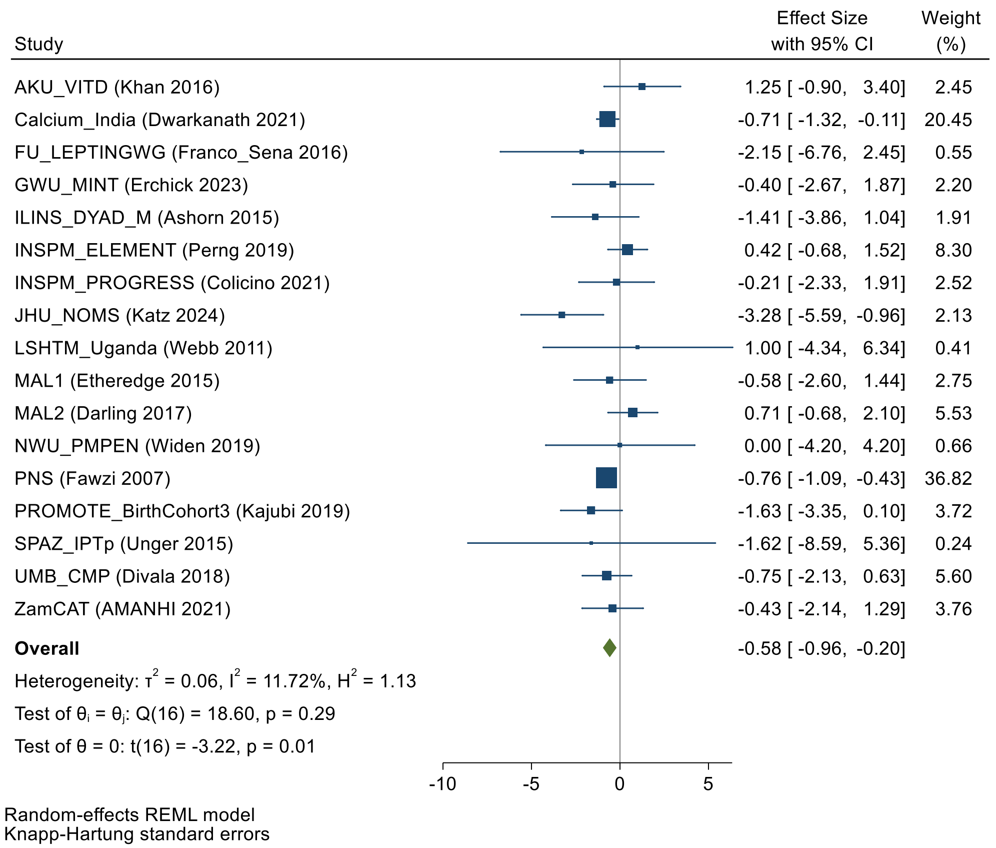

Supplementary Figure 31. Forest plot of association between severely inadequate gestational weight gain and postpartum diastolic blood pressure (mmHg).

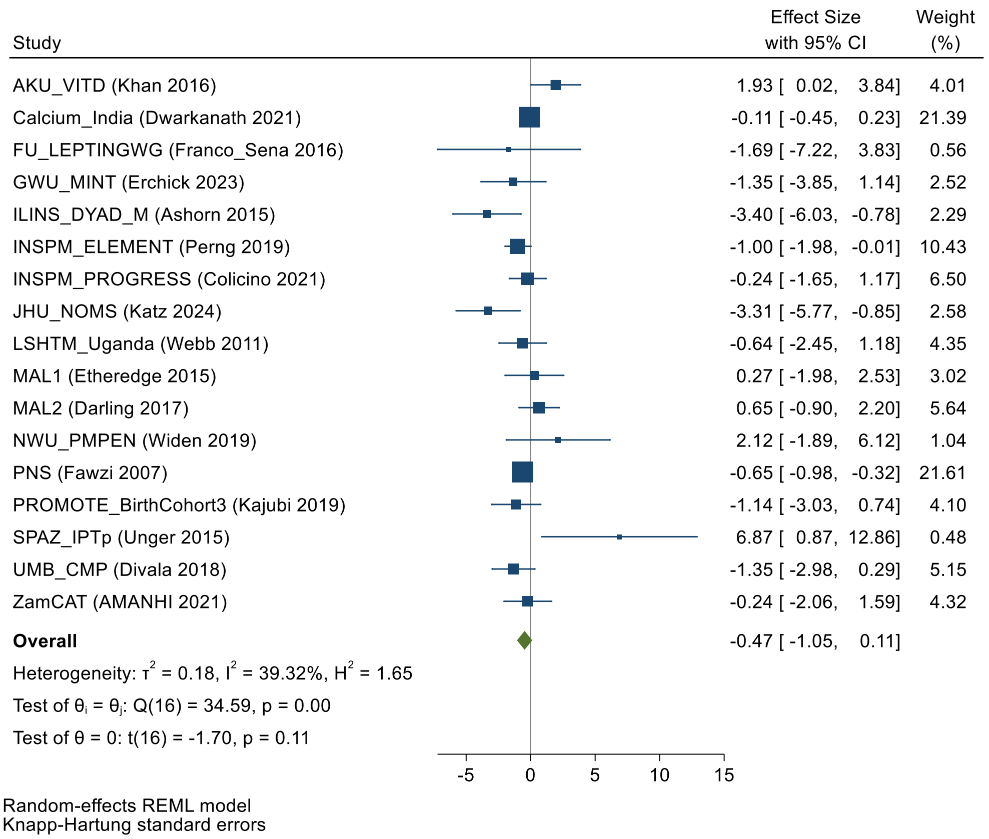

Supplementary Figure 32. Forest plot of association between moderately inadequate gestational weight gain and postpartum diastolic blood pressure (mmHg).

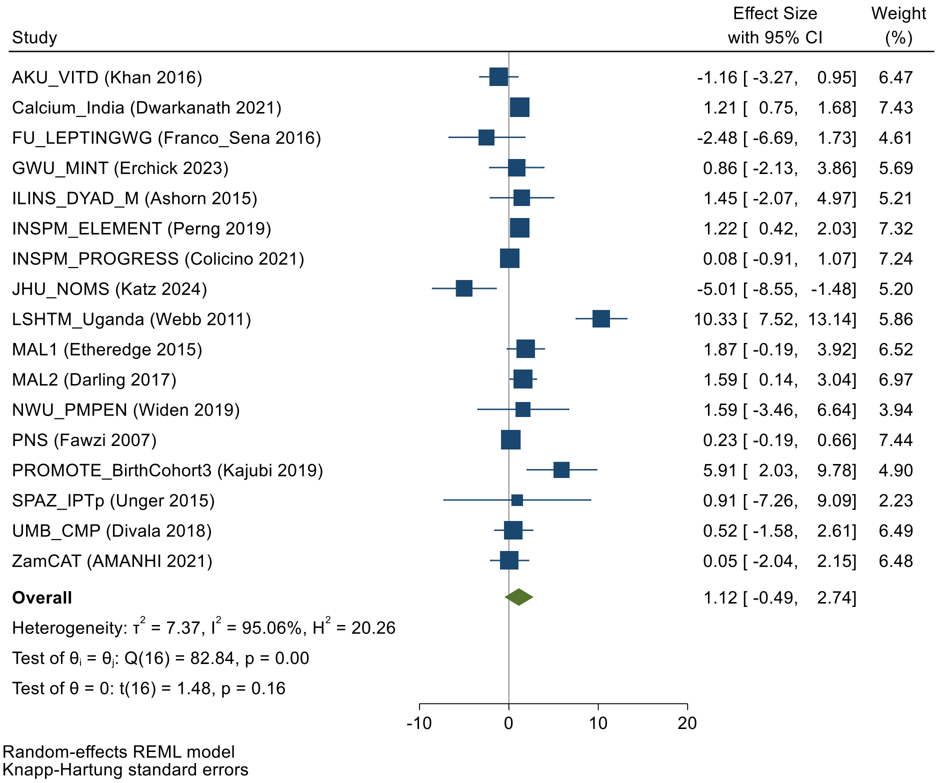

Supplementary Figure 33. Forest plot of association between excessive gestational weight gain and postpartum diastolic blood pressure (mmHg).

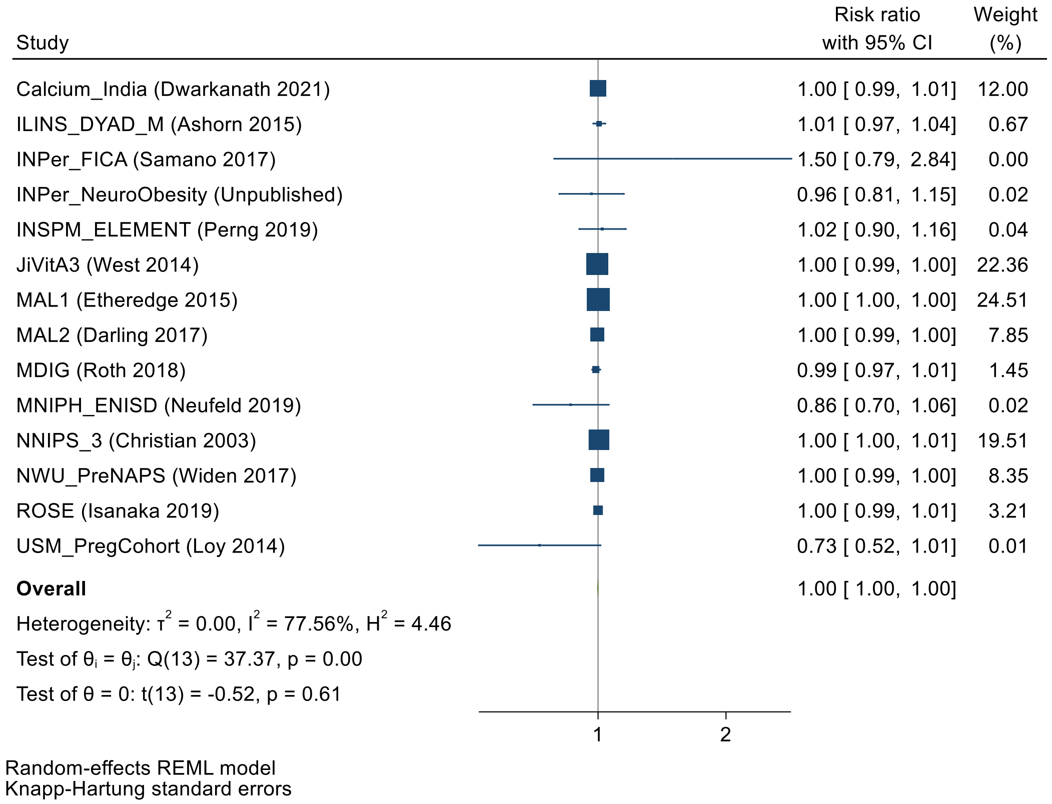

Supplementary Figure 34. Forest plot of association between severely inadequate gestational weight gain and likelihood of currently breastfeeding.

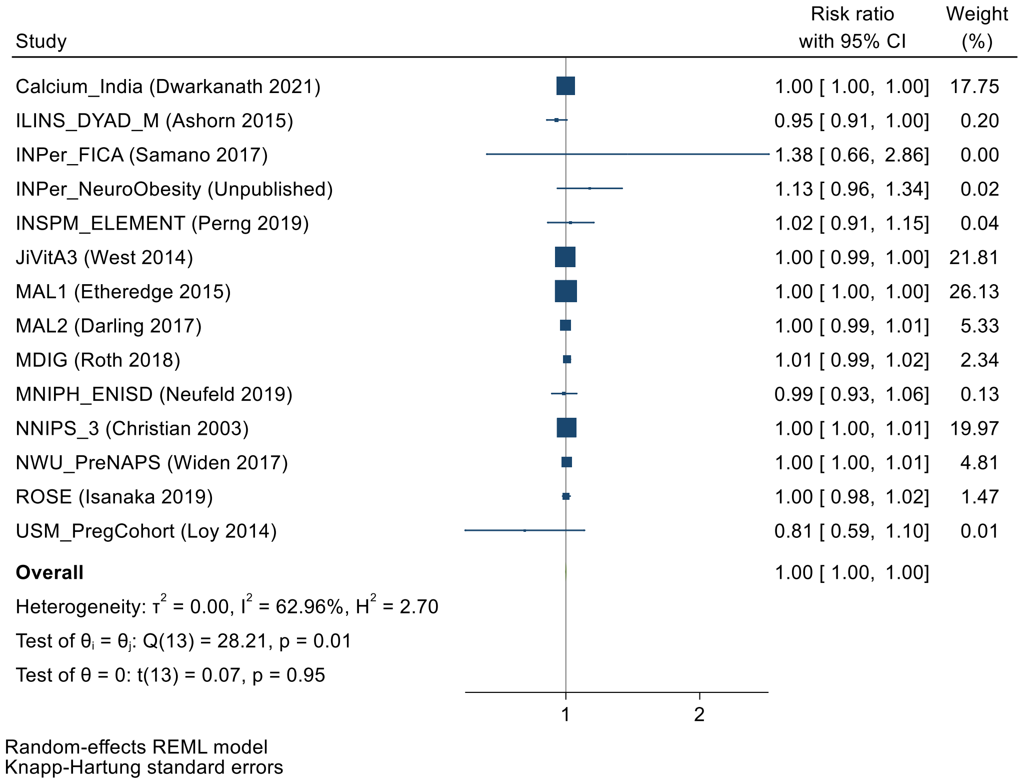

Supplementary Figure 35. Forest plot of association between moderately inadequate gestational weight gain and likelihood of currently breastfeeding.

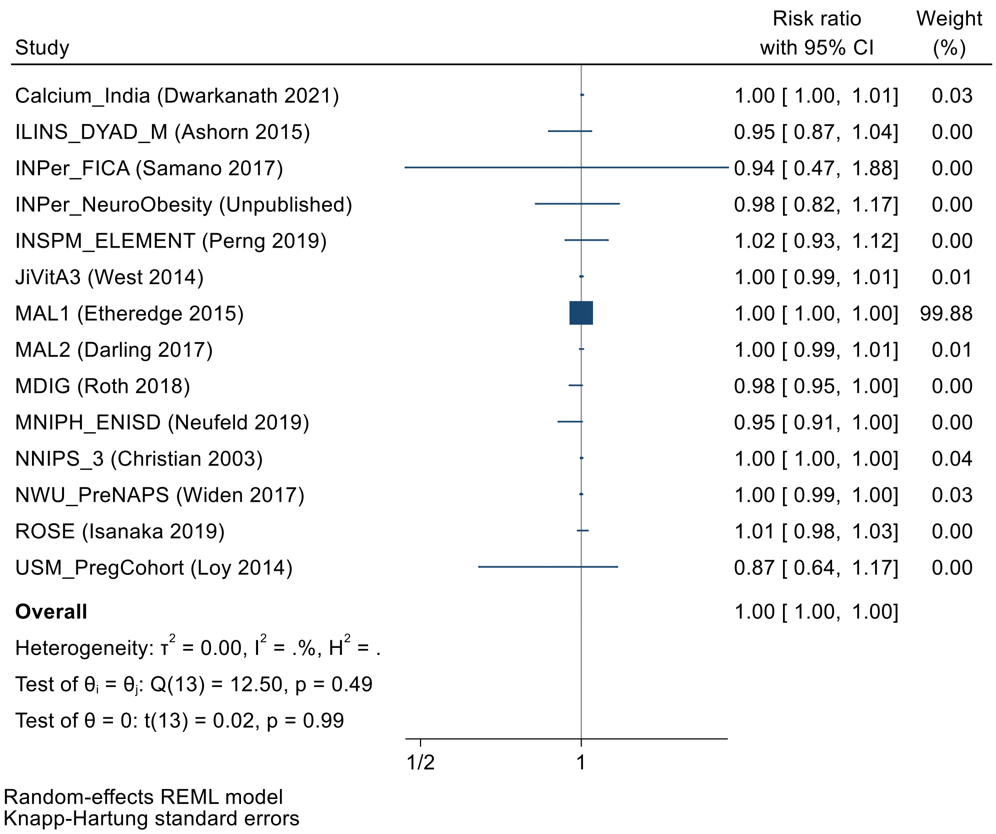

Supplementary Figure 36. Forest plot of association between excessive gestational weight gain and likelihood of currently breastfeeding.

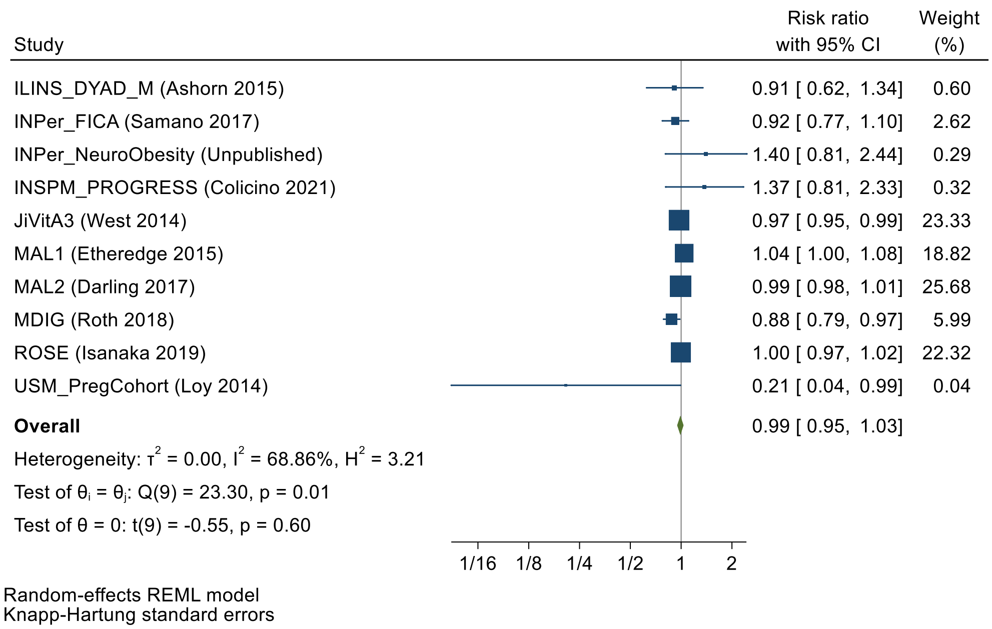

Supplementary Figure 37. Forest plot of association between severely inadequate gestational weight gain and likelihood of currently exclusively breastfeeding.

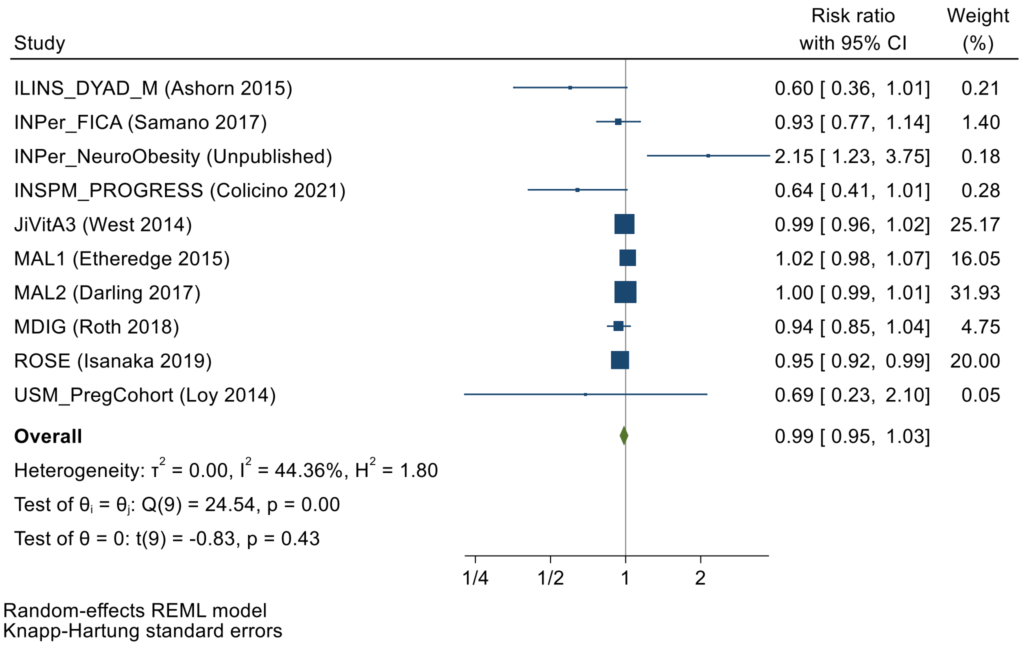

Supplementary Figure 38. Forest plot of association between moderately inadequate gestational weight gain and likelihood of currently exclusively breastfeeding.

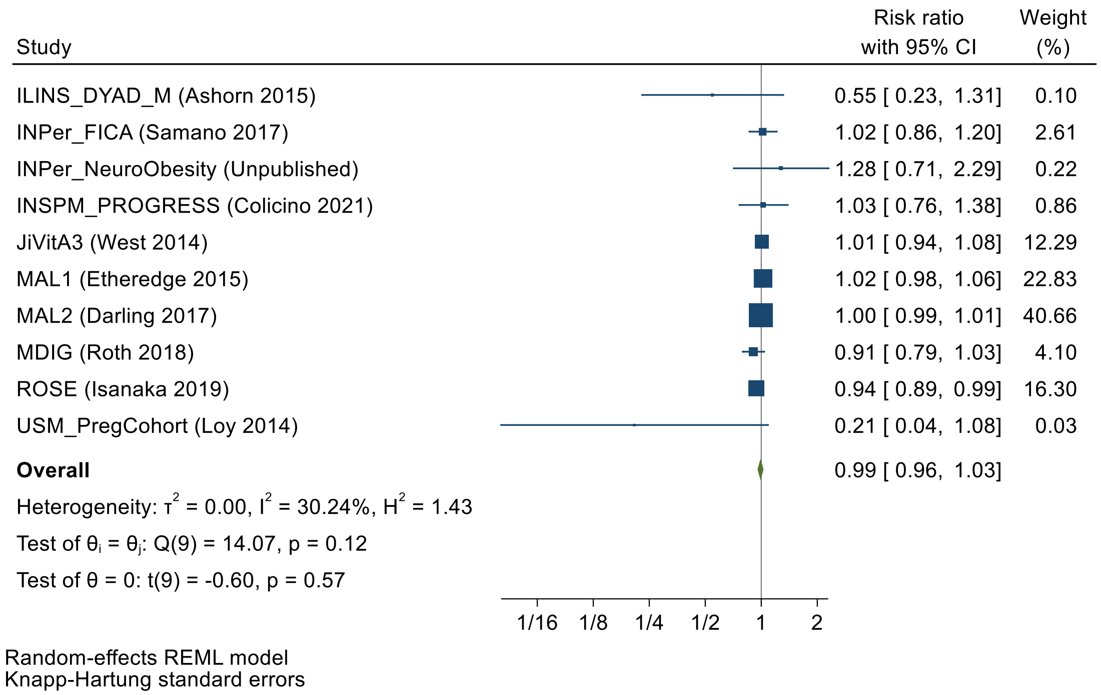

Supplementary Figure 39. Forest plot of association between excessive gestational weight gain and likelihood of currently exclusively breastfeeding.

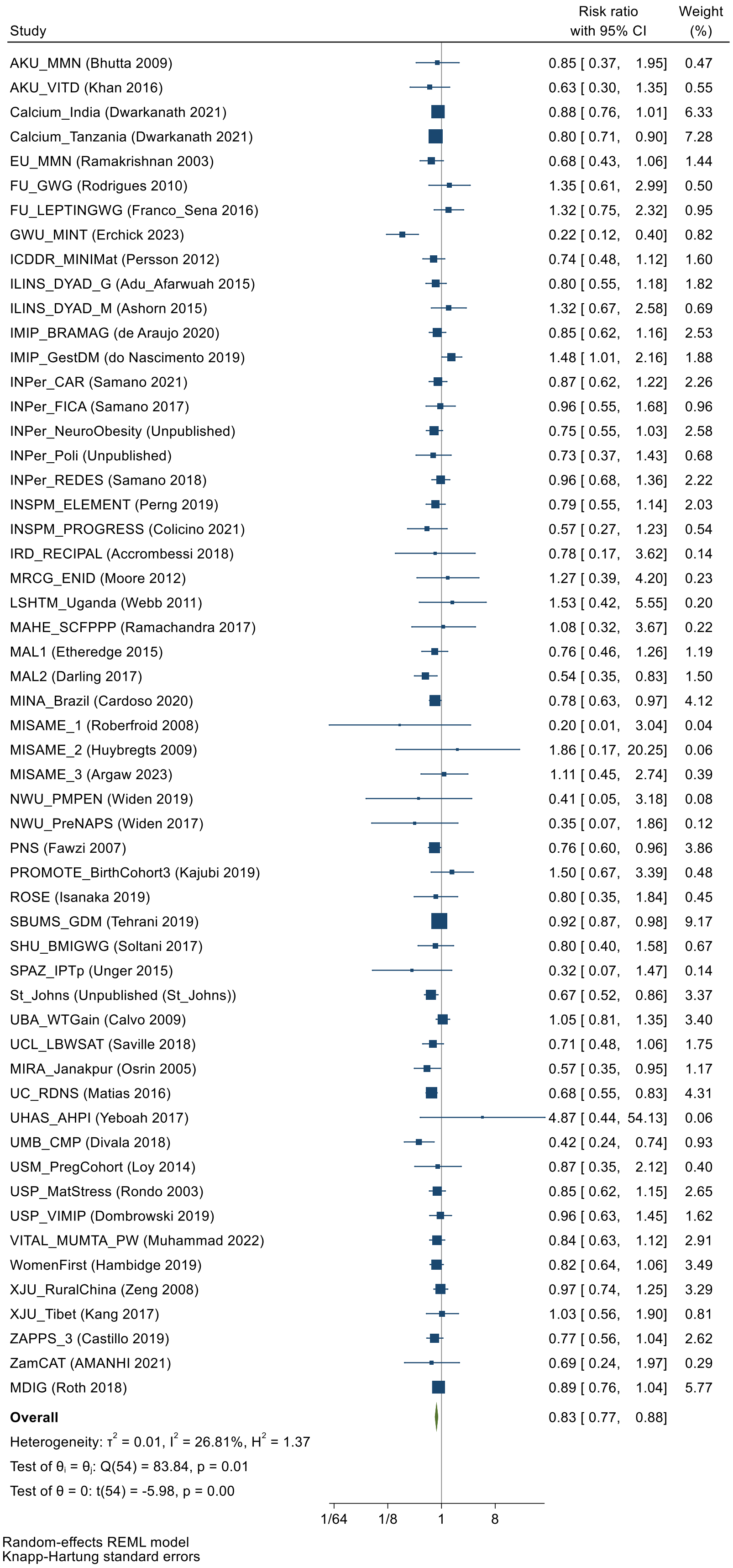

Supplementary Figure 40. Forest plot of association between severely inadequate gestational weight gain and likelihood of cesarean delivery (missing data addressed using multiple imputation).

Study	Risk ratio with 95% CI	Weight (%)
AKU_MMN (Bhutta 2009)	1.05 [0.45, 2.48]	0.33
AKU_VITD (Khan 2016)	0.87 [0.47, 1.60]	0.63
Calcium_India (Dwarkanath 2021)	0.95 [0.88, 1.02]	11.88
Calcium_Tanzania (Dwarkanath 2021)	0.82 [0.73, 0.91]	8.91
EU_MMN (Ramakrishnan 2003)	0.80 [0.52, 1.25]	1.19
FU_GWG (Rodrigues 2010)	1.09 [0.43, 2.72]	0.29
FU_LEPTINGWG (Franco_Sena 2016)	0.84 [0.39, 1.82]	0.40
GWU_MINT (Erchick 2023)	0.85 [0.54, 1.34]	1.12
ICDDR_MINIMat (Persson 2012)	0.96 [0.60, 1.55]	1.02
ILINS_DYAD_G (Adu_Afarwuah 2015)	0.93 [0.61, 1.41]	1.31
ILINS_DYAD_M (Ashorn 2015)	1.52 [0.77, 2.98]	0.52
IMIP_BRAMAG (de Araujo 2020)	0.94 [0.72, 1.23]	2.82
IMIP_GestDM (do Nascimento 2019)	1.39 [0.93, 2.07]	1.41
INPer_CAR (Samano 2021)	0.68 [0.44, 1.05]	1.18
INPer_FICA (Samano 2017)	0.96 [0.60, 1.55]	1.01
INPer_NeuroObesity (Unpublished)	0.75 [0.47, 1.18]	1.09
INPer_Poli (Unpublished)	0.93 [0.46, 1.89]	0.48
INPer_REDES (Samano 2018)	0.92 [0.64, 1.32]	1.68
INSPM_ELEMENT (Perng 2019)	0.89 [0.67, 1.18]	2.61
INSPM_PROGRESS (Colicino 2021)	0.90 [0.64, 1.28]	1.79
IRD_RECIPAL (Accrombessi 2018)	2.22 [0.39, 12.65]	0.08
MRCG_ENID (Moore 2012)	0.24 [0.00, 598.94]	0.00
LSHTM_Uganda (Webb 2011)	1.03 [0.56, 1.87]	0.66
MAHE_SCFPPP (Ramachandra 2017)	0.29 [0.04, 1.94]	0.07
MAL1 (Etheredge 2015)	0.67 [0.41, 1.12]	0.90
MAL2 (Darling 2017)	0.63 [0.36, 1.11]	0.74
MINA_Brazil (Cardoso 2020)	0.91 [0.74, 1.12]	4.19
MISAME_1 (Roberfroid 2008)	0.91 [0.06, 12.77]	0.04
MISAME_3 (Argaw 2023)	0.99 [0.33, 2.99]	0.20
NWU_PMPEN (Widen 2019)	1.09 [0.18, 6.38]	0.08
NWU_PreNAPS (Widen 2017)	0.65 [0.11, 3.99]	0.07
PNS (Fawzi 2007)	0.70 [0.56, 0.89]	3.61
PROMOTE_BirthCohort3 (Kajubi 2019)	1.06 [0.38, 2.93]	0.23
ROSE (Isanaka 2019)	1.26 [0.57, 2.78]	0.39
SBUMS_GDM (Tehrani 2019)	0.98 [0.92, 1.04]	13.24
SHU_BMIGWG (Soltani 2017)	1.26 [0.61, 2.57]	0.47
SPAZ_IPTp (Unger 2015)	1.00 [0.42, 2.36]	0.32
St_Johns (Unpublished (St_Johns))	0.81 [0.64, 1.01]	3.79
UBA_WTGain (Calvo 2009)	1.05 [0.78, 1.43]	2.29
UCL_LBWSAT (Saville 2018)	0.63 [0.45, 0.88]	1.92
MIRA_Janakpur (Osrin 2005)	0.68 [0.40, 1.13]	0.88
UC_RDNS (Matias 2016)	0.81 [0.65, 1.01]	3.77
UHAS_AHPI (Yeboah 2017)	6.82 [0.60, 77.51]	0.04
UMB_CMP (Divala 2018)	0.71 [0.39, 1.30]	0.65
USM_PregCohort (Loy 2014)	0.42 [0.12, 1.53]	0.15
USP_MatStress (Rondo 2003)	0.73 [0.53, 0.99]	2.24
USP_VIMIP (Dombrowski 2019)	0.94 [0.64, 1.39]	1.52
UZ_MatNutri (Friis 2004)	0.45 [0.08, 2.54]	0.08
VITAL_MUMTA_PW (Muhammad 2022)	0.93 [0.67, 1.27]	2.11
WomenFirst (Hambidge 2019)	0.87 [0.65, 1.16]	2.55
XJU_RuralChina (Zeng 2008)	0.86 [0.62, 1.20]	1.99
XJU_Tibet (Kang 2017)	1.19 [0.64, 2.21]	0.62
ZAPPS_3 (Castillo 2019)	0.80 [0.57, 1.12]	1.87
ZamCAT (AMANHI 2021)	0.83 [0.30, 2.35]	0.23
MDIG (Roth 2018)	0.83 [0.71, 0.97]	6.36
**Overall**	0.88 [0.84, 0.92]	

Heterogeneity: $\tau^2 = 0.00$, $I^2 = 16.99\%$, $H^2 = 1.20$

Test of $\theta_i = \theta_j$: Q(54) = 51.91, p = 0.56

Test of $\theta = 0$: t(54) = -5.99, p = 0.00

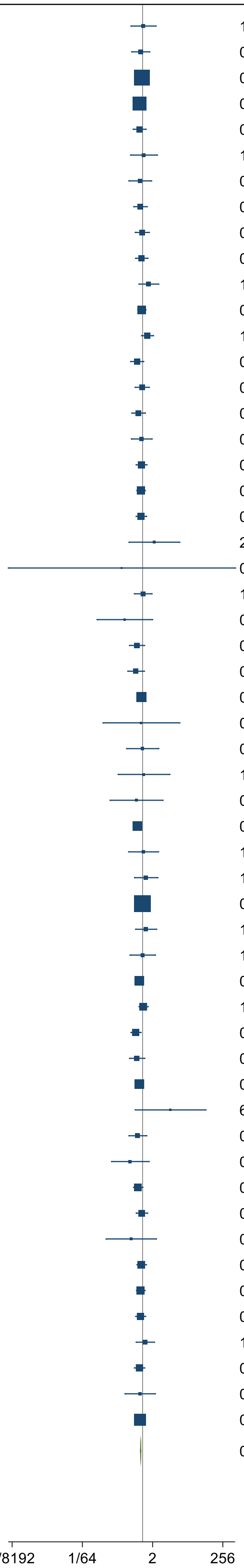

Random-effects REML model
Knapp-Hartung standard errors

Supplementary Figure 41. Forest plot of association between moderately inadequate gestational weight gain and likelihood of cesarean delivery (missing data addressed using multiple imputation).

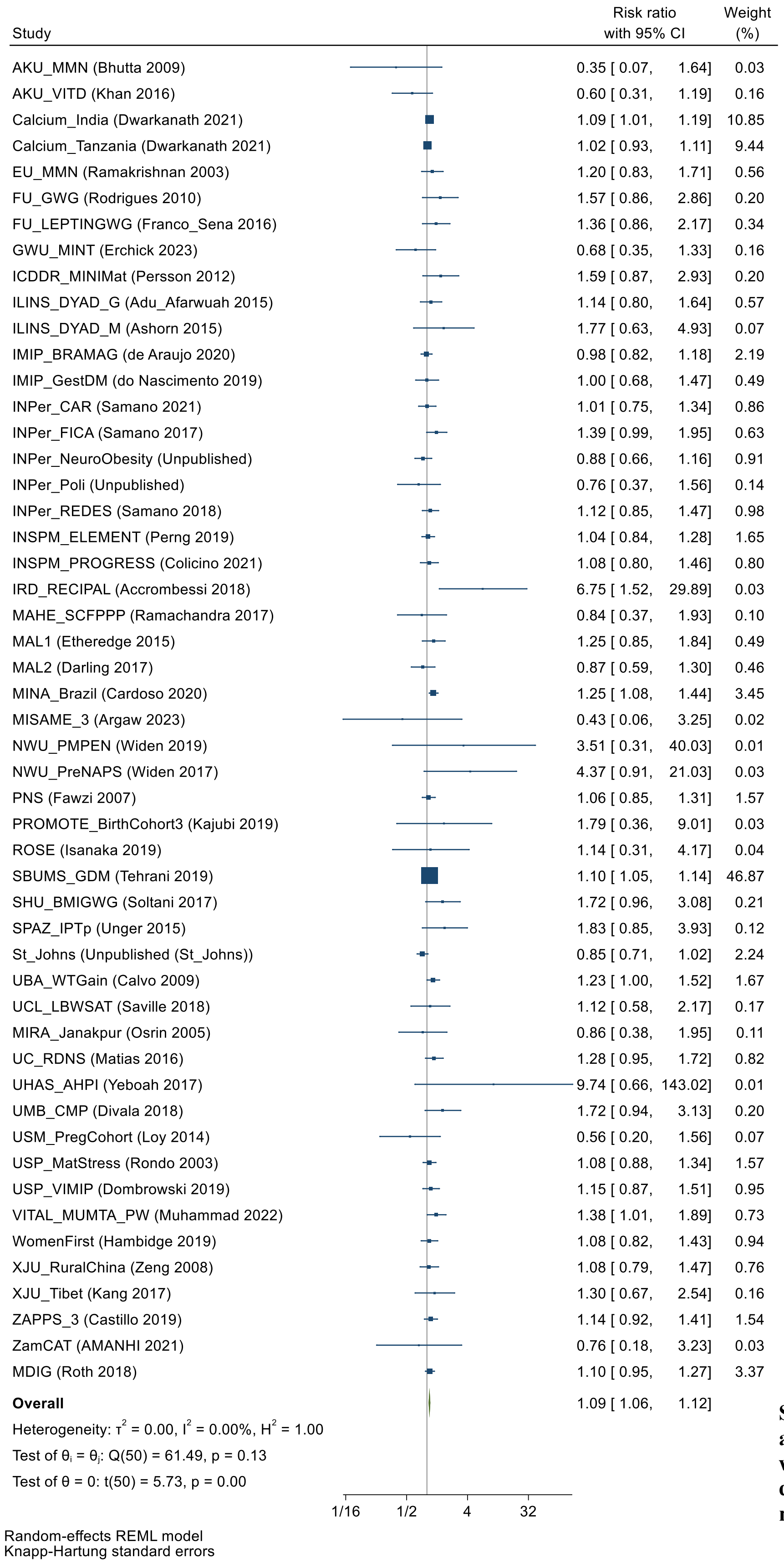

Supplementary Figure 42. Forest plot of association between excessive gestational weight gain and likelihood of cesarean delivery (missing data addressed using multiple imputation).

References

1. Bhutta ZA, Rizvi A, Raza F, Hotwani S, Zaidi S, Hossain SM, et al. A Comparative Evaluation of Multiple Micronutrient and Iron–Folic Acid Supplementation during Pregnancy in Pakistan: Impact on Pregnancy Outcomes. Food Nutr Bull. 2009 Dec;30(4_suppl4):S496–505.

2. Khan FR, Ahmad T, Hussain R, Bhutta ZA. A Randomized Controlled Trial of Oral Vitamin D Supplementation in Pregnancy to Improve Maternal Periodontal Health and Birth Weight. J Int Oral Health. 2016;

3. Dwarkanath P, Muhihi A, Sudfeld CR, Rani S, Duggan CP, Sando MM, et al. Non-inferiority of low-dose compared to standard high-dose calcium supplementation in pregnancy: study protocol for two randomized, parallel group, non-inferiority trials in India and Tanzania. Trials. 2021 Nov 24;22(1):838.

4. Ramakrishnan U, González-Cossío T, Neufeld LM, Rivera J, Martorell R. Multiple micronutrient supplementation during pregnancy does not lead to greater infant birth size than does iron-only supplementation: a randomized controlled trial in a semirural community in Mexico. Am J Clin Nutr. 2003 Mar;77(3):720–5.

5. Rodrigues PL, Costa De Oliveira L, Santos Brito AD, Kac G. Determinant factors of insufficient and excessive gestational weight gain and maternal–child adverse outcomes. Nutrition. 2010 Jun;26(6):617–23.

6. Franco-Sena AB, Rebelo F, Pinto T, Farias DR, Silveira GE, Mendes RH, et al. The effect of leptin concentrations and other maternal characteristics on gestational weight gain is different according to pre-gestational BMI: results from a prospective cohort. BJOG Int J Obstet Gynaecol. 2016 Oct;123(11):1804–13.

7. Erchick DJ, Lama TP, Khatry SK, Katz J, Mullany LC, Zavala E, et al. Supplementation with fortified balanced energy-protein during pregnancy and lactation and its effects on birth outcomes and infant growth in southern Nepal: protocol of a 2×2 factorial randomised trial. BMJ Paediatr Open. 2023 Nov;7(1):e002229.

8. Persson LA, Arifeen S, Ekstrom EC, Rasmussen KM, Frongillo EA, Yunus M. Effects of prenatal micronutrient and early food supplementation on maternal hemoglobin, birth weight, and infant mortality among children in Bangladesh: the MINIMat randomized trial. JAMA. 2012 May 16;307(19):2050–9.

9. Adu-Afarwuah S, Lartey A, Okronipa H, Ashorn P, Zeilani M, Peerson JM, et al. Lipid-based nutrient supplement increases the birth size of infants of primiparous women in Ghana. Am J Clin Nutr. 2015 Apr;101(4):835–46.

10. Ashorn P, Alho L, Ashorn U, Cheung YB, Dewey KG, Harjunmaa U, et al. The impact of lipid-based nutrient supplement provision to pregnant women on newborn size in rural Malawi: a randomized controlled trial. Am J Clin Nutr. 2015 Feb;101(2):387–97.

11. De Araújo CAL, Ray JG, Figueiroa JN, Alves JG. BRAzil magnesium (BRAMAG) trial: a double-masked randomized clinical trial of oral magnesium supplementation in pregnancy. BMC Pregnancy Childbirth. 2020 Dec;20(1):234.

12. Do Nascimento GR, Borges MDC, Figueiroa JN, Alves LV, Alves JG. Physical activity pattern in early pregnancy and gestational diabetes mellitus risk among low-income women: A prospective cross-sectional study. SAGE Open Med. 2019 Jan;7:205031211987592.

13. Sámano R, Ortiz-Hernández L, Martínez-Rojano H, Nájera-Medina O, Chico-Barba G, Sánchez-Jiménez B, et al. Disordered Eating Behaviors Are Associated with Gestational Weight Gain in Adolescents. Nutrients. 2021 Sep 13;13(9):3186.

14. Sámano R, Martínez-Rojano H, Chico-Barba G, Godínez-Martínez E, Sánchez-Jiménez B, Montiel-Ojeda D, et al. Serum Concentration of Leptin in Pregnant Adolescents Correlated with Gestational Weight Gain, Postpartum Weight Retention and Newborn Weight/Length. Nutrients. 2017 Sep 27;9(10):1067.

15. Chico-Barba G, Sámano R, Martínez-Rojano H, Morales-Hernández RM, Barrientos-Galeana E, Luna-Hidalgo A, et al. Total Gestational Weight Gain Is Explained by Leptin and Body Fat, Regardless of Pre-Pregnancy Body Mass Index and Other Adipokines, in Mexican Adolescents. Nutrients. 2024 Jan;16(13):2147.

16. Sámano R, Chico-Barba G, Martínez-Rojano H, Godínez E, Rodríguez-Ventura AL, Ávila-Koury G, et al. Pre-pregnancy body mass index classification and gestational weight gain on neonatal outcomes in adolescent mothers: A follow-up study. Simeoni U, editor. PLOS ONE. 2018 Jul 12;13(7):e0200361.

17. Perng W, Tamayo-Ortiz M, Tang L, Sánchez BN, Cantoral A, Meeker JD, et al. Early Life Exposure in Mexico to ENvironmental Toxicants (ELEMENT) Project. BMJ Open. 2019 Aug;9(8):e030427.

18. Colicino E, De Water E, Just AC, Navarro E, Pedretti NF, McRae N, et al. Prenatal urinary concentrations of phthalate metabolites and behavioral problems in Mexican children: The Programming Research in Obesity, Growth Environment and Social Stress (PROGRESS) study. Environ Res. 2021 Oct;201:111338.

19. Accrombessi M, Yovo E, Cottrell G, Agbota G, Gartner A, Martin-Prevel Y, et al. Cohort profile: effect of malaria in early pregnancy on fetal growth in Benin (RECIPAL preconceptional cohort). BMJ Open. 2018 Jan;8(1):e019014.

20. Katz J, Khatry SK, Shrestha L, Summers A, Visscher MO, Sherchand JB, et al. Impact of topical applications of sunflower seed oil on neonatal mortality and morbidity in southern Nepal: a community-based, cluster-randomised trial. BMJ Glob Health. 2024 Feb 29;9(2):e013691.

21. West KP Jr, Shamim AA, Mehra S, Labrique AB, Ali H, Shaikh S, et al. Effect of Maternal Multiple Micronutrient vs Iron–Folic Acid Supplementation on Infant Mortality and Adverse Birth Outcomes in Rural Bangladesh: The JiVitA-3 Randomized Trial. JAMA. 2014 Dec 24;312(24):2649–58.

22. Moore SE, Fulford AJ, Darboe MK, Jobarteh ML, Jarjou LM, Prentice AM. A randomized trial to investigate the effects of pre-natal and infant nutritional supplementation on infant immune development in rural Gambia: the ENID trial: Early Nutrition and Immune Development. BMC Pregnancy Childbirth. 2012 Dec;12(1):107.

23. Webb EL, Mawa PA, Ndibazza J, Kizito D, Namatovu A, Kyosiimire-Lugemwa J, et al. Effect of single-dose anthelmintic treatment during pregnancy on an infant's response to immunisation and on susceptibility to infectious diseases in infancy: a randomised, double-blind, placebo-controlled trial. Lancet. 2011 Jan 1;377(9759):52–62.

24. Ramachandra P, Kumar P, Kamath A, Maiya AG. Do Structural Changes of the Foot Influence Plantar Pressure Patterns During Various Stages of Pregnancy and Postpartum? Foot Ankle Spec. 2017 Dec;10(6):513–9.

25. Etheredge AJ, Premji Z, Gunaratna NS, Abioye AI, Aboud S, Duggan C, et al. Iron Supplementation in Iron-Replete and Nonanemic Pregnant Women in Tanzania: A Randomized Clinical Trial. JAMA Pediatr. 2015 Oct 1;169(10):947–55.

26. Darling AM, Mugusi FM, Etheredge AJ, Gunaratna NS, Abioye AI, Aboud S, et al. Vitamin A and Zinc Supplementation Among Pregnant Women to Prevent Placental Malaria: A Randomized, Double-Blind, Placebo-Controlled Trial in Tanzania. Am J Trop Med Hyg. 2017 Apr;96(4):826–34.

27. Roth DE, Morris SK, Zlotkin S, Gernand AD, Ahmed T, Shanta SS, et al. Vitamin D Supplementation in Pregnancy and Lactation and Infant Growth. N Engl J Med. 2018 Aug 9;379(6):535–46.

28. Cardoso MA, Matijasevich A, Malta MB, Lourenco BH, Gimeno SGA, Ferreira MU, et al. Cohort profile: the Maternal and Child Health and Nutrition in Acre, Brazil, birth cohort study (MINA-Brazil). BMJ Open. 2020 Feb;10(2):e034513.

29. Roberfroid D, Huybregts L, Lanou H, Henry MC, Meda N, Menten J, et al. Effects of maternal multiple micronutrient supplementation on fetal growth: a double-blind randomized controlled trial in rural Burkina Faso. Am J Clin Nutr. 2008 Nov;88(5):1330–40.

30. Huybregts L, Roberfroid D, Lanou H, Menten J, Meda N, Van Camp J, et al. Prenatal food supplementation fortified with multiple micronutrients increases birth length: a randomized controlled trial in rural Burkina Faso. Am J Clin Nutr. 2009 Dec;90(6):1593–600.

31. Argaw A, Kok B de, Toe LC, Hanley-Cook G, Dailey-Chwalibóg T, Ouédraogo M, et al. Fortified balanced energy–protein supplementation during pregnancy and lactation and infant growth in rural Burkina Faso: A 2 × 2 factorial individually randomized controlled trial. PLOS Med. 2023 Feb 6;20(2):e1004186.

32. Neufeld LM, García-Guerra A, Quezada AD, Théodore F, Bonvecchio Arenas A, Islas CD, et al. A Fortified Food Can Be Replaced by Micronutrient Supplements for Distribution in a Mexican Social Protection Program Based on Results of a Cluster-Randomized Trial and Costing Analysis. J Nutr. 2019 Dec 1;149(Suppl 1):2302S-2309S.

33. Christian P. Effects of alternative maternal micronutrient supplements on low birth weight in rural Nepal: double blind randomised community trial. BMJ. 2003 Mar 15;326(7389):571–571.

34. Widen EM, Tsai I, Collins SM, Wekesa P, China J, Krumdieck N, et al. HIV infection and increased food insecurity are associated with adverse body composition changes among pregnant and lactating Kenyan women. Eur J Clin Nutr. 2019 Mar;73(3):474–82.

35. Widen EM, Collins SM, Khan H, Biribawa C, Acidri D, Achoko W, et al. Food insecurity, but not HIV-infection status, is associated with adverse changes in body composition during lactation in Ugandan women of mixed HIV status. Am J Clin Nutr. 2017 Feb;105(2):361–8.

36. Fawzi WW, Msamanga GI, Urassa W, Hertzmark E, Petraro P, Willett WC, et al. Vitamins and Perinatal Outcomes among HIV-Negative Women in Tanzania. N Engl J Med. 2007 Apr 5;356(14):1423–31.

37. Kajubi R, Ochieng T, Kakuru A, Jagannathan P, Nakalembe M, Ruel T, et al. Monthly sulfadoxine–pyrimethamine versus dihydroartemisinin–piperaquine for intermittent preventive treatment of malaria in pregnancy: a double-blind, randomised, controlled, superiority trial. The Lancet. 2019 Apr;393(10179):1428–39.

38. Isanaka S, Kodish SR, Mamaty AA, Guindo O, Zeilani M, Grais RF. Acceptability and utilization of a lipid-based nutrient supplement formulated for pregnant women in rural Niger: a multi-methods study. BMC Nutr. 2019 Dec;5(1):34.

39. Taneja S, Chowdhury R, Dhabhai N, Upadhyay RP, Mazumder S, Sharma S, et al. Impact of a package of health, nutrition, psychosocial support, and WaSH interventions delivered during preconception, pregnancy, and early childhood periods on birth outcomes and on linear growth at 24 months of age: factorial, individually randomised controlled trial. BMJ. 2022 Oct 26;379:e072046.

40. Ramezani Tehrani F, Gulf Study Cooperative Research Group, Behboudi-Gandevani S, Abedini M, Soleymani-Dodaran M, Khalili D, et al. Cost effectiveness of different screening strategies for gestational diabetes mellitus screening: study protocol of a randomized community non-inferiority trial. Diabetol Metab Syndr. 2019 Dec;11(1):106.

41. Soltani H, Lipoeto NI, Fair FJ, Kilner K, Yusrawati Y. Pre-pregnancy body mass index and gestational weight gain and their effects on pregnancy and birth outcomes: a cohort study in West Sumatra, Indonesia. BMC Womens Health. 2017 Dec;17(1):102.

42. Unger HW, Ome-Kaius M, Wangnapi RA, Umbers AJ, Hanieh S, Suen CSLW, et al. Sulphadoxine-pyrimethamine plus azithromycin for the prevention of low birthweight in Papua New Guinea: a randomised controlled trial. BMC Med. 2015 Dec;13(1):9.

43. Calvo EB, López LB, Balmaceda YDV, Poy MS, González C, Quintana L, et al. Reference charts for weight gain and body mass index during pregnancy obtained from a healthy cohort. J Matern Fetal Neonatal Med. 2009 Jan;22(1):36–42.

44. Saville NM, Shrestha BP, Style S, Harris-Fry H, Beard BJ, Sen A, et al. Impact on birth weight and child growth of Participatory Learning and Action women's groups with and without transfers of food or cash during pregnancy: Findings of the low birth weight South Asia cluster-randomised controlled trial (LBWSAT) in Nepal. Van Wouwe JP, editor. PLOS ONE. 2018 May 9;13(5):e0194064.

45. Osrin D, Vaidya A, Shrestha Y, Baniya RB, Manandhar DS, Adhikari RK, et al. Effects of antenatal multiple micronutrient supplementation on birthweight and gestational duration in Nepal: double-blind, randomised controlled trial. The Lancet. 2005 Mar;365(9463):955–62.

46. Matias SL, Mridha MK, Paul RR, Hussain S, Vosti SA, Arnold CD, et al. Prenatal Lipid-Based Nutrient Supplements Affect Maternal Anthropometric Indicators Only in Certain Subgroups of Rural Bangladeshi Women. J Nutr. 2016 Sep;146(9):1775–82.

47. Yeboah FA, Ngala RA, Bawah AT, Asare-Anane H, Alidu H, Hamid AWM, et al. Adiposity and hyperleptinemia during the first trimester among pregnant women with preeclampsia. Int J Womens Health. 2017 Jun;Volume 9:449–54.

48. Divala TH, Mungwira RG, Mawindo PM, Nyirenda OM, Kanjala M, Ndaferankhande M, et al. Chloroquine as weekly chemoprophylaxis or intermittent treatment to prevent malaria in pregnancy in Malawi: a randomised controlled trial. Lancet Infect Dis. 2018 Oct 1;18(10):1097–107.

49. Loy S, Jan Mohamed HJ. The Universiti Sains Malaysia Pregnancy Cohort Study: Maternal-infant Adiposity Development until the First Year of Life. Health Environ J. 2014;5(1).

50. Rondó PHC, Ferreira RF, Nogueira F, Ribeiro MCN, Lobert H, Artes R. Maternal psychological stress and distress as predictors of low birth weight, prematurity and intrauterine growth retardation. Eur J Clin Nutr. 2003 Feb 1;57(2):266–72.

51. Dombrowski JG, Souza RMD, Lima FA, Bandeira CL, Murillo O, Costa DDS, et al. Association of Malaria Infection During Pregnancy With Head Circumference of Newborns in the Brazilian Amazon. JAMA Netw Open. 2019 May 3;2(5):e193300.

52. Friis H, Gomo E, Nyazema N, Ndhlovu P, Krarup H, Kæstel P, et al. Effect of multimicronutrient supplementation on gestational length and birth size: a randomized, placebo-controlled, double-blind effectiveness trial in Zimbabwe. Am J Clin Nutr. 2004 Jul;80(1):178–84.

53. Muhammad A, Fazal ZZ, Baloch B, Nisar I, Jehan F, Shafiq Y. Nutritional support and prophylaxis of azithromycin for pregnant women to improve birth outcomes in peri-urban slums of Karachi, Pakistan—a protocol of multi-arm assessor-blinded randomized controlled trial (Mumta PW trial). Trials. 2022 Dec;23(1):2.

54. Hambidge KM, Westcott JE, Garcés A, Figueroa L, Goudar SS, Dhaded SM, et al. A multicountry randomized controlled trial of comprehensive maternal nutrition supplementation initiated before conception: the Women First trial. Am J Clin Nutr. 2019 Feb;109(2):457–69.

55. Zeng L, Cheng Y, Dang S, Yan H, Dibley MJ, Chang S, et al. Impact of micronutrient supplementation during pregnancy on birth weight, duration of gestation, and perinatal mortality in rural western China: double blind cluster randomised controlled trial. BMJ. 2008 Nov 7;337:a2001.

56. Kang Y, Dang S, Zeng L, Wang D, Li Q, Wang J, et al. Multi-micronutrient supplementation during pregnancy for prevention of maternal anaemia and adverse birth outcomes in a high-altitude area: a prospective cohort study in rural Tibet of China. Br J Nutr. 2017 Sep 28;118(6):431–40.

57. Castillo MC, Fuseini NM, Rittenhouse K, Price JT, Freeman BL, Mwape H, et al. The Zambian Preterm Birth Prevention Study (ZAPPS): Cohort characteristics at enrollment. Gates Open Res. 2019;2:25.

58. Group TA for M and NHI (AMANHI) GAS. Simplified models to assess newborn gestational age in low-middle income countries: findings from a multicountry, prospective cohort study. BMJ Glob Health. 2021 Sep 1;6(9):e005688.
